# Coevolution of visual behaviour, the material world and social complexity, depicted by the eye-tracking of archaeological objects in humans

**DOI:** 10.1038/s41598-019-39661-w

**Published:** 2019-03-08

**Authors:** Felipe Criado-Boado, Diego Alonso-Pablos, Manuel J. Blanco, Yolanda Porto, Anxo Rodríguez-Paz, Elena Cabrejas, Elena del Barrio-Álvarez, Luis M. Martínez

**Affiliations:** 1 Institute of Heritage Sciences (Incipit), Spanish National Research Council (CSIC), Avenida de Vigo s/n°, 15705 Santiago de Compostela, Spain; 2 Institute of Neurosciences (IN), Spanish National Research Council (CSIC), Universidad Miguel Hernández (UMH), Campus de San Juan, Sant Joan d’Alacant, Alicante, Spain; 3 Laboratory of Perception, Faculty of Psychology, University of Santiago de Compostela (USC), Rúa Xosé María Suárez Núñez, s/n, Campus Vida, 15782 Santiago de Compostela, Spain

## Abstract

We live in a cluttered visual world that is overflowing with information, the continuous processing of which would be a truly daunting task. Nevertheless, our brains have evolved to select which part of a visual scene is to be prioritized and analysed in detail, and which parts can be discarded or analysed at a later stage. This selection is in part determined by the visual stimuli themselves, and is known as “selective attention”, which, in turn, determines how we explore and interact with our environment, including the distinct human artefacts produced in different socio-cultural contexts. Here we hypothesize that visual responses and material objects should therefore co-evolve to reflect changes in social complexity and culture throughout history. Using eye-tracking, we analysed the eye scan paths in response to prehistoric pottery ranging from the Neolithic through to the Iron Age (ca 6000–2000 BP), finding that each ceramic style caused a particular pattern of visual exploration. Horizontal movements become dominant in earlier periods, while vertical movements are more frequent in later periods that were marked by greater social complexity.

## Introduction

This research, by testing how visual cognition is affected by different sorts of archaeological pottery styles belonging to different chronologies and social conditions, provides direct evidence that cognition is not only in our mind, but underpinned in some way by the world around us. This idea has been previously suggested by numerous philosophers and cognitive scientists, but has never been properly tested experimentally. Our work provides clear evidence of a strong correlation between the evolution over time of the social structure of a community and the way in which people interact with and cognitively interpret the world around. Specifically, the visual behaviour, that is, the patterns of eye movements that we produce by freely exploring a chronological series of ceramics, follows the same evolutionary trend as the social structure; and they do so because the producers of these ceramics have progressively, and in parallel, modified the visual structure, mainly the decoration, of the artifacts characterizing each successive cultural periods. Thus, the visual saliency, as measured using the most current algorithms, of each pottery style produces a distinct visual response.

Nowadays there is an increasing interest in the relationship between cognition and the material structure of the environment^1^. Unembodied theories, i.e. theories proposing that thoughts are purely conceptual, assume that cognitive representations are wholly abstract in nature and do not require a direct relationship with the nature of the sensory and motor information to which they refer^2,3^. On the other hand, the most radical embodiment theories propose that the content of cognitive representations is simply a result of the sensory and motor information brought about by the material structure of the environment itself, which would therefore be merely internalized and not transduced^3–6^. While the former view predicts that there is a clearly defined interface between the environment and neural processes, the latter suggests that rather than being something that occurs in the brain alone, cognition is an ongoing, relational process between the brain and its environment^3^. Under these premises, the world and cognition, being inextricably interconnected, would form an actual integrated state, constantly feeding off of each other while directing our behavioral repertoire^4,7,8^. This would also be true for the human-made and transformed environment (constituted by objects, buildings, landscapes, and land-use). Therefore, the material structure of the world and the constituent elements of human cognition should co-evolve as the social structure and cultural context develop over time.

To investigate these issues, we used a collection of prehistoric pottery in which each pot characterizes a specific ceramic style and the environmental context (see Supplementary Information SI1 and SI2), and as a behavioral readout, the patterns of spontaneous exploratory eye movements they motivated, which were recorded by eye-tracking^9–13^. The design of prehistoric pottery is unanimously acknowledged as a clear indicator of socio-cultural identity, as it quite distinctly exemplifies the material style and cultural context of each particular epoch^14–18^. Therefore, using these prehistoric artifacts allows us to compare societies through time and space cross-culturally, by assessing the particular behavioral patterns in response to objects from different cultural and temporal contexts. Given that the basic functional mechanisms of the brain are similar today and in the recent past, the differential influence of artifacts on observers should not be affected by using a sample of a current population.

Using this approach, we examined two different but related hypotheses: firstly, to consider whether or not the material configuration of the object (i.e., the material style it represents) actually imposes a strong bias on our visual behaviour. If confirmed, this would suggest, as an embodied approach would hold, that the mental representation of the pot, rather than being an independent abstraction, actively traces and includes the object, its materiality and shape. The second hypothesis goes a little further in its proposition, in line with a number of previous archaeological conjectures^19–21^. Namely that there was through time an evolution of the material structure of the pots and the visual exploratory patterns they evoke, that parallels changes in other characteristics of the societies to which each material style belongs, including their degree of social complexity^22^.

To test these hypotheses, we designed an experimental protocol that included 15 pots (Fig. Extended Data 1 -ED further on), representative of five different material styles belonging to five distinct chrono-cultural periods spanning from 6000 to 2000 BP (Fig. ED2-6 deconstruct the technological chain –ie. the set of different technological options to mould a pot^23^, of each material style, thereby showing how the analysed pots are representative of the whole style; moreover, any specialist on prehistoric pottery will easily identity the style and chronology of each pot). The pots were all found in the same geographic area in Northwestern Iberia (Fig. ED7) and from societies with increasing degrees of complexity and social hierarchy (Table ED1 and Methods). Despite the fact that we only chose pots from a specific geographic region (NW Iberia), they all correspond to material styles characterizing the evolution of ceramics in Western Europe as a whole^24^. The Bell Beaker pottery (style 3 of our experiment), for instance, is a very homogeneous phenomenon ranging from southern Scandinavia to Morocco and from the European Atlantic façade all the way to the Danube valley^25–28^; its wide distribution throughout Europe has recently been explained as the result of cultural diffusion between certain areas (viz. Iberia and Central Europe), and migrations and population replacement in others (viz. Britain)^29^. Moreover, the other styles can either be found over a vast area^30^ (style 2), or have similar features to other coeval styles at a macro-regional level (styles 1^31^, styles 4 and 5^32^). These 15 pots, our stimuli, were presented as flat photos (Experiment 1, Figs ED1, ED8) and as drawings (Experiment 2, Fig. ED9), as explained in “Methods, Experimental Process”.

## Results

**Figure 1:**
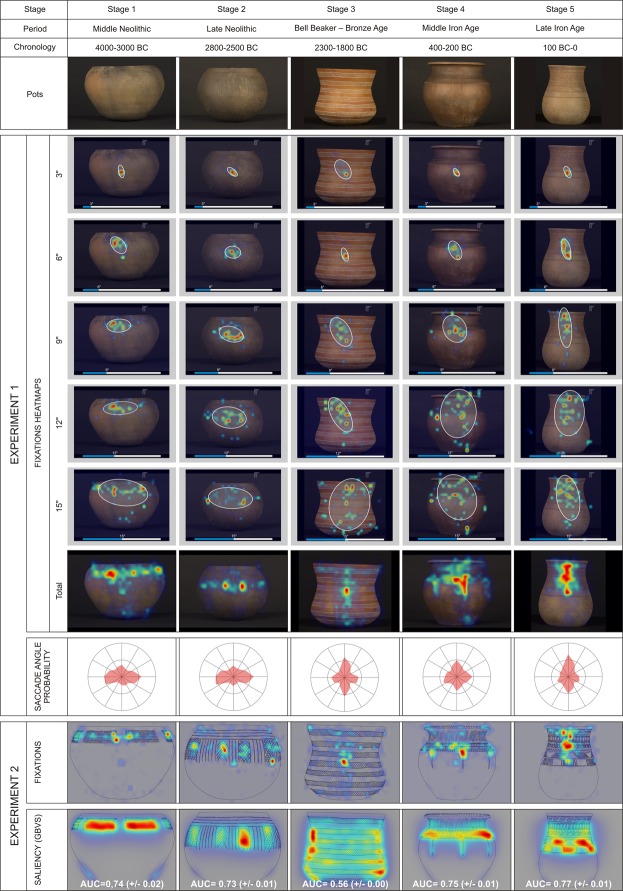
This shows the preference for horizontal or vertical exploration presented by each pot in Exp 1 and presents it as a temporary sequence of images. This information completes Vi (Fig. 2) by showing the interrelationship between the saccades directions and the topological distribution of the fixations density (heat maps). Not only are the visual attention centers in the decorated parts of each pot different, but attending to the shape and internal articulation of the decorative pattern, so is the visual behaviour. The ellipse in each image represents the location of 70% of the positions that were most visited during this time period (with intervals of 500 ms; see Methods).

**Figure 2:**
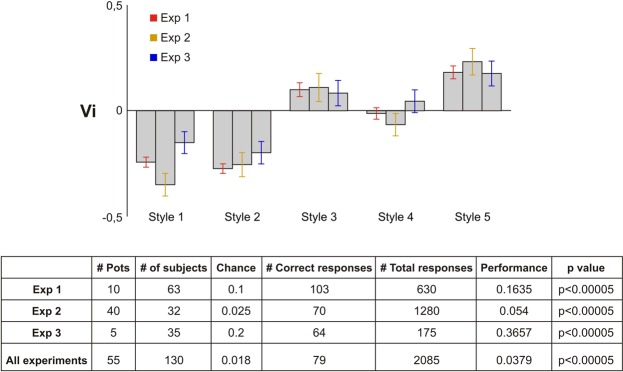
Panel A: The comparison of Vi and AR of the five pots shows that while AR slightly increases, the effect of decoration imposes in some cases (styles 1 and 2) a horizontal pattern of visual movements and a vertical one in other cases (styles 3 to 5). As the Vi of the visual response of photos and drawings is similar (Fig. ED15), Experiments 2 and 3 were only carried out on drawings. The Kruskal-Wallis analysis did not detect any differences between the images of Exp 1 and their drawings in Experiment 2. Hstat = 0.14, well below the 5.99 value for α = 0.05 for a distribution of 2 degrees of freedom, as a result of which we fail to reject H0. However, when considering the differences between the 5 pots Hstat = 12.833 which is well above 10.2, the critical value for α = 0.01 for a 5 × 3 case. We therefore conclude that at least one of the pots differs from the others. Kendall’s correlation coefficient is 0.8 between Exp1 and Exp 2; 1 between Exp1 and Exp3 (significant for a α = 0.01); and 0.8 between Exp2 and Exp3. Panel B: LDA of Vi makes it possible to predict the pot under exploration after the eye movements of each participant.

Figure 1 presents a summary of our results showing that the pattern of spontaneous exploratory eye movements gradually transits from the earliest to the latest pots. We quantified how the experimental subjects explored the different pots using an *ad hoc* index (index of verticality, Vi) that calculates the eye scan paths computed in the X and the Y axes (Methods). Figure 2A shows how, in the three experiments, we found a significant upward trend for the chronology of the pottery. While Pot 1 consistently induced a pattern of visual exploration that was clearly predominated by horizontal eye movements (negative values of Vi), this horizontal bias decreased progressively until it reversed, reaching the maximum vertical tendency (positive values of Vi) for Pot 5.

We used a linear discriminant analysis based on the Vertical Index (Vi) computed from the eye movements of each participant in order to investigate to what extent we could predict the pots they were viewing in each case (Methods). The performance of this decoder was always significantly higher than chance (Fig. 2B), both in each individual experiment and when considering the aggregated results. Thus, the uninstructed subjects’ spontaneous exploratory free-viewing behaviour conveys significant information about the identity of the pots being examined (Fig. ED17). This analysis confirms that the Vi is very informative about the way each piece is looked at, further proving the preponderant role of the material structure of the pots in the subjects’ visual behaviour.

**Figure 3:**
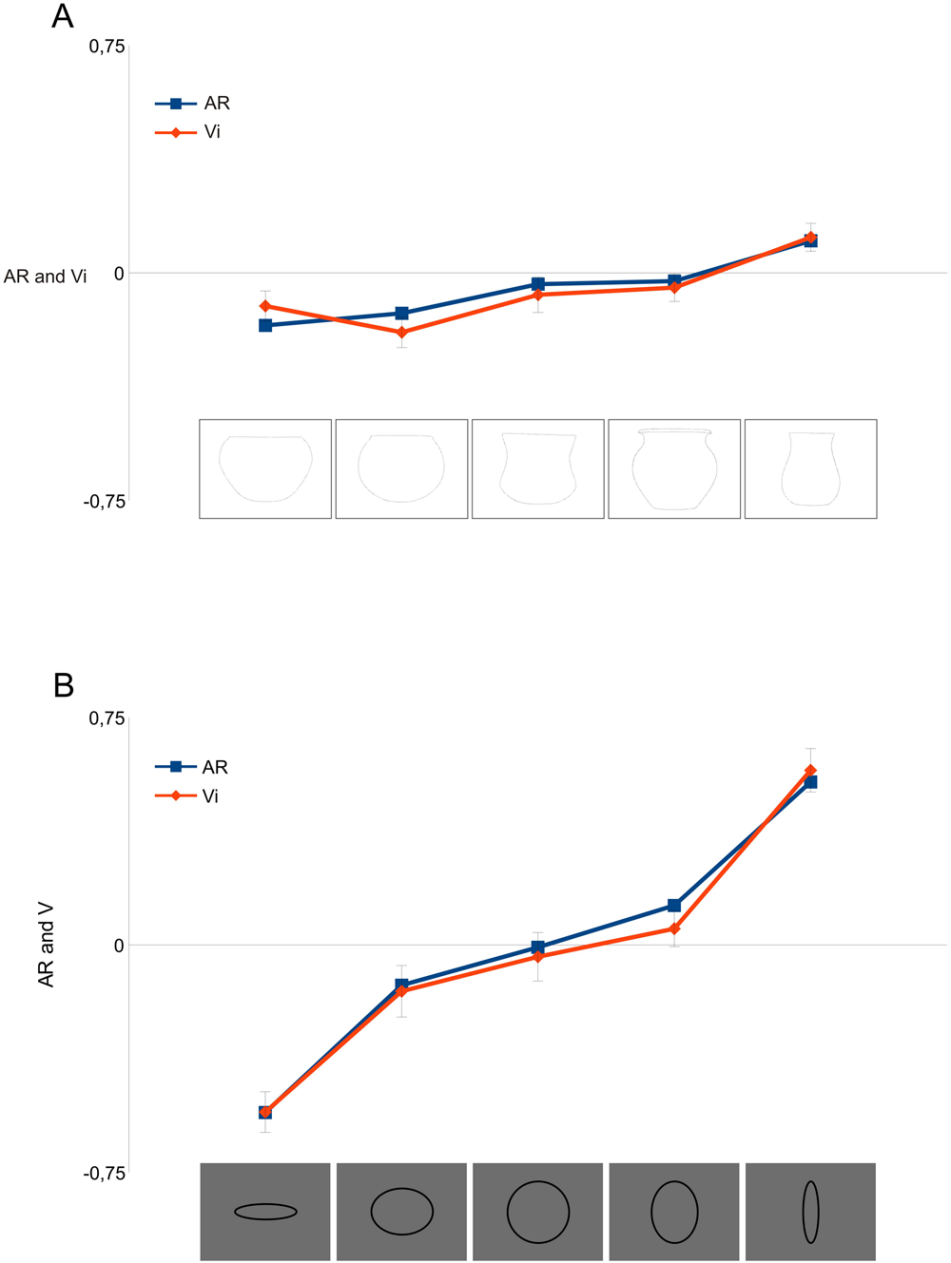
In order to confirm to what extent the visual response depends on the decoration of the pot, in Experiment 2 the decoration of the five experimental pots was removed, presenting them as undecorated, then comparing them with the Vi of geometric figures with different AR. In all cases a strong correlation between AR and Vi is observed.

**Figure 4:**
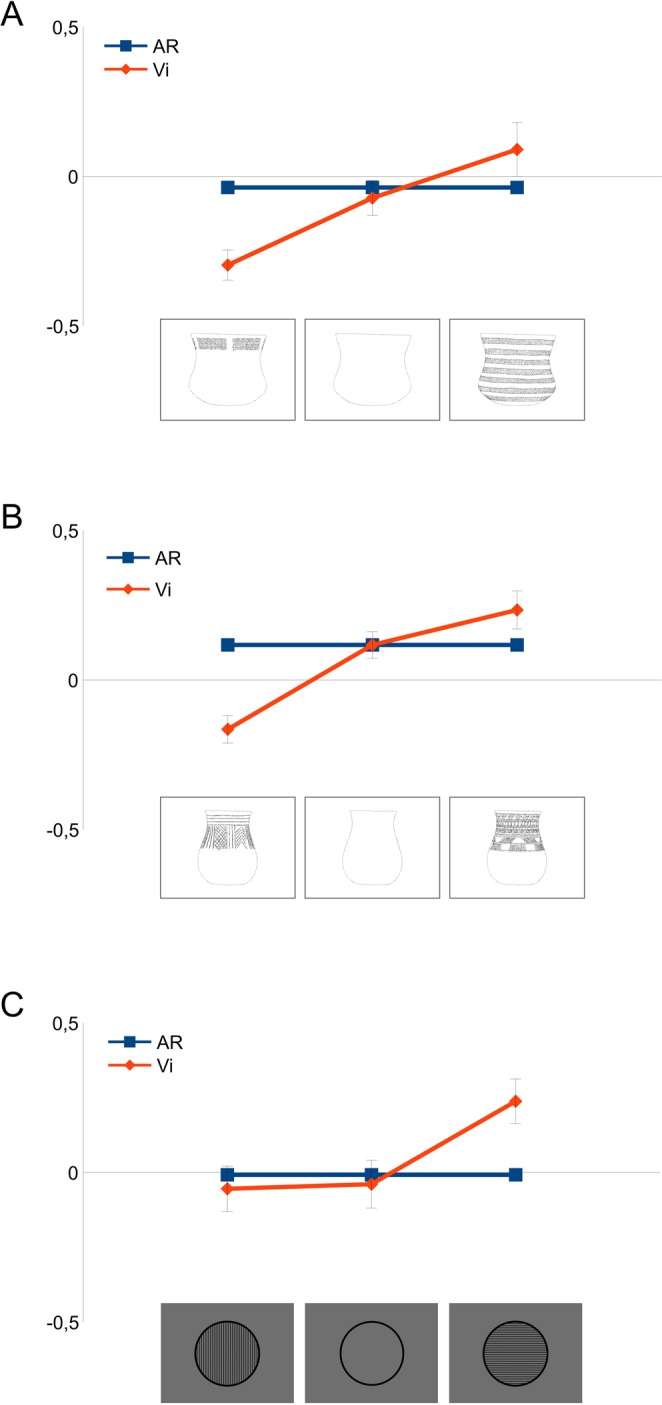
The effect of the decoration can also be observed by exchanging shapes and decorations. (Panel A,B) show the effect produced by introducing a horizontal (left) or vertical (right) exploration decoration into two different pots (no. 3 and 5) that present the highest Vi of our series. (Panel C) shows the same thing when introducing a decoration with horizontal (vertical lines) or vertical (horizontal lines) exploration in a simple geometric figure: a decoration with high Vi on a low AR form verticalizes the visual response.

Our eyes move over visual scenes and objects in order to gather visual information^11^. In principle, this means that we could expect that the overall orientation of the eye movements we make when exploring a visual target is largely determined by its height-to-width aspect ratio (AR). However, a direct comparison of the Vi and the AR of the pots revealed that this was not the case. Even though the AR itself follows an ascending trend, it is the combined effect of the decoration that imposes a more horizontal visual behaviour in some cases, and a more vertical type of visual behaviour in others. The fact that the upward trend in visual behaviour could not be fully explained by the different aspect ratios of the selected pottery is further demonstrated by the data from experiments 2 and 3. In neutral conditions (i.e., only including the outline of the undecorated pots), the AR and the Vi correlate quite well; as they do when we consider the pattern of eye movements produced when subjects explore simple geometric shapes (circles and ellipses) with different aspect ratios (Fig. 3). However, when we introduce decoration into these basic forms, the eye movement patterns are significantly altered (Fig. 4).

## Discussion

Therefore, the subjects’ visual exploratory patterns are determined by a synergistic interaction between the shape (AR) of the pots and their decoration, which is specific to each particular cultural and temporal context.

The correlation between changes in visual exploratory patterns (Vi and the spatial clustering of visual fixations) and the evolution of the spatial distribution of visual features in the material styles of the different chrono-cultural periods, as well as the strong consistency of these results across different subjects, suggests a common underlying process or mechanism in the sense that the visual world (extrinsic factors) has fostered the symmetrical engagement between doing, seeing, and designing (intrinsic factors) throughout history. The decoration of the pots becomes key aspect in closing the loop between these extrinsic and intrinsic factors of human cognition. Consistent with this view, the experts in our sample, who were already familiar with the pieces and therefore had a knowledge of their meaning that far exceeded the knowledge of the other subjects, tended to be less influenced by the aspect ratio of the pots (Fig. ED14). These results are consistent with two of the factors (bottom-up saliency, and scene structure and meaning) that guide attention in visual search^33^.

Therefore, a straight-forward perceptual coupling between the experimental subjects and the material structure of their environment (ie, the pots) can explain the subject´s behaviour (in this case, their exploratory eye movements). Perception-action couplings such as this can produce complex adaptive behaviours; patterns that are stable across subjects and which, as current embodiment theories propose^3^, are capable of replacing internal cognitive algorithms and leading to stable functional behaviour without the need to resort to abstract mental representations or complex simulations.

Our analysis supports the idea that the Vi of prehistoric pottery increases with the development of complex societies and enlarged social ranking and hierarchization (Table ED1)^22,34,35^. Therefore, a negative Vi predominates in Neolithic societies (pots 1 and 2), whose social relations were simpler and negotiated at the local or community level. A higher Vi is found in the material culture of the hierarchical aristocratic societies (pots 4 and 5). In between these historical groups there was a major transition from the Neolithic peasant societies to the appearance of warriors and ranked society in European Late Prehistory whose pottery (Bell Beaker, pot 3) is associated here with a sudden change in the Vi. In particular, the perceptual footprint of the Bell Beaker confirms that the tremendous novelty of this material style embodied the new social conditions that arose during this period. This embodiment of the social in the pot would be the common under the two main but distinct processes (invention and diffusion in some areas, as well as population movements and replacement in certain other areas) to explain the extraordinary expansion of this pottery throughout vast areas of Western Eurasia^29^. This general pattern of change in the Vi over time is relevant, as firstly, there is a horizontal bias in natural images that imposes a horizontal visual exploratory behaviour^36,37^ which must be compensated for by a change in the structure of human artefacts; and, secondly, because it is easier to move the eyes horizontally, as the muscles involved are not antigravity muscles^38^.

## Conclusions

It is a well-known fact that the world around us displays consistent statistical properties that are deemed to be essential in order to understand how the human visual system analyzes images^39–43^. Nevertheless, the world is a material setting formed by natural features (affected by humans on a very small scale) and artificial (human-made) features in a proportion that depends on each type of society, and which changes over time. As the former decreases and the latter increases, societies are seen to become more complex. Therefore, our results hints at how these statistics relating to the visual world can be continually transformed and reworked by human activity in a coordinated way, together with other facets of culture and social contexts. This means it is likely that these factors evolved at the same time, producing a particular cognitive footprint in a far more complex manner than previously thought.

It therefore seems that the brain makes use of the material structure of the objects around us, using cognition to establish a direct connection, between perceptual input and behavioral and social output: after all, eye movements can be easily read and decoded by other conspecifics^11,44,45^. So they can be seen as windows into how others judge the material structure of the environment and, ultimately, the structure of society itself, becoming a fundamental aspect of human nature and social behaviour. By showing how cognition can be biased by the material properties of visual stimuli, our results show that the world and cognition are connected through a permanent feedback system of stimuli, perception, and then behaviour (as suggested by the perceptual control theory^46^), thereby connecting what and how humans see, with what humans do, and how they do it. The natural world is given to humans; it shapes their cognition that affects their behaviour. But the human world is built, therefore it is the product of a previous cognitive process what influences behaviour and then consolidates or re-shapes cognition. Due to its plasticity, the brain is clearly capable of adapting to changes in the environment, whether this is a wholly natural environment, or one that is partly or completely artificial. The difference is that, if natural, it is nature what determines visual cognition; but in the case of an artificial environment, then we as humans directly affect our own and other people’s minds, thereby creating a loop between what we see and what we think mediated by our actions. In this case, it is humans themselves who transformed their behaviour, as we can see from the varying cognitive footprint of different archaeological materialities over time. The very fact that different kinds of people from the same cultural background, reacted in our experiments to the different types of stimulation produced by the materials from societies that are far-removed in terms of time and culture, speaks for the strength of the effect of materiality on cognition. The example of Bell Beaker pottery proves that it does not matter whether Beaker people saw the Beaker pots in the same way as our present-day experimental subjects, because what is important is to check the cognitive breakup (or disruption) that they signified.

Here our experiments provided consistent and reliable results in this direction. They underpin a number of relevant conjectures about human cognition and its relationship with the material world, particularly a world moulded by humans. However, before making any generic statement about cultural evolution and the human mind, it will be necessary to extend these experiments in order to involve other kinds of materiality and other cultural contexts (archaeological and anthropological) of a comparable and distinct level of social complexity. Plans are currently underway to carry out this multifaceted research.

## Methods

Methods, along with further analytical results and additional figures are available below. The additional display items of this section (figures, photos and one table) are incorporated on Electronic Supplementary Material. References unique to this section appear only in it.

### Experiment process

Although we do not have access to a sample of representative individuals from the different cultural contexts, the stereotyped and consistent changes observed in the visual structure of the artifacts should produce a commensurable change in the exploratory behaviour of present-day individuals with a normal visual function. Therefore, one hundred and thirteen subjects participated in three different and successive experiments, completing a total of one hundred and thirty-eight trials (see below for further details about the experimental process). In Experiment 1, sixty-eight of these subjects (seven subjects were discarded for technical errors and the final valid sample comprised sixty-one subjects), visually explored photographs of five different pots, one of each style (Fig. ED8) plus one distractor. The pots were replicated for the experiment from actual archaeological items through a technological process that emulated the same formal and visual characteristics of the original pots (Supplementary Information SI2). Using replicas helps to overcome the artificial visual salience that would have been introduced by the fragmentation of reconstructed pots. In this first experiment, subjects were assigned to one of four different groups according to their degree of familiarity with the visual stimuli. Group 1 (G1, n = 13) included experts who were familiar with the specific pieces, and who had a priori knowledge of the methodology, working hypotheses and main aims of the experiment. Group 2 (G2, n = 12) was composed of trained archaeologists who knew the material but did not have any information about the experiment and/or working hypothesis. Group 3 (G3, n = 11) consisted of potters, specialists who create items as artists or artisans as a part of their daily lives. Finally, Group 4 (G4, n = 25) consisted of ‘non-experts’ from the general public, with very different educational backgrounds, and from both urban and rural environments.

Experiments 2 and 3 were used as controls and to account for any potential visual biases in our sample. The visual stimuli for Experiment 2 were 40 line drawings that included representations from the 5 pots from the first experiment, 10 other prehistoric original pots, and 5 groups different arbitrary variations of the five pots from Experiment 1 (Fig. ED9). Thirty-six subjects participated in this experiment, twenty-five of whom had previously taken part in Experiment 1. Experiment 3 was completed by 34 subjects who had not been involved in the previous experiments. This included a diverse set of 50 images and drawings comprising the pots used in Experiment 1, together with images simplifying the decoration to conventional representations (circles with vertical, diagonal and horizontal lines), and other completely different objects used as distractors (Fig. ED10). Experiments 2 and 3 investigated the particular roles that the diverse shapes, essentially height-to-width aspect ratios, and decoration patterns have in biasing the spontaneous visual behaviour.

Our results were not affected by differences in gender (Fig. ED11) or age (Fig. ED12), were similar in the different sample groups (Figs ED13, ED14), and were also consistent when using either pictures or drawings (Fig. ED15), suggesting that it is the envisioned visual saliency of the decoration that drives the trajectories of the spontaneous exploratory eye scan paths (see Fig. 1). We used 4 popular saliency models (Itti-Koch saliency model^47^, GBVS^48^, RARE^49^, and AWS^53^ to map the spatial lay out of the visual features that are likely to draw attention on the basis of evidence from studies of the visual systems of different mammalian species, including humans^54^. Our results show that the fixations and the orientation bias in the eye movements of our observers can both be predicted from these 2D saliency maps (Fig. 1, bottom line), particularly those of the drawings that isolate the contours and decoration of the pots from any unintended or noisy source of saliency resulting from the manufacturing process (Fig. ED18^52^).

#### Eye-tracking of prehistoric pottery

The apprehension of the external world with the visual part of the brain can be easily measured by eye-tracking. Visual perception is the result of a process in the brain which decodes the electromagnetic signals from the world, including eye movements, the filtering introduced by the retinal mosaics, and the specific processing performed by a hierarchy of different brain nuclei and cortical areas. Through visual behaviour we can examine the effect of material things in cognition.

This research is more oriented to explain how one gazes than where one looks, which is what a salience model feasibly. Thus the objective of our study is to predict how gaze operates, which visual gestures use and to define by what factors the visual exploration is determined.

Ceramics is a suitable object for this study because on the one hand it characterizes adequately each archaeological period, is very abundant, well studied and its formal diversity is known, and on the other it generates clear oculomotor responses that are easy to handle in an experiment of this type. Moreover, the decorations that appear in the pottery tend to appear in other mobile or non-mobile cultural items (figurines, ornaments, stone placs, bone amulets, or stone recipients, sculptures, rock art, architecture …)^53^.

#### Prehistoric pottery considered in this study

The pieces we analysed are very different, depending on the ceramic styles to which they belong. They present both variations in the shape of the ceramic container, as in the decoration that applies to it. The variety of shapes includes vases, jars, urn, casseroles and great vessels that were quite sure store pots.

In all cases selected the decoration is based on geometric motifs, as is the dominant trend in European prehistoric pottery, where the naturalist decoration and figuration is exceptional. Altogether they range from the middle (St1) and late (St2) stages of the Neolithic (the first village settlements), through early Bronze Age (St3) to the end of Protohistory (St4, St5), covering a variety of socio-cultural forms (see Table E1 below), from simple communities based on the house and the family which are relatively egalitarian (St1, St2), through to social formations based on hierarchisation (St3), to the aristocracy and ranked societies, and finally complex states or proto-states (St4 and St5).

#### Experimental process

The experimental process involved a considerable amount of effort, approximately 2,660 hours (of a team with 10 people) from May 2014 to March 2015, plus 131 volunteers who devoted a total of around 250 hours.

The volunteers taking part in the ETA were carefully recorded but with full warranties of preserving their identity. Contextual data recorded data were age, place of origin and main characteristics (place of residence, education, training, professional activity, language, social identity, etc.).

The study implied three different experiments:

**Table:** 

	**Short reference in this paper**	**Code of the Experiment**	**Link to dataset and further details**
*First Experiment*	Exp1	EXP_14061	http://hdl.handle.net/10261/153984
*Second Experiment*	Exp2	EXP_14091	http://hdl.handle.net/10261/153984
*Third Experiment*	Exp3	EXP_15011	http://hdl.handle.net/10261/153984

We recruited 113 healthy subjects with normal or corrected-to-normal vision using the Institute of Heritage Sciences (CSIC) subject pool. Subjects gave written informed consent. All methods and procedures were performed in accordance with the relevant guidelines and regulations provided by the ethics committee of the Spanish National Research Council (CSIC) and University of Santiago de Compostela (USC). The experiments were made upon the guidelines approved by the Galician Regional Committee of Research Ethics (CAEIG) in 2012 and 2015 for the Eye-tracking experiments of the lab we used. (*Comité Autonómico de Ética da Investigación de Galicia* - CAEIG; Address: Secretaría Xeral- Consellería de Sanidade, Edificio Administrativo San Lázaro s/n, 15781 SANTIAGO DE COMPOSTELA, Tel. + 34 881 546 425; e-mail: ceic@sergas.es; Website: https://acis.sergas.es/Paxinas/web.aspx?tipo=paxlct&idTax=15534&idioma=es).

These experiments involved 113 subjects in total (average age 34, age range 23–59) who carried out 138 different tests (25 people repeated Experiment 2 as a part of the experimental strategy).

A total of 68 experimental subjects took part in Exp1, 7 of whom had to be excluded from the analysis due to the recording conditions (the 2 young children, 3 people due to the recording conditions and 2 for other reasons, including one of the authors –FCB). The mean age of the remaining 61 subjects was 36, with a range of 15–58 years. For the minors of the study, written informed consent was obtained from their parents or legal guardians. Besides, all underage subjects are children of one of the authors (FCB) and have therefore parent consent. However, the data from these experiments were not proceeded because their proper analysis would has required a bigger sample population; thus these results does not make part of this paper. The subjects conformed 5 groups (different sample populations) based on their degree of familiarity with the pieces and working hypotheses: a first group (G1) consisting of 13 people who had a high degree of specific knowledge and all of the whom were aware of the working hypotheses and the aims of the project, and members of the same research institute where the research was carried on (6 men and 7 women; mean age: 40; range: 29–45); a second group (G2) of 12 people with a high degree of specific knowledge (archaeologists) but who were not familiar with the working hypotheses (7 men and 5 women; mean age: 34, range: 22–57); a third group (G3) of 11 specialists in the production of ceramic items (6 men and 5 women; mean age: 49, range: 31–58), including the three artisans who did the replicas of the pots (their results fit very well into the same trend as the others); a group (G4) consisting of 25 members of the general public with no specific familiarity of the pottery or the working hypotheses (10 men and 15 women; mean age: 30, range 22–50); and finally, a study group was put together of 3 teenage girls (15, 15 and 17), that gave interesting results but were discarded because lack of statistical significance.

A total of 36 people took part in Exp2, of whom 25 had already taken part in the previous experiments but none of them were members of the Incipit and them they were unaware of the project: 18 men and 18 women, mean age: 34, range: 23–59. Three people were excluded due to the recording conditions (33 valid trials).

Finally, 34 people took part in Exp3. Most of them were university students, including: 154 men and 19 women, mean age: 22, range: 18–27.

#### Equipment

The study was conducted at Santiago de Compostela University’s Faculty of Psychology. The testing laboratory contained two rooms, one for the experimenter monitor and the other for the participant monitors, with adjustable chairs and tables. Adjustments were made to maintain the participant’s eyes at 70 cm from the 386 × 333 mm monitor (IBM P211, 2048 × 1536 pixels, 60 Hz). Eye movements were collected using an Eyelink II (SR Research Ltd., Osgoode, Ontario, Canada) 500 Hz eye tracker.

The recording software is hosted on a dedicated computer for that task. The experiment is controlled from a second computer that also commands the presentation of the stimuli. The eye movements are recorded by two cameras of 500 Hz each; the registration system is adjusted to the head of the subject by a helmet adjustable in diameter. Moreover, a third camera reports the position of the head with respect to the stimulus presentation screen. The presentation of stimuli and coordination between the registration system and the presentation system was implemented in MATLAB based on the Psychotoolbox and the specific toolbox for Eyelink.

#### Procedure and design

Each participant completed demographic and consent forms. Subsequently, the recording chambers were adjusted and the sampling was verified to be stable at all points on the screen. Then the task was explained to them.

The experiment started with a nine-point calibration in which the subject had to fix the view in 9 points distributed on the screen and the subsequent validation, checking the robustness of the previous reference.

The experiment consisted of two parts, both with a presentation duration of 30 seconds. In the first part (FV) they simply had to explore the piece freely. In the second part (OD), the subjects listened to a voice message that asked them to estimate the age of the piece that was shown, after the observation period the subjects had to answer the question raised choosing the option they considered more adequate of the two that appeared on the screen. To do this, they had to press the left or right arrow. In order to avoid the displacement of the coordinate axis throughout the experiment, the subjects had to fix the view at a point that appeared in the centre of the screen before and after each presentation and that allows the continuous adjustment of the reference frame. This second part of the experiment has not been included in this research. The images (see selection of images) were randomly displayed to each new subject, although order was maintained between the first and second comparisons.

Finally, the subjects had to cover a survey of personal data for the statistical analysis of the population sample.

#### About Aspect Ratio

The height-width ratio varies greatly from one pot to another. Therefore, it was possible that what we were looking for (ie, to discover the main component of the orientation of the visual exploration of each pot), was initially determined by the basic form of the pot. To discriminate this effect, we start by looking for a numerical expression of the shape of each pot. We have called this “aspect ratio” (AR) and its formula is: AR = (V − H)/(H + V). In addition, since the hypothesis predicts a predominance of horizontality in some cases and of verticality in others, to verify this we calculate a verticality index (Vi) (see below) that allows us to compare the proportion of vertical and horizontal saccades in each image.

#### About the Vertical Index (Vi)

In free observation tasks the most common angles for the saccades are around the vertical and horizontal axis both of natural and fractal images^54^. This pattern is also found for the images used in this work (Fig. 1). Saccades with and angle between 45° and −45° with respect to the horizontal are considered as horizontal saccades. Similarly those saccades with angles between 45° and −45° with respect to the horizontal are considered as vertical saccades. The same angle classification was applied to the drifts, in this case considering the angle formed between the final coordinate of the previous saccade and the initial one of the next saccade. To calculate these ratios we used the following formula:

$${\rm{Vi}}=({\rm{W}}\ast {\rm{NVS}}-{\rm{H}}\ast {\rm{NHS}})/({\rm{W}}\ast {\rm{NVS}}+{\rm{H}}\ast {\rm{NVS}})$$

Where H is the screen height in pixels; W is the screen wide in pixels; NHS is the number of horizontal saccades; and NVS is the number of vertical saccades.

The normalization regarding the screen dimensions seemed convenient to ease comparison with other screens and is based in the one used by Lau and col^55^. The drifts were also analyzed in a discrete way following the same formula.

#### About Figure 1

The ellipse describes the variability of the eye fixations about its mean position by assuming that the fixations have a bivariate normal distribution^56^. The area of the ellipse was calculated as:

$${\rm{A}}=2{\rm{k}}{\rm{\pi }}{\rm{\sigma }}{\rm{h}}{\rm{\sigma }}{\rm{v}}(1-r2)1/2,$$

being A the area of a bivariate normal ellipse, σh y σv the standard deviations along the two meridians, and ρ the Pearson product-moment correlation of the horizontal and vertical eye movements. The value of k establishes the confidence limit for the ellipse. In our experiments, K = 1.14 was used, which produces an ellipse where the fixation is found 68.3% of the observation time.

#### About analysis of drifts

We have analysed the drifts in detail but do not modify the conclusions we made from saccades analysis. Drifts are correction movement of the fixation and, although they tend to be mostly horizontal movements, on the other hand they reinforce the saccade and follow the tendency of this one moving primarily in the sense of the saccades.

#### Decoding Method: Linear discriminant analysis (LDA)

Decoding performances are quantified by the relative number of hits (or correct responses) that are the average of the diagonal in the confusion matrix^57,58^. As the outcomes of the predictions of each stimulus can be regarded as a sequence of Bernoulli trials (independent trials with two possible outcomes: success and failure), the probability of successes in a sequence of trials follows the Binomial distribution. Given a probability *p* of getting a hit by chance (*p* = *1/K*, in which *K* is the number of stimuli), the probability of getting *k* hits by chance in *n* trials is given by

$$P(K)=(\begin{array}{c}n\\ k\end{array}){p}^{k}{(1-p)}^{n-k}$$

Where

$$(\begin{array}{c}n\\ k\end{array})=\frac{n!}{(n-k)!k!}$$

is the number of possible ways of getting *k* hits in *n* trials. From this it is possible to assess statistical significance and calculate a *p*-value by adding up the probabilities of getting *k* or more hits by chance:

$${\rm{p}}-{\rm{value}}={\sum }_{(j=k)}^{n}P(j)$$

To validate decoding results, some trials were used as the training set. This procedure was the “leave-one-out” in which each trial is predicted based on the distribution of all the others trials.

## Supplementary information

Video POT 1

Video POT 2

Video POT 3

Video POT 4

Video POT 5

Supplementary Figures

Supplementary Information, Execution of Experimental Pots

Supplementary Information, Visual display of the reproduction process of replicas

## Data Availability

The raw and source data are accessible in External Databases: the file with anonymized personal data of the 113 experimental subjetcs, the scripts and images of the eye-tracking experiments, and the raw data provided by eye-tracker are available in ready to use files on the open access repository of the CSIC (Spanish National Research Council), digital.csic.es, under the link http://hdl.handle.net/10261/153984.
