# Supplementary material for: Coevolution of visual behaviour, the material world and social complexity, depicted by the eye-tracking of archaeological objects in humans
Source: Sci Rep. 2019 Mar 8;9:3985. doi: 10.1038/s41598-019-39661-w (PMC6408451; doi:10.1038/s41598-019-39661-w)
Supplement: Supplementary file 6 — Supplementary Figures [file 41598_2019_39661_MOESM6_ESM.pdf]

Scientific Reports
Paper after editorial revision – SUPPLEMENTARY FIGURES

Coevolution of visual behaviour, the material world and social complexity, depicted by the eye-tracking of archaeological objects in humans

versión 2.0.0, revisión 1 25 de November de 2018 · 22:08
Submission in process: confidential

Authors:

Felipe Criado-Boado[1], Diego Alonso-Pablos[2], Manuel J. Blanco[3], Yolanda Porto[1], Anxo Rodríguez-Paz[1], Elena Cabrejas[1], Elena del Barrio-Álvarez[1], and Luis M. Martínez[2]

Affiliations

[1]Institute of Heritage Sciences (Incipit), Spanish National Research Council (CSIC), Avenida de Vigo s/nº, 15705 Santiago de Compostela, Spain.

[2]Institute of Neurosciences (IN), Spanish National Research Council (CSIC) – Universidad Miguel Hernández (UMH), Campus de San Juan, Sant Joan d'Alacant, Alicante, Spain.

[3] Laboratory of Perception, Faculty of Psychology, University of Santiago de Compostela (USC), Rúa Xosé María Suárez Núñez, s/n, Campus Vida, 15782 Santiago de Compostela, Spain.

*Correspondence to: felipe.criado-boado@incipit.csic.es and l.martinez@umh.es

Electronic Supplementary Material

Supplementary Extended Data include **three documents** and **five video movies**: *Supplementary_Figures*, that incorporates the Extended Data displays (18 figures plus one table) of the Methods section; detailed archaeological and technical information about the pots and the process of experimental manufacturing of replicas used in Experiment 1 (*Supplementary_Info1_Execution-of-experimental-Pots-Replicas*, plus a Powerpoint of the experimental manufacturing process of the pots for Experiment 1 in *Supplementary_Info2_Reproduction-process-of-Replicas*. There are also some movies SI3-7: videos with the total visual movements of 61 experimental subjects for Experiment 1 (Movie SI3 for pot 1, and so on).

Supplementary_Figures

Figures texts

Fig. ED1. The full set of studied pots in this analysis, arranged according the different ceramic styles they belong (see table ED1 and Fig. ED7).

Fig. ED2. Synthesis of ceramic Style 1, Middle Neolithic-Megalithism. The graph shows more than just the typology of the period but, following a specification developed by Isabel Cobas Fernández and Pilar Prieto Martínez[57], comprehensibly summarizes the technological-operative chain of the period by displaying the different technological and formal options that the potter could choice in each stage of the making of a ceramic form. Thus, the table allows to do easy and direct comparisons between different pottery styles. Figures S2-S6 are taking from Prieto Martínez, Cobas Fernández and Criado-Boado *(20).*

Fig. ED3. Synthesis of ceramic Style 2, Late Neolithic.

Fig. ED4. Synthesis of ceramic Style 3, Bell Beaker Pottery, Early Bronze Age.

Fig. ED5. Synthesis of ceramic Style 4, Middle Iron Age.

Fig. ED6. Synthesis of ceramic Style 5, Late Iron Age.

Fig. ED7. Map showing the archaeological sites and location of the analyzed pots.

Fig. ED8. Images presented in Experiment 1 (Exp1 –no. EXP_14061); each image was shown during 30 seconds.

Fig. ED9. Images presented in the Experiment 2 (Exp2 –no. EXP_14091); each image was shown during 15 seconds.

Fig. ED10. Images presented in the Experiment 3 (Exp3 –no. EXP_15011); each image was shown during 10 seconds.

Unfortunately, we were not able to make clear the rights to publish 25 of the 54 photographic stimuli. These photographs were obtained on free databases on internet, but we were unable to contact the copyright holder and request permission to publish the images commercially, under a CC BY open access license. However, we made sure that all photographs are free of use for research purposes. We can provide these images upon request. Here, we just give the references that will allow to identify these images (see the number in the ED10 mosaic): 1- Galatea of the Spheres, painting by Salvador Dalí (1952). 2- Number 31, by Jackson Pollock (1950). 3- The Starry Night, by Vincent van Gogh (1889). 4- Las Meninas, by Diego Velázquez (1656). 5- La belle ferronière, by Leonardo da Vinci (1490-95). 6- Lady with an Ermine, by Leonardo da Vinci (1490). 7- The Gioconda, by Leonardo da Vinci (1503-19). 8- Bust of white young woman, about 20 years old, short light brown hair, Caucasian features with clear skin and pink cheeks and lips, neutral facial expression, wearing small earrings. 9- Bust of white man, about 35 years old, short dark brown hair, light skin, thick eyebrows, neutral facial expression, and wearing a red turtleneck sweater. 10- Bust of white man, about 40 years old, with pretty baldness and no shave, neutral facial expression, wearing a V-neck sweater and a brown beaded necklace. 11- Bust of white young woman, about 35 years old, medium length blonde hair, pink skin, neutral facial expression, wearing a white turtleneck sweater. 12- Outdoor architecture, specifically, a view of about fifteen buildings and skyscrapers on the shore. In this picture dominates the blue and grey colours. 13- Outdoor architecture, in particular, a brown skyscraper make up by different vertical modules, with vegetation around its base.14- Outdoor architecture, specifically, elevated view of a central point of a city completely taken up by large buildings and skyscrapers. 15- Outdoor architecture, specifically, detail view of six stories of a large building full of rectangular windows. 16- Salvator Mundi, by Leonardo da Vinci (1500). 17-

Natural, in particular, a pond of water lilies. 18- Natural, specifically, a spider web held between herbs. 19- Outdoor architecture, in particular, old door with reliefs on a stone wall with round arch. 20- Outdoor architecture, specifically, carved wood old door with round arch. 21- Outdoor architecture, specifically, old round gothic window in a stone wall. 22- Famous journalistic photograph of social violence, in particular the widely known execution of a Vietcong prisoner during the Vietnam war, taken by Eddie Adams and Pulitzer winner. 23- Photograph of social violence, specifically, a recruit of violent Islamic groups killing a young man by cutting his throat with a hunting knife. 24- Photograph of social violence in which a man in a camouflage suit smiles stretching a rope that hangs a dog. 25- Photograph of social violence in which a man attacks a woman pressing her neck, while she tries to take him away.

Fig. ED11. To complement Fig. 2, we analysed Vi responses by gender in Experiment 1. We found no significant differences between the Vi values of males and females for any of the stimuli used. The graphics show the variation of the Vi values of males and females for the different pots, the first graphic shows the average Vi index, the second graph shows a normalized average of the Vi index ranking of the different measures taken for both males and females for each pot. For a 1 degree of freedom and α value of 0.05 we should expect a $\chi 2$ value over 3.841 which is not reached for any of the pots displayed. For the pot 1 $\chi 2(1, N = 60) = 0.204$, $p = 0.652$; pot 2 $\chi 2(1, N = 60) = 2.673$, $p = 0.102$; pot 3 $\chi 2(1, N = 60) = 0.603$, $p = 0.437$; pot 4 $\chi 2(1, N = 60) = 0.11$, $p = 0.739$; pot 5 $\chi 2(1, N = 60) = 1.832$, $p = 0.176$.

Fig. ED12. To complement Fig. 2, we analysed Vi responses by age groups in Experiment 1. We found no significant differences between the Vi values of the different age groups. We found no significant differences between the Vi values of the 3 age groups established for any of the stimuli used. The graphics show the variation of the Vi values the 3 groups of age (between 20 and 31, between 30 and 41 and over 40) for the different pots, the first graphic shows the average Vi index, the second graphs shows a normalized average of the Vi index ranking of the different measures taken for the different age groups in each pot. For a 2 degree of freedom and α value of 0.05 we should expect a $\chi 2$ value over 5.991 which is not reached for any of the pot images used as stimuli. For the pot 1 $\chi 2(2, N = 60) = 3.722$, $p = 0.155$; pot 2 $\chi 2(2, N = 60) = 5.254$, $p = 0.072$; pot 3 $\chi 2(2, N = 60) = 0.988$, $p = 0.610$; pot 4 $\chi 2(2, N = 60) = 2.281$, $p = 0.304$; pot 5 $\chi 2(2, N = 60) = 2.953$, $p = 0.228$.

Fig. ED13. To complement Fig. 2, we analysed Vi responses by samples groups of Experiment 1 (see also Fig. ED17). This figure shows very clearly that the behaviour of the different sample groups is the same; the only slight difference is that the three 'expert' groups further reinforce the trend of the Vi while the 'non-expert' group that has a flatter response. This undoubtedly shows the greater attention that experts put in the decoration while the non-experts are more focused on shape, so their ocular response is largely influenced by the pot's AR. In other words, it seems clear from these results that the mark of the expert is his ability to discard much visual information that is very salient to the non-expert.

We found some differences among the different expertise classes for the images of the pots 2 and 3. For a 3 degrees of freedom and α value of 0.05 we should expect a $\chi 2$ value over 7.815 which is only reached for pots 2 and 3. For the pot 1 $\chi 2(3, N = 60) = 3.965$, $p = 0.265$; pot 2 $\chi 2(3, N = 60) = 11.239$, $p = 0.011$; pot 3 $\chi 2(3, N = 60) = 9.789$, $p = 0.020$; pot 4 $\chi 2(3, N = 60) = 3.240$, $p = 0.356$; pot 5 $\chi 2(3, N = 60) = 3.876$, $p = 0.275$. When analysing in detail the results of the two pots where the significant differences were found we see that for the pot 2, we have significant differences between the groups 1 (Incipit) and 2 (Archaeologists) $\chi 2(1, N = 25) = 4.503$, $p = 0.034$; 1 and 4 (non-expert) $\chi 2(1, N = 37) = 8.294$, $p = 0.004$ and 3 (ceramists) and 4 $\chi 2(1, N = 35) = 4.623$, $p = 0.032$. For the pot 3 we have significant differences between the groups 2 and 3 $\chi 2(1, N = 23) = 4.253$, $p = 0.039$; as well as between the groups 2 and 4 $\chi 2(1, N = 36) = 7.390$, $p = 0.007$.

Fig. ED14. To complement Fig. 2 and Fig. ED13, we compared as a whole the groups of experts (G1, G2 and G3) with no-experts (general public) of Experiment 1. We found significant differences between experts and non-experts for the images of the pots 2 and 3 in experiment 1. The graphics show the variation of the Vi values of experts and non-experts for the different pots, the first graphic shows the average Vi index, the second graphs shows a normalized average of the Vi index ranking of the different measures taken for both experts and nonexperts for each pot. For a 1 degree of freedom and α value of 0.05 we should expect a $\chi 2$ value over 3.841 which is only reached for pots 2 and 3. For the pot 1 $\chi 2(1, N = 60) = 0.295$, $p = 0.587$; pot 2 $\chi 2(1, N = 60) = 6.467$, $p = 0.011$; pot 3 $\chi 2(1, N = 60) = 5.507$, $p = 0.019$; pot 4 $\chi 2(1, N = 60) = 1.230$, $p = 0.267$; pot 5 $\chi 2(1, N = 60) = 2.463$, $p = 0.117$.

Fig. ED15. A relevant consequence of the experiments was to check that there are no significant differences in the Vi between pictures (Experiment 1) and drawings (Experiment 2). The drawings present only grayscale variations that represent the contour of the pot and the shape of decoration, while the photos show appreciable differences in coloration, texture or brightness in different parts of the same pot (see figure E16). After confirming that the behaviour of Vi is the same in photographs and drawings, from these results we worked only with drawings in Exp2, as this facilitated the work by simplifying the images.

Fig. ED16. High resolutions images of each pot in Exp1. These photos clearly show the pot´s natural salience.

Fig. ED17. (A) Vi interimage correlation average for Experiments 1, 2 and 3. (B) Comparison between the Vi interimage correlations obtained in Experiments 1 and 3 when considering the original pictures* only. (C) Comparison of the interimage correlation average of several parameters (Vi, eye fixations and saccade amplitudes) for the images presented in Experiment 3. (D) Vi interimage correlation for the images presented in Experiment 2. (E) Vi interimage correlation for the images presented in Experiment 3. (F) Interimage correlations of the different images presented in Experiment 3 for Vi, eye fixations and saccade amplitudes.

Fig. ED18. Analysis of saliency in Experiments 1 and 2. ***Panel A***: AUC (area under ROC curve) values for each image according to four saliency models: IK (colour blue), GBVS (colour green), RARE (colour orange), and AWS (colour red); to the left, values for the forty images (drawings) of Experiment 2; to the right, corresponding values for the five images (photos) of Experiment 1. We did not find consistent differences in the predictive value of the four models. Dashed line indicates inter-observer AUC, that is, the agreement between observers in their fixation maps. For each image, the inter-observer AUC provides an upper bound for the four models. ***Panel B***: AUC values for each series of drawings of Experiment 2 according to GBVS model (black line), compared with inter-observer AUC (yellow line). ***Panel C***: AUC values for each image of Experiment 1 according to GBVS model (black line), compared with inter-observer AUC (yellow line). ***Panel D***: AUC values relative to inter-observer values for images of Experiment 1 (red line) and the drawings of these same images in Experiment 2 (blue line). The AUC computation was made following the “shuffled” procedure suggested by Borji et al[58]. Comments on these data: A consequence of this analysis is that visual exploration is not only commanded by the AR or by the visual information density. In spite of the latter having an important effect on visual behaviour (since fixations are partially predicted by the IK model), these data show that different sort of salience have got different impact on visual behaviour. This aims toward some sort of cognitive salience, ie. a process that allows paying more attention to those part with more relevant information.

Figures

Fig. ED1

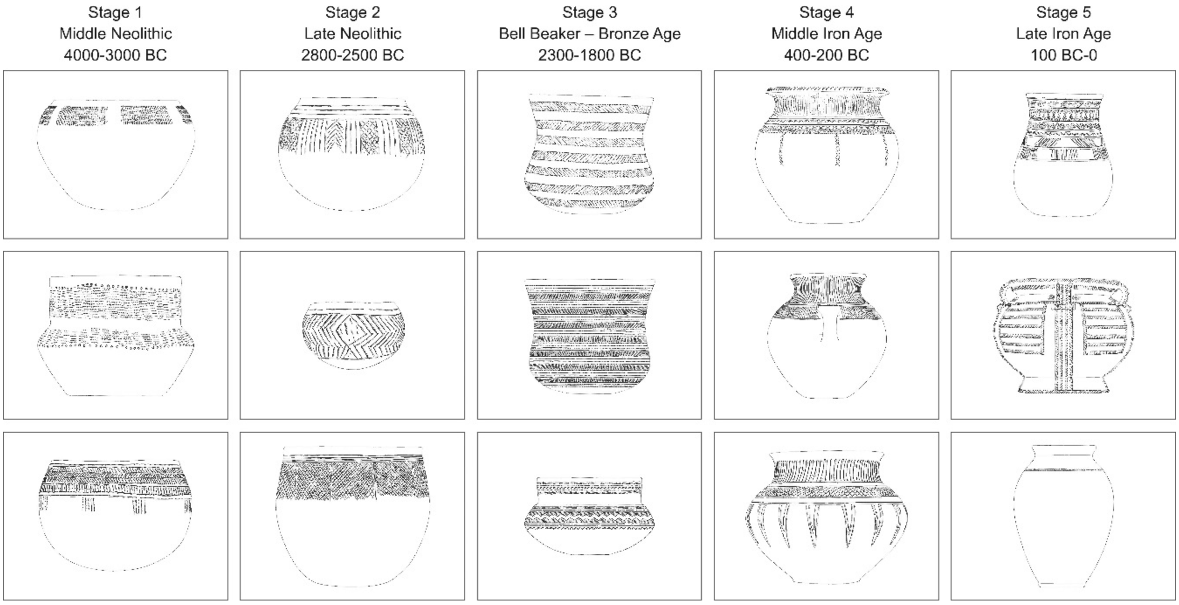
Stage 1
Middle Neolithic
4000-3000 BC
Stage 2
Late Neolithic
2800-2500 BC
Stage 3
Bell Beaker – Bronze Age
2300-1800 BC
Stage 4
Middle Iron Age
400-200 BC
Stage 5
Late Iron Age
100 BC-0

Fig. ED2

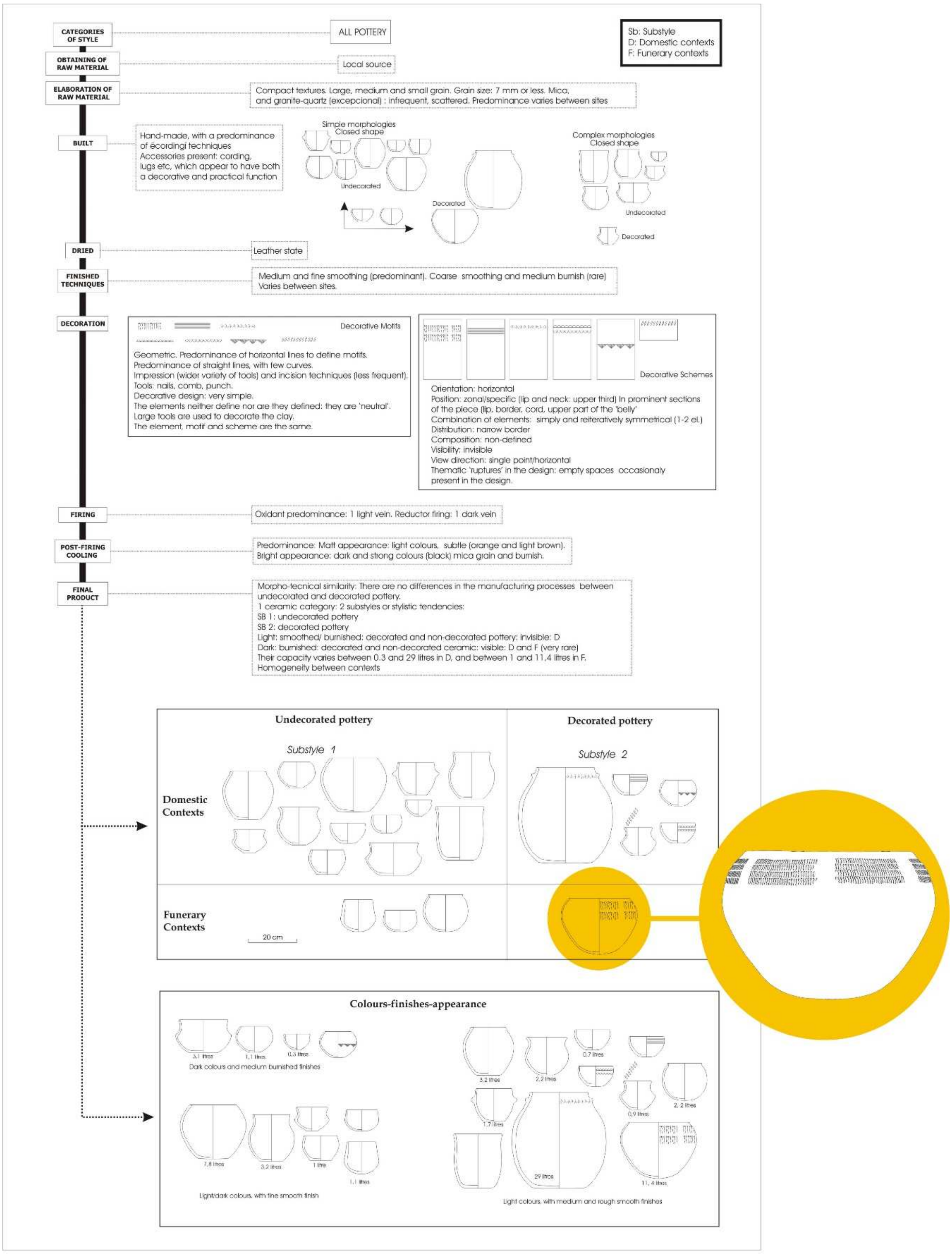

Formal characterisation of Early/Mid Neolithic pottery in Galicia (NW Spain)

Fig. ED3

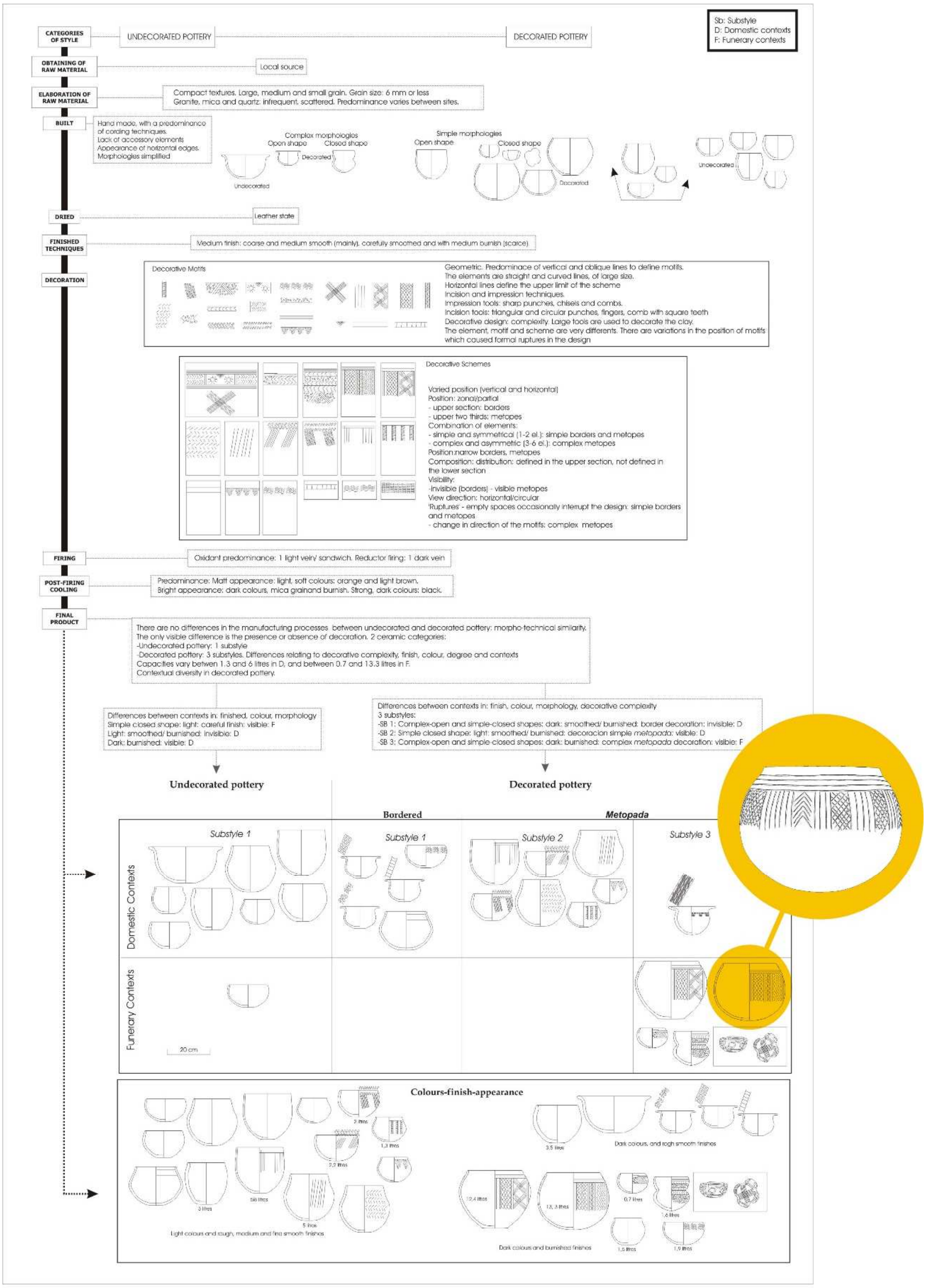

Formal characterisation of Galician Late Neolithic pottery

Fig. ED4

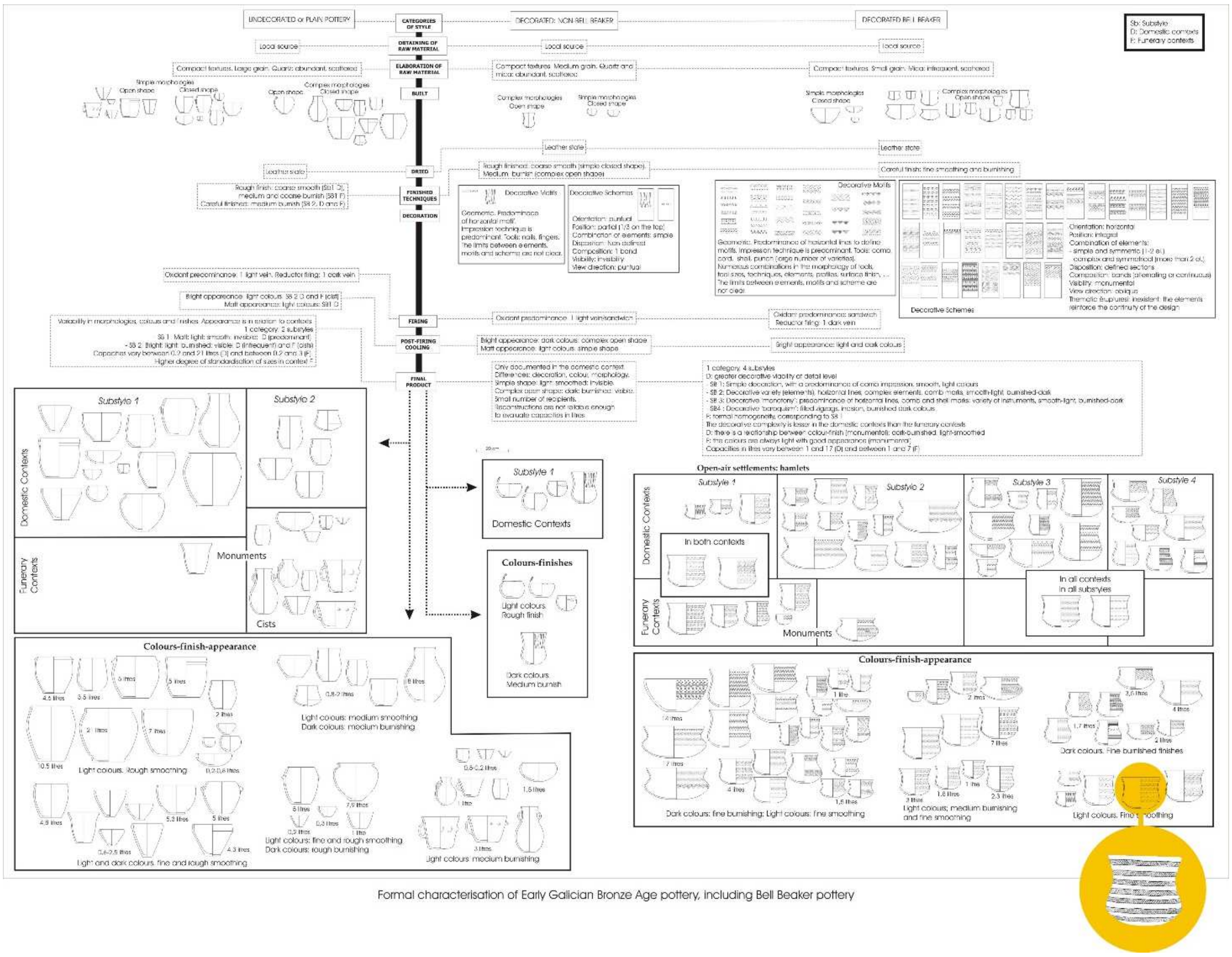

Formal characterisation of Early Galician Bronze Age pottery, including Bell Beaker pottery

Fig. ED5

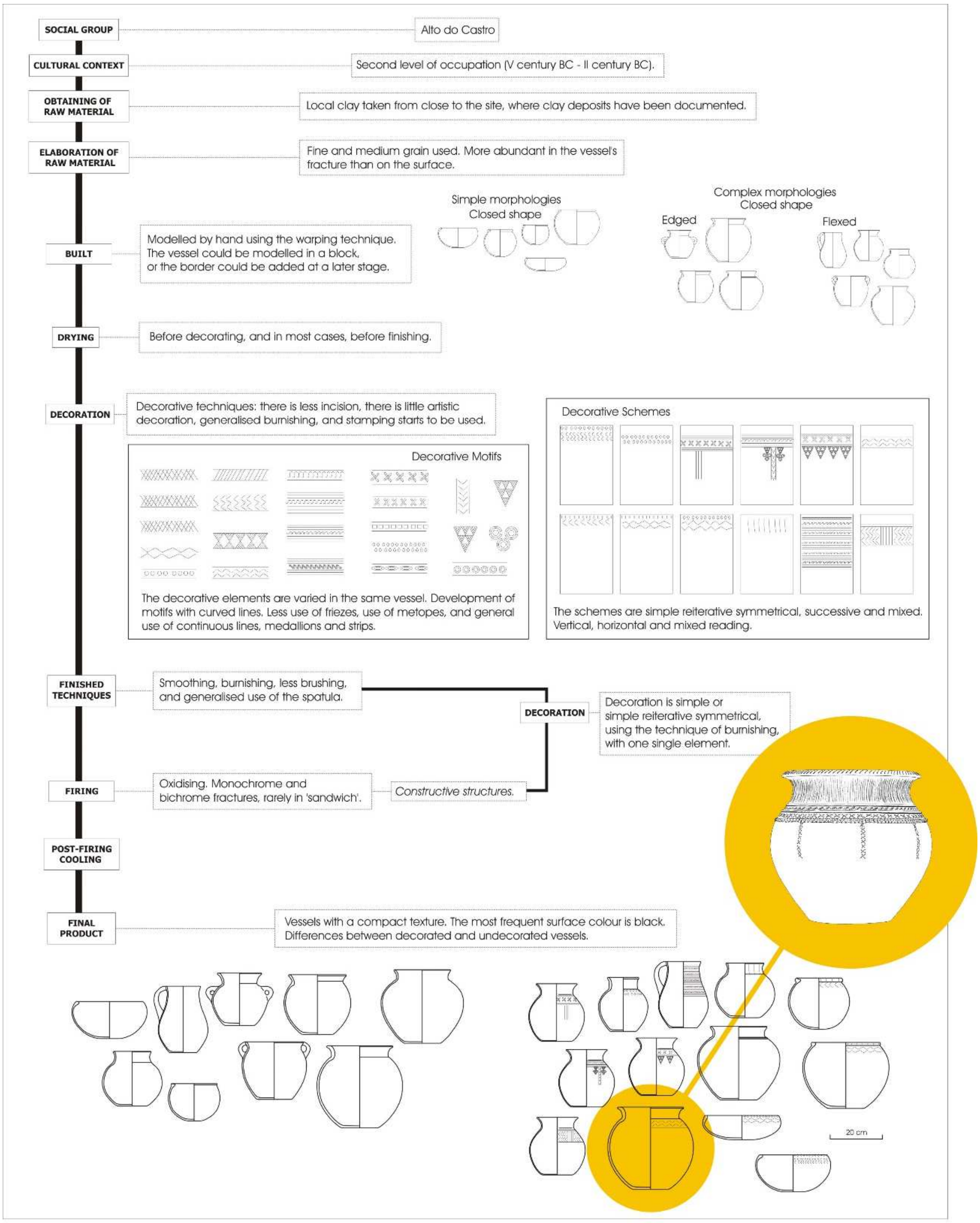

Formal characterisation of ceramics from the Iron Age II in Galicia

Fig. ED6

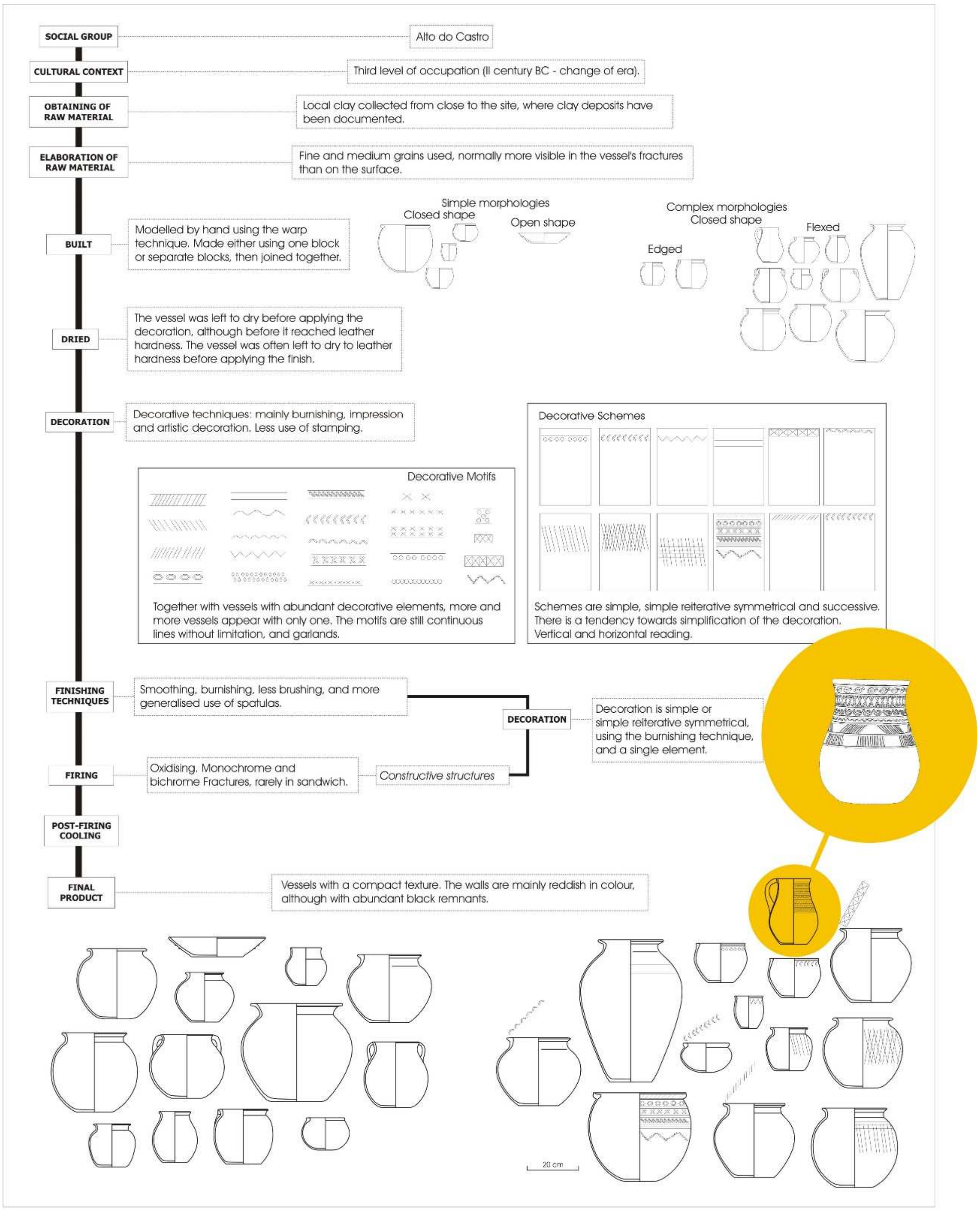

Formal characterisation of ceramics from the Late Iron Age in Galicia

Fig. ED7

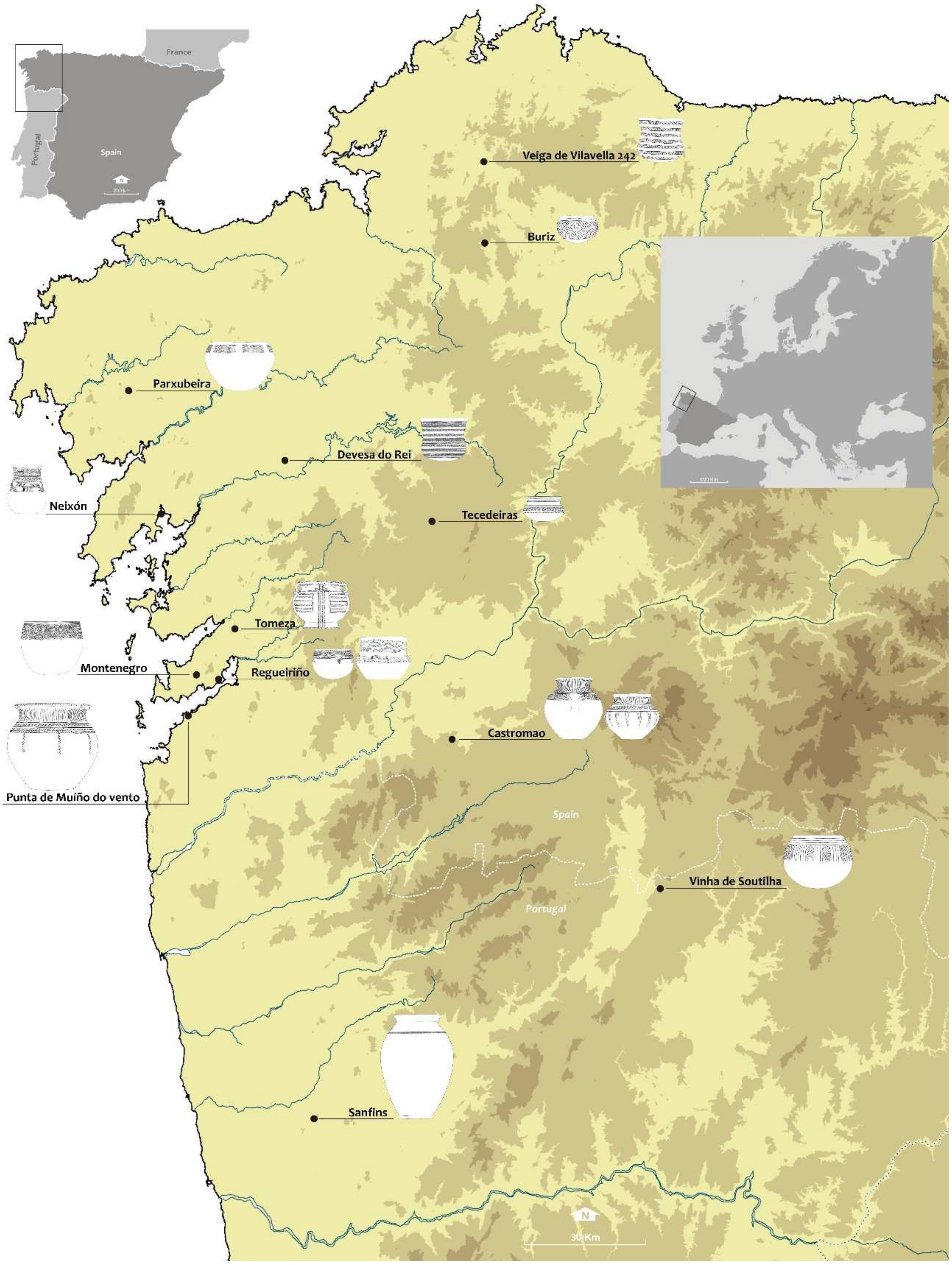
France
Portugal
Spain
Veiga de Vilavella 242
Buriz
Parxubeira
Devesa do Rei
Neixón
Tecedeiras
Tomeza
Montenegro
Regueiriño
Castromao
Punta de Muíño do vento
Spain
Portugal
Vinha de Soutilha
Sanfins
N
30 Km

Fig. ED8

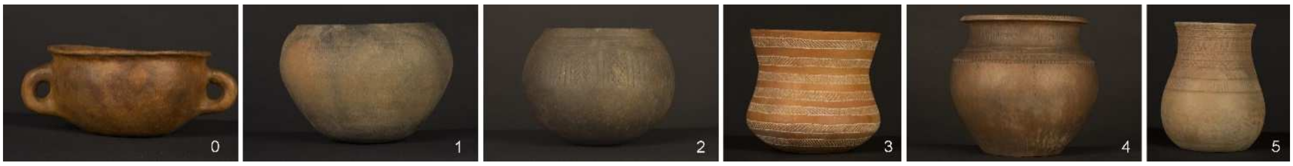

Fig. ED9

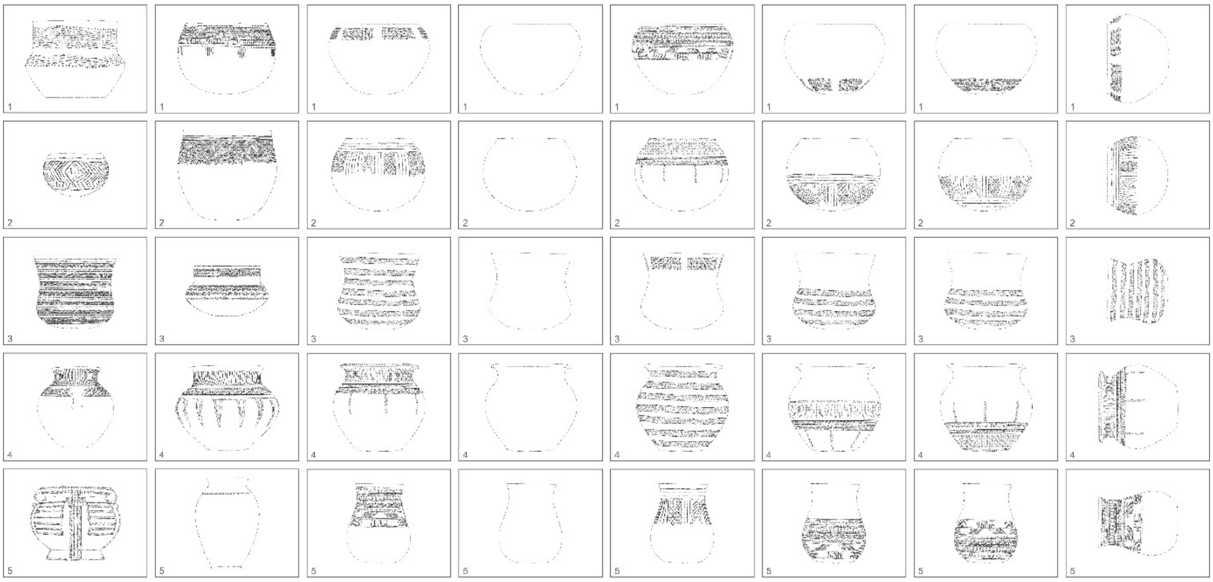

Fig. ED10

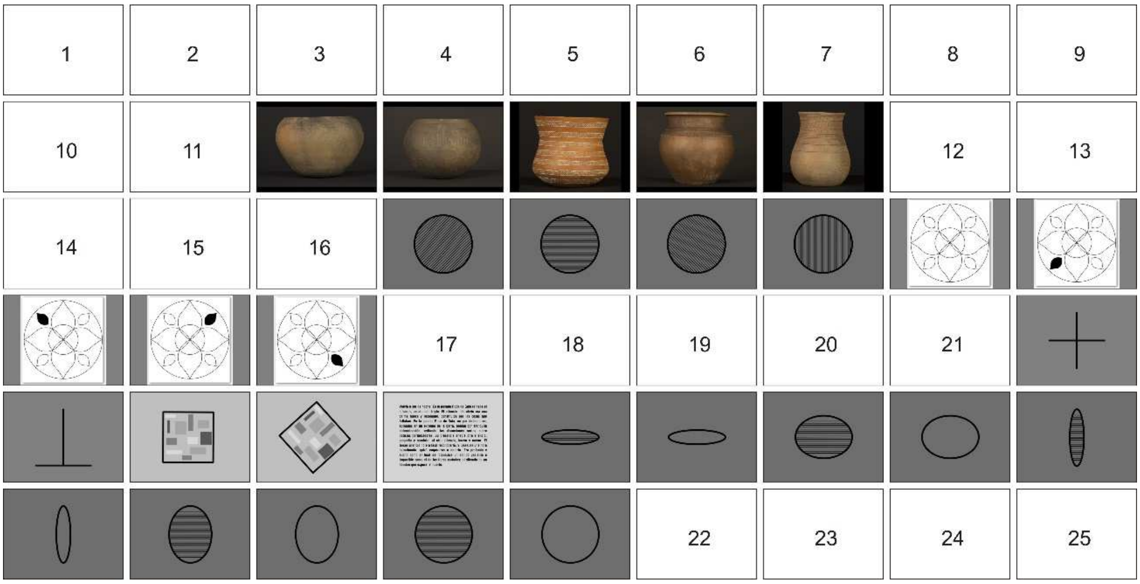

Fig. ED11

S11

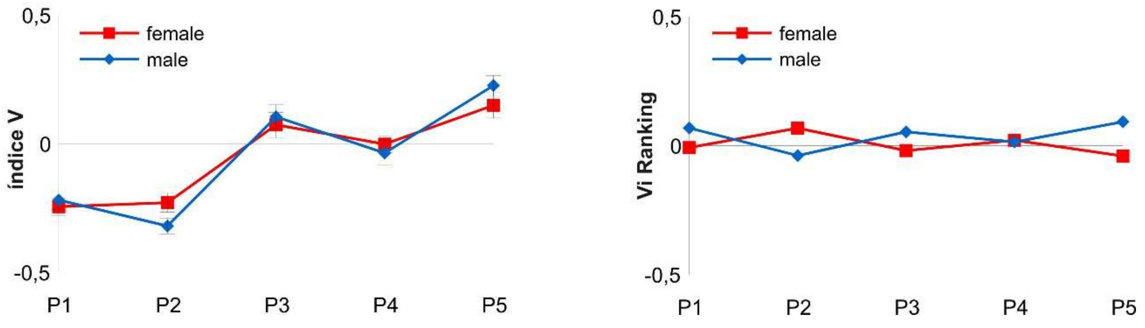

Fig. ED12

S12

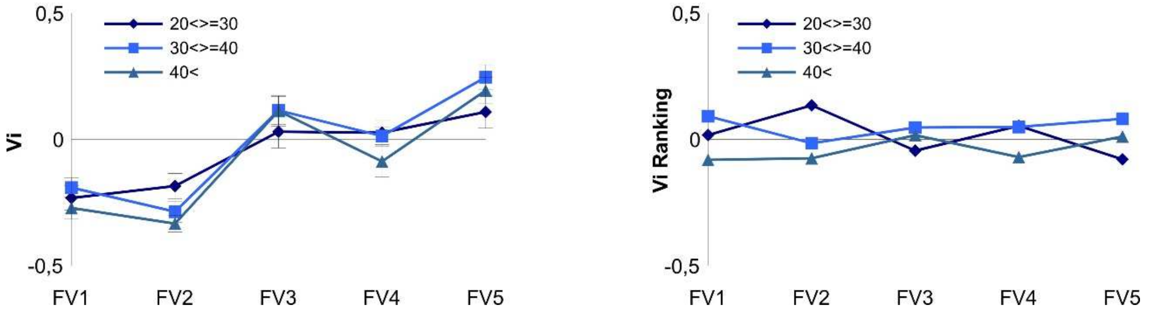

Fig. ED13

S13

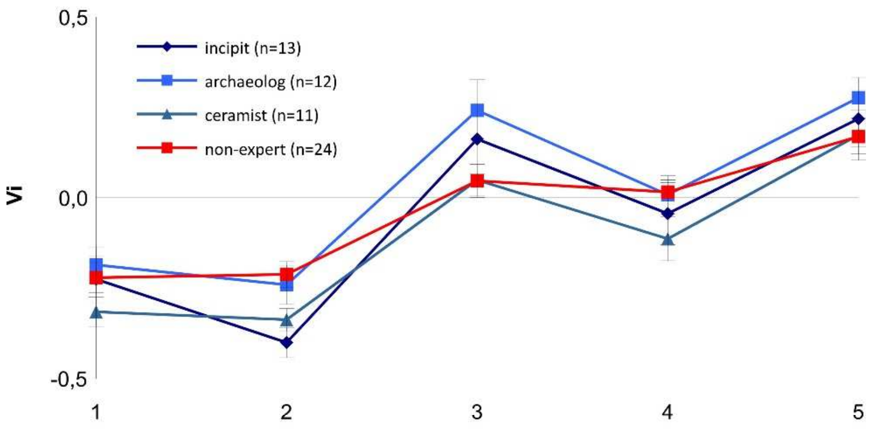

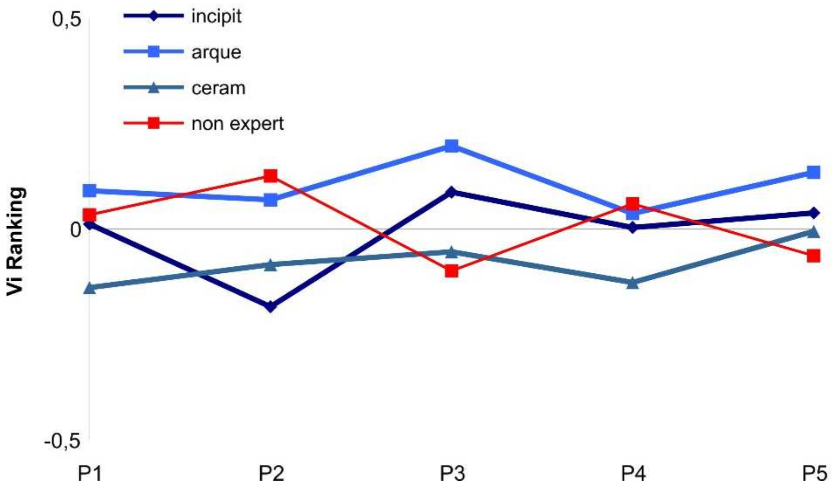

Fig. ED14

S14

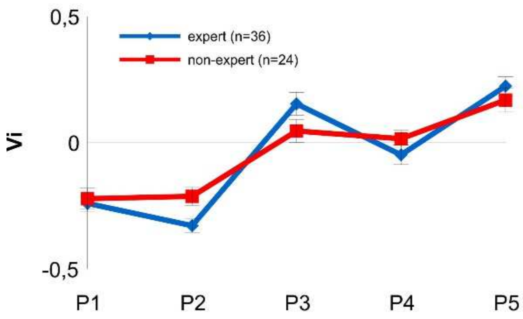

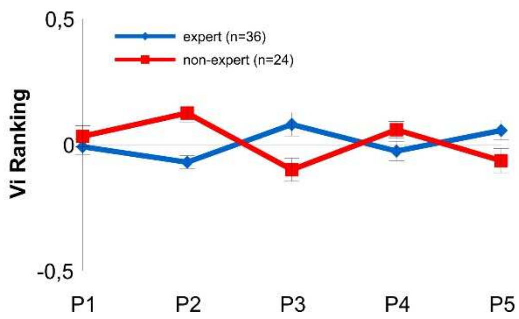

Fig. ED15

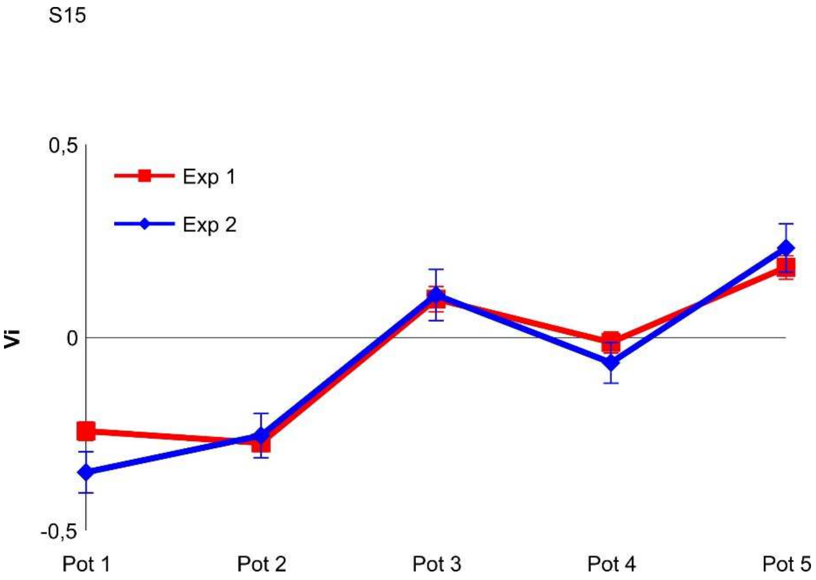

Fig. ED16-0

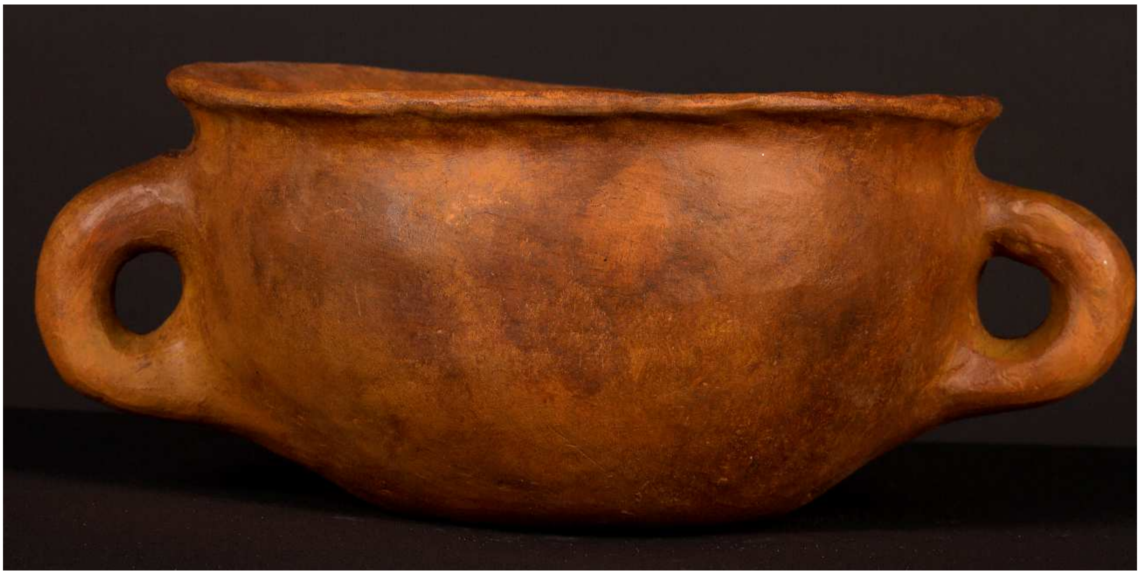

Fig. ED16-1

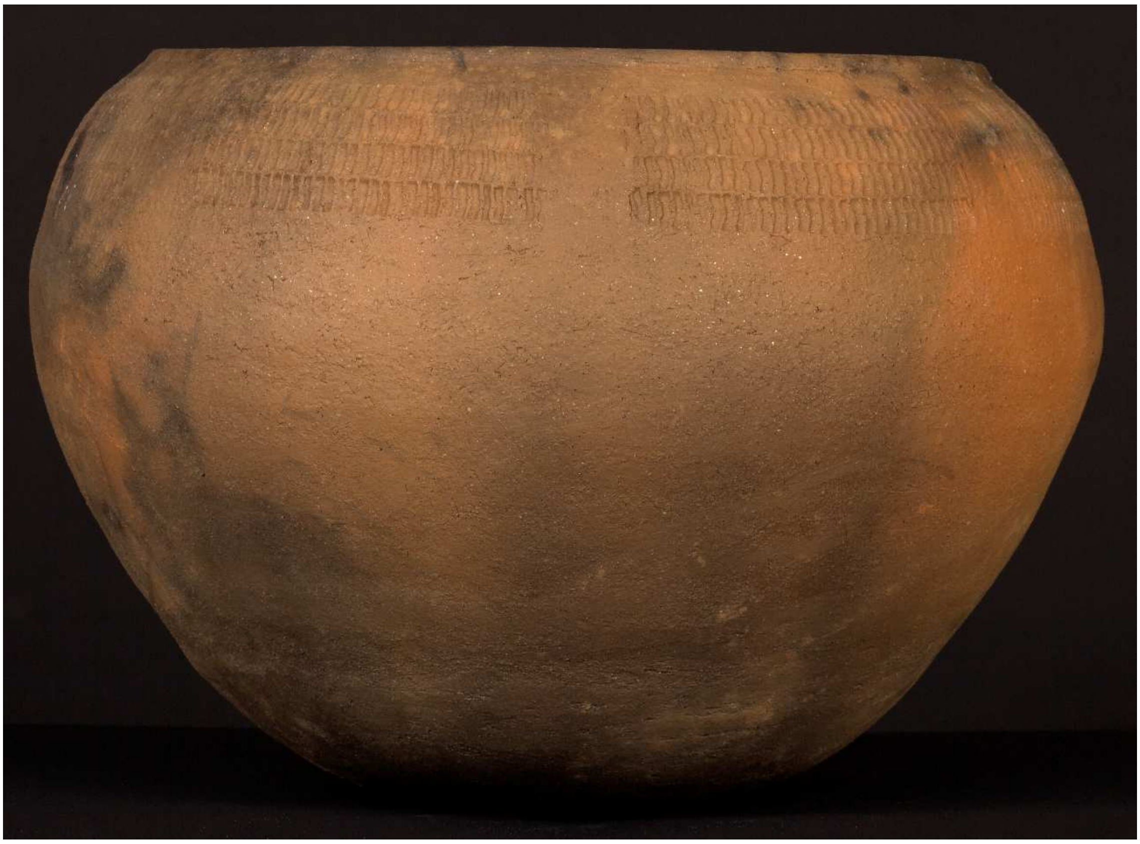

Fig. ED16-2

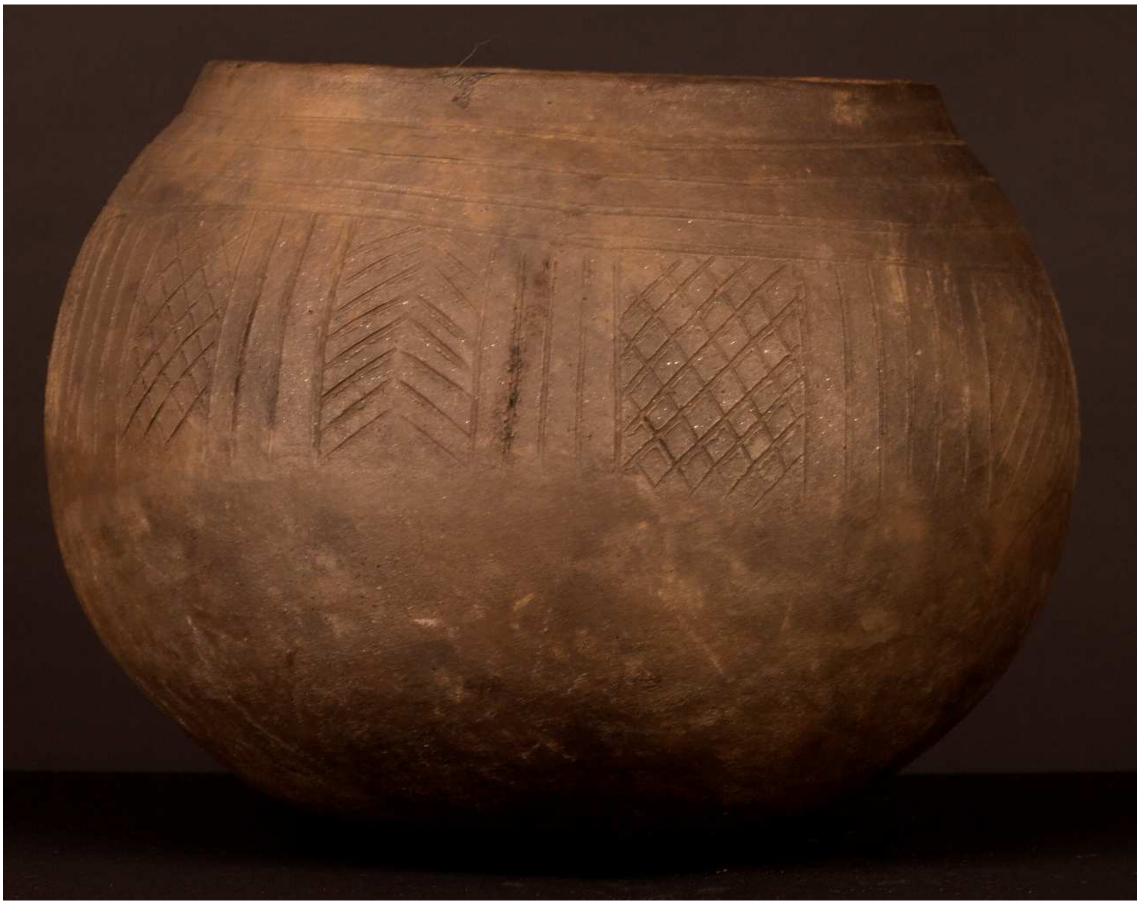

Fig. ED16-3

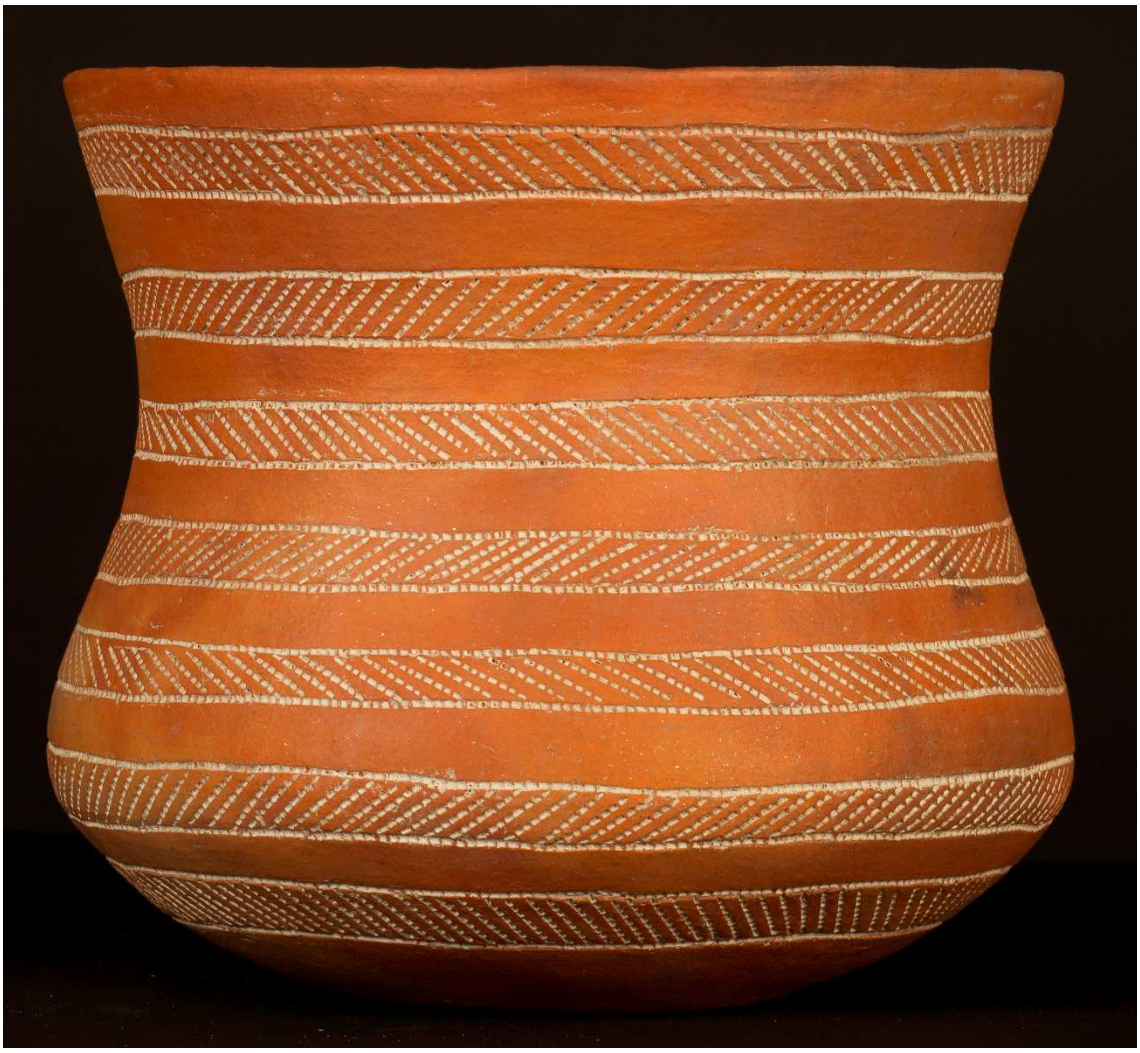

Fig. ED16-4

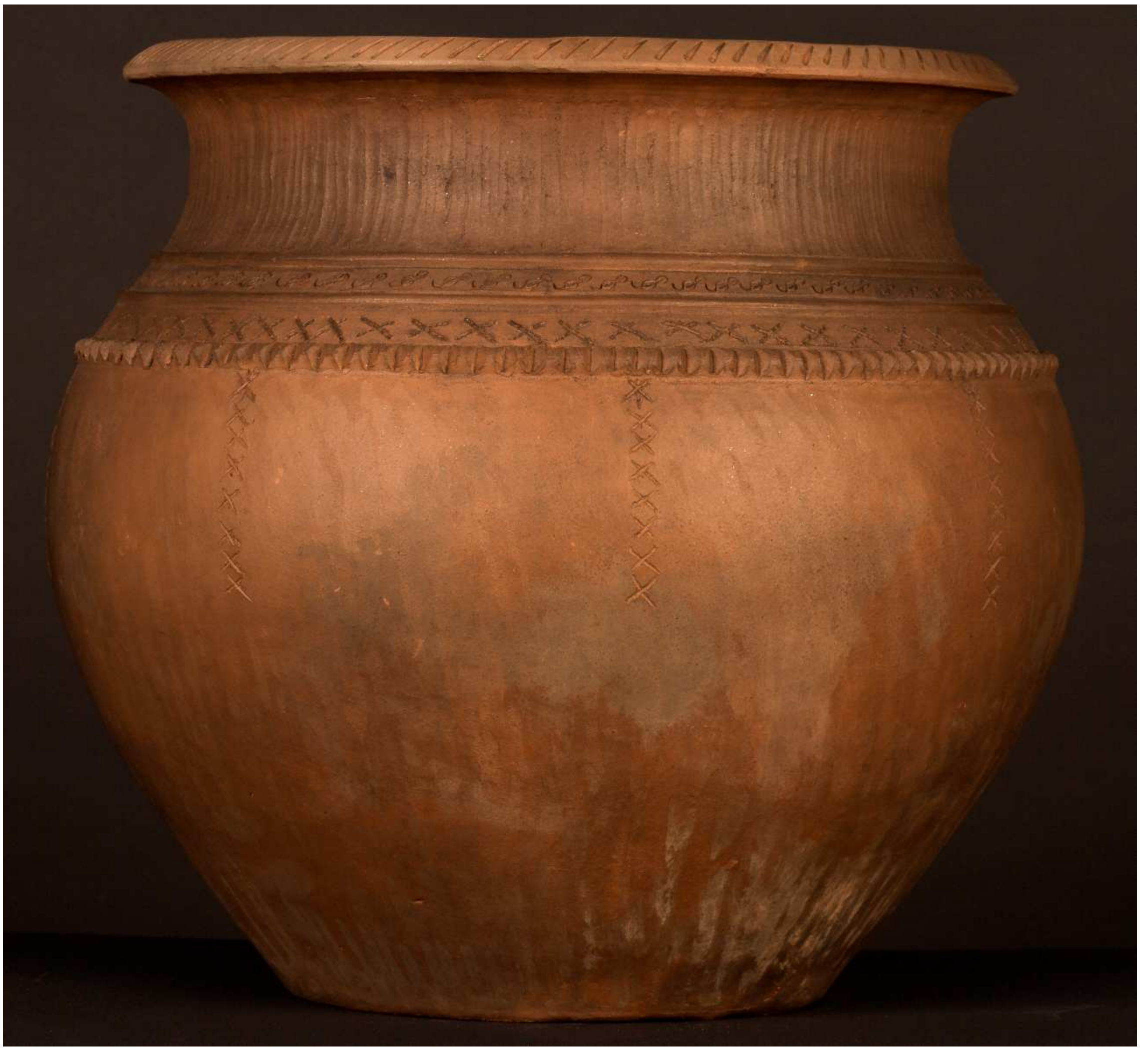

Fig. ED16-5

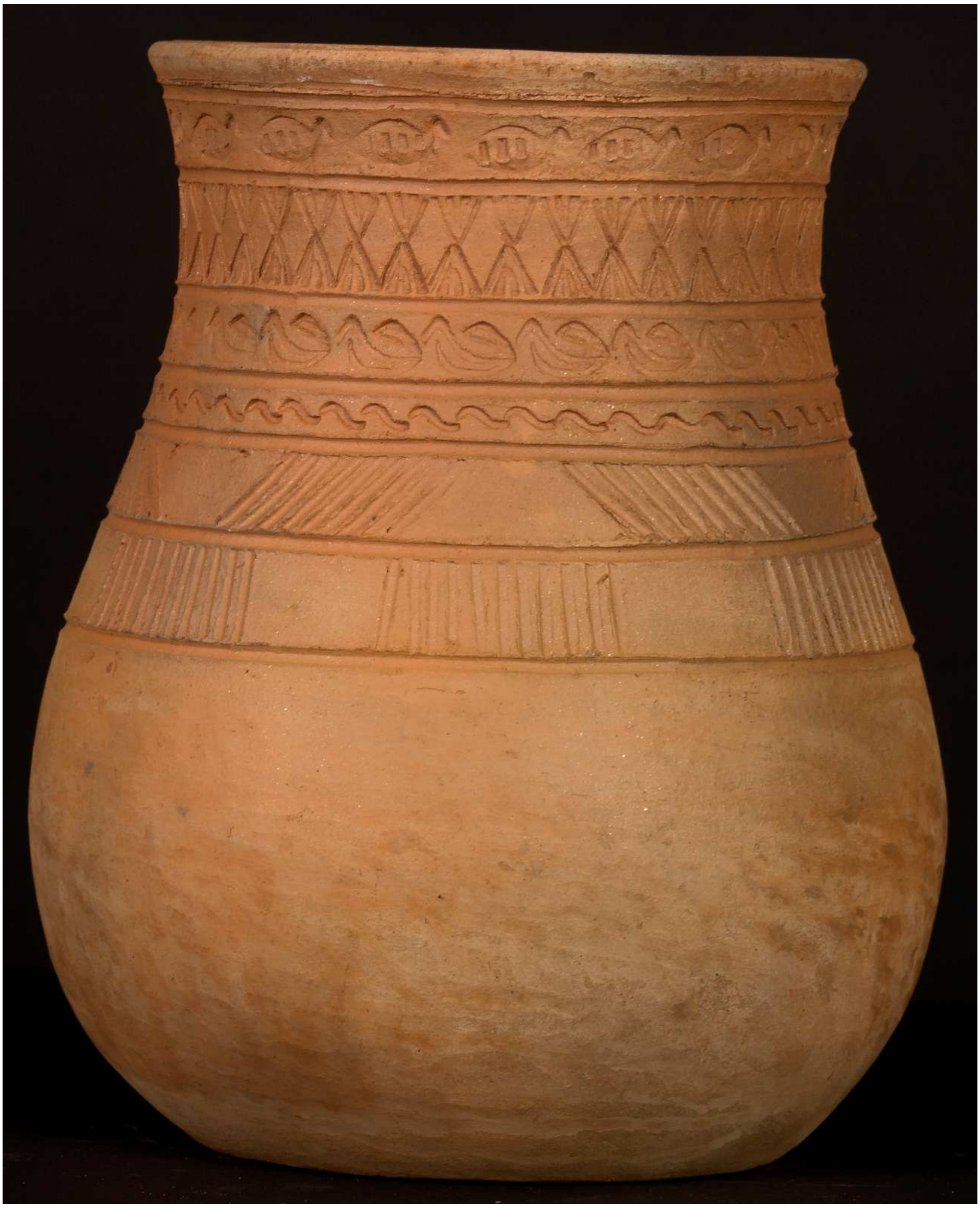

Fig. ED17

S17

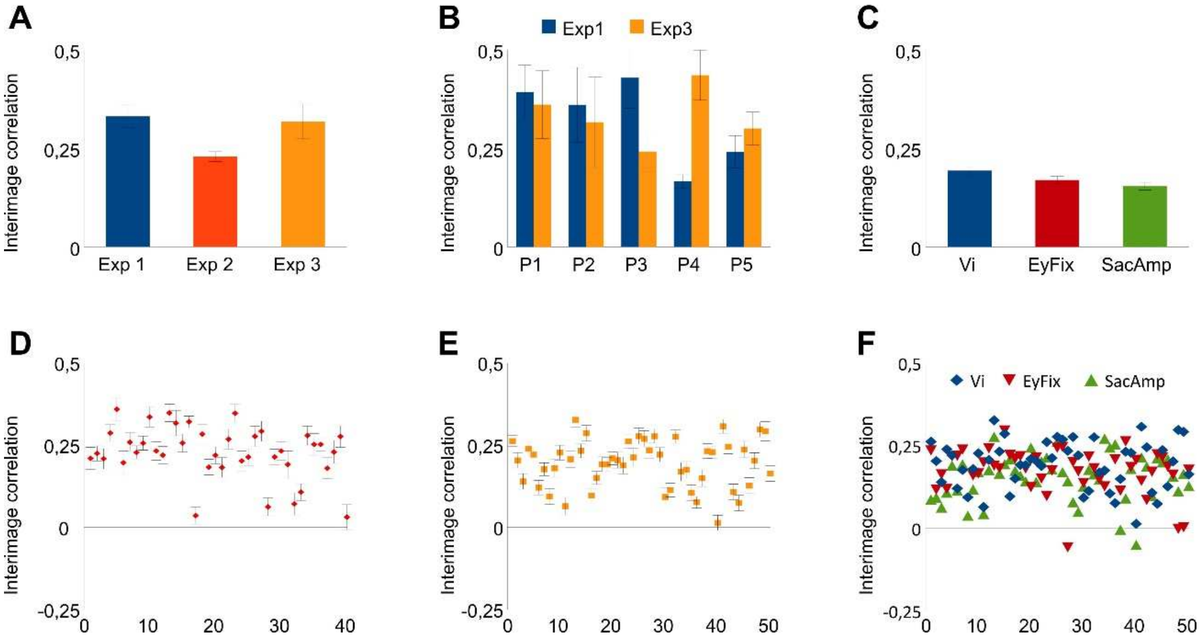

Fig. ED18

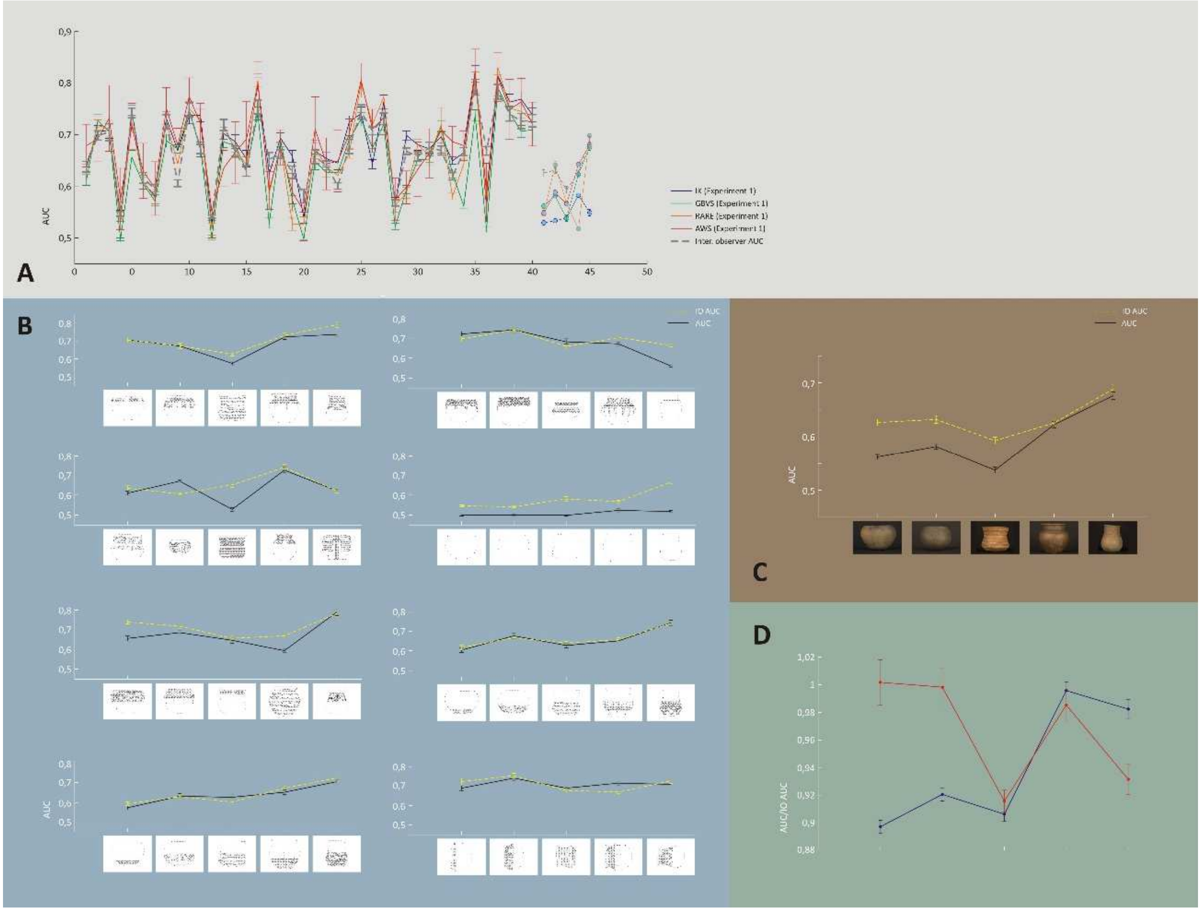

Extended data table

Table ED1.

Chrono-cultural scheme showing the chronology and main archaeological and social characteristics of the five ceramic styles analyzed. It also indicates the correlation of each of them with other archaeological phenomena and environmental events that accompany them and are common in the prehistory of Western Europe. For a more detailed account, see Parcero-Oubiña and Criado-Boado[59].

		Settlements			Monumentality		Burial practices		Production				Environmental impac	
	Ca. time period (cal BC)	Location	Form	Mobility	Where	Degree	Form	Type	Main productive activities	Storage capacity	Metallurgy	Long distance trade	Degree	Trend
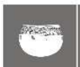	Before 4500	Uplands	Camps	Constant	Absent	Null	Unknown	Unknown	Hunting Gathering Horticulture	Null	Absent	Null	Null	-
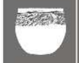	4500 – 2700	Uplands and lowlands	Camps Small open villages	Important (Seasonal?)	Burials	Big to extreme	Megalithic mounds	Collective	Slash and burn agriculture	Very low	Absent	Increase	Low to moderate (deforestation, soil erosion)	Increases
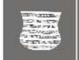	2700 – 2300	Mainly lowlands	Big enclosed villages	Low	Ceremonial	Medium	Varied (mounds, cists, pits)	Individual	Fallow agriculture	Big	Emerging	Increase	Low to moderate	Increases
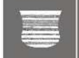	2300 – 1200	Uplands and lowlands	Small open villages	Important (seasonal)	Burials	Very low	Varied (mounds, cists, pits)	Individual	Stockbreeding Fallow agriculture	Low	Modest and stable	Decrease	Strong (+ soil acidification)	Decreases
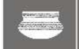	1200 – 800	Lowlands	"Pit fields"	Low	Absent	Null	Varied	Individual	Fallow agriculture	Very big	Intense (x10) Specialized Large axe hoards (often as-cast)	Increase	Very strong (+metal pollution) Peak c. 1000 BC	Increases
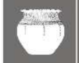	800 – 500	Upper limit of lowlands	Small hillforts	Null	Settlement	Big (widely based on natural features)	Unknown	Unknown	Fallow agriculture	Low	Modest Domestic Large axe hoards (often as-cast)	Decrease	Strong	Holds
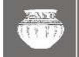	500 – 200	Lowlands	Small to medium hillforts	Null	Settlement and household	Big	Unknown	Unknown	Intensive agriculture	Big	Intense Specialized	Increase	Strong	Increases
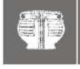	200 – 0	Lowlands	Small hillforts and Oppida	Null	Settlement and household	Extreme	Unknown	Unknown	Intensive agriculture	Very big	Very intense Specialized Gold jewelry of high quality	Increase	Very strong (+metal pollution)	Increases
