# Supplementary material for: Coevolution of visual behaviour, the material world and social complexity, depicted by the eye-tracking of archaeological objects in humans
Source: Sci Rep. 2019 Mar 8;9:3985. doi: 10.1038/s41598-019-39661-w (PMC6408451; doi:10.1038/s41598-019-39661-w)
Supplement: Supplementary file 7 — Supplementary Information, Execution of Experimental Pots [file 41598_2019_39661_MOESM7_ESM.pdf]

Scientific Reports
Paper after editorial revision – SUPPLEMENTARY INFO1: EXECUTION OF EXPERIMENTAL POTS REPLICAS

Coevolution of visual behaviour, the material world and social complexity, depicted by the eye-tracking of archaeological objects in humans

versión 2.0.0, revisión 1 25 de November de 2018 · 22:12
Submission in process: confidential

Authors:

Felipe Criado-Boado[1], Diego Alonso-Pablos[2], Manuel J. Blanco[3], Yolanda Porto[1], Anxo Rodríguez-Paz[1], Elena Cabrejas[1], Elena del Barrio-Álvarez[1], and Luis M. Martínez[2]

Affiliations

[1]Institute of Heritage Sciences (Incipit), Spanish National Research Council (CSIC), Avenida de Vigo s/nº, 15705 Santiago de Compostela, Spain.

[2]Institute of Neurosciences (IN), Spanish National Research Council (CSIC) – Universidad Miguel Hernández (UMH), Campus de San Juan, Sant Joan d'Alacant, Alicante, Spain.

[3] Laboratory of Perception, Faculty of Psychology, University of Santiago de Compostela (USC), Rúa Xosé María Suárez Núñez, s/n, Campus Vida, 15782 Santiago de Compostela, Spain.

*Correspondence to: felipe.criado-boado@incipit.csic.es and l.martinez@umh.es

Electronic Supplementary Material

Supplementary Extended Data include **three documents** and **five video movies**: *Supplementary_Figures*, that incorporates the Extended Data displays (18 figures plus one table) of the Methods section; detailed archaeological and technical information about the pots and the process of experimental manufacturing of replicas used in Experiment 1 (*Supplementary_Info1_Execution-of-experimental-Pots-Replicas*, plus a Powerpoint of the experimental manufacturing process of the pots for Experiment 1 in *Supplementary_Info2_Reproduction-process-of-Replicas*. There are also some movies SI3-7: videos with the total visual movements of 61 experimental subjects for Experiment 1 (Movie SI3 for pot 1, and so on).

Supplementary_Info1_Execution-of-Experimental-Pots-Replicas

Introduction

A total of 5 ceramic pieces are selected from archaeological origin of different contexts and eras. Two of these pieces are completely preserved (the containers type *penha* –nº 2- and the bell-beaker vessel–nº 3), so their shape, type of decoration and distribution are known. In the other three cases some fragments are preserved, which, taking into account what is known from other objects of the same period and style, allow to make a hypothetical reconstruction of their full original shape.

From each one of the pieces are made two replicas. One of them tries to reply a "fake original", with formal irregularities, incomplete conservation and the characteristic fragmentation of archaeological ceramics, the intention is that it can be confused with an original piece. The other is conceived as a replica of each original piece; using a more geometric shape and a continuous decoration. In the case of the bell-beaker vessel, is made a third replica where the white paste characteristic of this type of objects is inserted into the decorated part.

The intention is **to use the replicas to analyze the visual response they generate by eye-tracking** from images of the same presented on the screen to the experimental subjects. **The fake originals were executed for, in the future, to be able to analyze the perceptual effects that generate to the experimental subjects** by holding them in the hand, believing that they are manipulating old pieces, which will require the use of mobile eye-tracking systems and other techniques to evaluate the emotional response. **In these experiment these pieces were not considered**.

• Fragment from the mound Mámoa 3 de Parxubeira, A Coruña (Neolithic initial/medium)	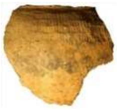
• Vessel *type penha* from the settlement Vinha de Soutilha, Chaves, Portugal (Late Bronce Age)	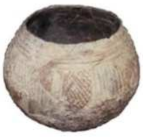
• Bell-beaker vessel from the mound 242, Veiga de Vilavella, As Pontes, A Coruña (Megalithism)	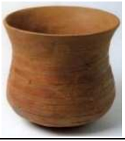
• Fragment of *castreñan* pot from the hillfort Castro de Punta de Muiño do Vento, Pontevedra (Iron Age II, IV-II century b.C.)	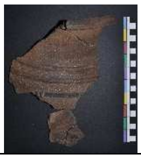
• Fragment of pitcher *type Toralla*, from the hillfort Castro Grande de Neixón, Boiro, A Coruña (Iron Age II, IV-II century b.C.)	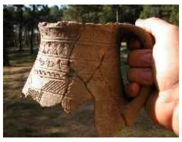

Data sheet

For the realization of the replicas Incipit-CSIC has had the collaboration of the foundation Fundación Terra Termarum (Cuntis, Pontevedra), http://www.castrolandin.es/

Technical team:

- Felipe Criado Boado (Incipit, CSIC)
- Yolanda Porto Tenreiro (Incipit, CSIC)
- Sandra Obarrio Fernández (Fundación Terra Termarum)
- Elena Cerviño Ferrín (Fundación Terra Termarum)
- Jose Luis Villanueva López (Fundación Terra Termarum)

Information about the original pieces

NEOLITHIC VESSEL FROM PARXUBEIRA

<table>
<tr><td>Original</td><td colspan="5">Fragment of edge from M-3 of Mina de Parxubeira (A Coruña). Deposited in the Archaeological and Historical Museum of A Coruña.</td><td>Replicas</td><td>1R(replica) /10 (fake original)</td><td>CCA</td><td>Initial/medium Neolithic.</td></tr>
<tr><td>Origin</td><td colspan="6">The fragment comes from a funerary context, the excavation of the mound Mina de Parxubeira, campaigns 1977-1984. The recovered ceramic material came from the chamber and the corridor, and the set was composed of two fragments without decoration located on the outside of the chamber, six fragments of bell-beaker ceramic, two sheets, a silex arrowhead, an adze, a hoe, a gouge, a arrowhead type palmela and several betilos (sacred stones). (Rodriguez Casal, 1989).</td><td colspan="3">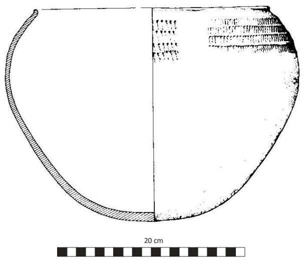
Hypothetical drawinf of the complet shape. (Rodriguez Casal, 1989).</td></tr>
<tr><td rowspan="2">Description</td><td>Manufacture</td><td>Surface finish</td><td>Texture</td><td>Color</td><td>Decoration</td><td>Element</td><td colspan="3" rowspan="2"></td></tr>
<tr><td>Manual modeling</td><td>Smoothed</td><td>Rough</td><td>Light brown, ochre</td><td>Impression</td><td>Shell</td></tr>
</table>

	The vessel is a globular pot of simple profile with slightly rounded and exvased lip. The preserved part allows to reconstruct the profile of the vessel in its upper third. Ceramic baked at low temperature, the overall color is light brown ocher, uneven, some areas darker and some lighter. It has a granitic degreaser with some irregularly distributed medium and large grains that give the surface a rough appearance. The surface finish is a crude smoothing matte-looking that retains the traces of manual modeling, the edge is little regularized. It presents small superficial fissures that evidence an uncontrolled cooking, with abrupt changes of temperature. The decoration is concentrated in the area near to the edge with a continuous band of four horizontal lines made with shell impression; some stretches were deleted to create metopes in which the smooth sections alternate with the decorations. (Use/ Function: funerary vessel)	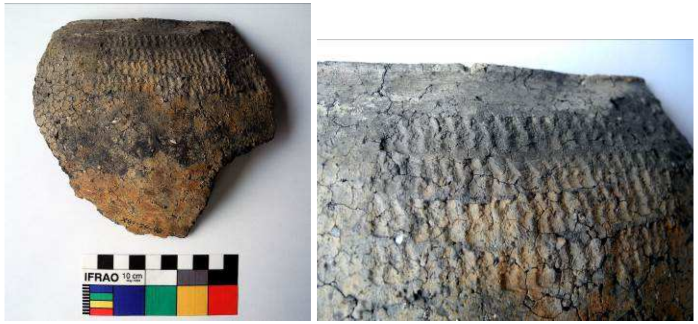 Detail of the original fragment, Archaeological and Historical Museum of A Coruña.

VESSEL TYPE PENHA

<table>
<tr><td>Original</td><td colspan="4">Vessel type “penha” SOJ 23P, it’s preserved almost complete, fixed and rejoin. Deposited in Museo da Regiao Flaviense, Chaves (register number 585).</td><td>Replicas</td><td>2R(replica) /20 (fake original)</td><td>CCA</td><td>End of the III milenium and beginninf of the II milenium b.C. (Late Bronce Age).</td></tr>
<tr><td>Origin</td><td colspan="6">Settlement of Vinha da Soutilha (Mairos, Chaves, Portugal) (Jorge y Soeiro 1981-82; Jorge 1986: 71-312, print IV-LXXXII). This container appear in the called “Nivel III: Sector A (c. 3)”, register with the inventory number “23P” (Print LXXII)</td><td colspan="2" rowspan="3"></td></tr>
<tr><td rowspan="2">Description</td><td>Manufacture</td><td>Surface finish</td><td>Texture</td><td>Color</td><td>Decoration</td><td>Element</td></tr>
<tr><td>Manual modeling</td><td>Smoothed</td><td>Rough</td><td>Brown grey</td><td>Incision and impression</td><td>Punch/burin</td></tr>
</table>

	Spherical ceramic container, quite closed, the part of maximum expansion is just at the half of the total height of the piece. In the section it is possible to realise that the bottom is completely concave and there is no part flattened on which it could be stand. Porous plasticene with generous quartzite aplastic components on the surface. Manually shaped by pressure and coils or coiling technique. The surface finish is smooth with fingers. It is decorated in its upper half, in a band of 10 cm from the edge, with motifs organized into metopes delimited in the part next to the edge by four lines that run along the perimeter of the container, the rest of the piece is smooth. The upper four lines are made by incision with a flat-tipped punch that leaves a mark on U. The metopes are delimited with vertical lines with alternate motives of plait or spike of cereals and reticle. The metopes decoration is made by combining impression and incision.	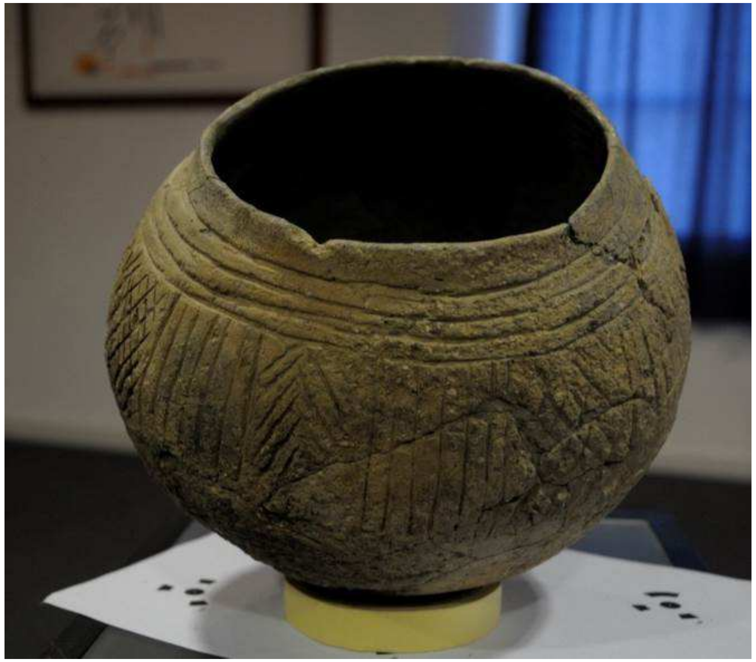<br>Photo of the original piece, Museo da Regiao Flaviense, Chaves.

BELL-BEAKER VESSEL

<table>
<tr><td>Original</td><td colspan="6">Bell-beaker vessel from the mound M242 of Veiga de Vilavella, As Pontes, A Coruña. Collection Santiago de la Iglesia. Depositen in the History I Department, University of Santiago de Compostela. Almost complete container, glued and restored shortly after its recovery in the excavation. Missing since 1992.</td><td>Replicas</td><td>3R(replica) /30 (fake original)/ 3B (replica white paste)</td><td>CCA</td><td>Megalithism</td></tr>
<tr><td>Origin</td><td colspan="6">In the excavation of the mound appeared two bell-beaker vessels. These two bell-beaker vessels are the most famous of Galicia and are currently preserved in the Department of Prehistory of the University of Santiago de Compostela. They were discovered by A. de la Iglesia, and published by D. Luis Pericot in 1927. They have the typical form of wide bands, equidistant, symmetrical and with red earth tone (Criado Boado, 1989).</td><td colspan="4">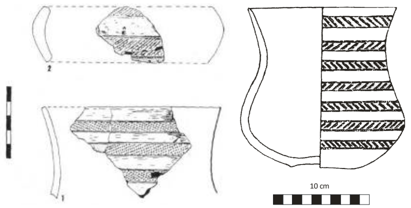
Archaeological drawing of two fragments, schematic drawing of the complete form (Criado Boado, 1989)</td></tr>
<tr><td rowspan="2">Description</td><td>Manufacture</td><td>Surface finish</td><td>Texture</td><td>Color</td><td>Decoration</td><td>Element</td><td rowspan="2" colspan="4"></td></tr>
<tr><td>Manual modeling</td><td>Burnish</td><td>Polished</td><td>Light reddish</td><td>Impression</td><td>Shell</td></tr>
</table>

	Bell-beaker vessel of light reddish paste with decoration of 7 bands with motifs of oblique lines realized by impression of shell, which alternate with smooth bands. Oblique lines are delimited by horizontal lines forming the band/sash. The surface finish is polished, contrasting the gloss of the smooth sashes with the matte appearance of the stippled decorated bands. (Use/Function: funerary vessel).	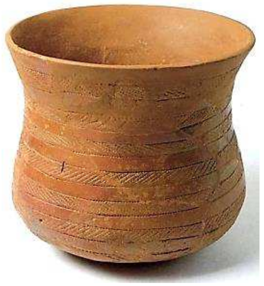 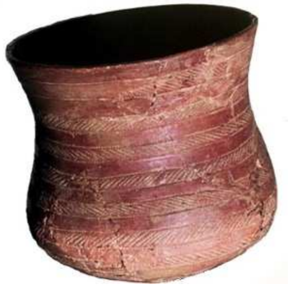 The reconstructed vessel, with glued fragments and reintegration of faults.

CASTREÑAN POT

<table>
<tr>
<td>Original</td>
<td colspan="3">Small fragment of edge and neck of a pot type Cies, from the hillfort called Castro of Punta do Muíño do Vento (Pontevedra). Deposited in the Museum of the Sea of Galicia, Vigo.</td>
<td colspan="2">Replicas</td>
<td>4R (replica)/ 4O(fake original)</td>
<td>CCA</td>
<td>Iron Age II (IV-II century b.C)

Castreñan Culture</td>
</tr>
<tr>
<td>Origin</td>
<td colspan="6">Hillfort called Castro de Punta de Muiño do Vento (Pontevedra), excavation 2002-2003.</td>
<td colspan="2">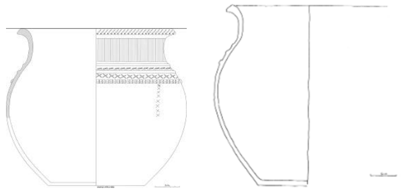
Hypothetical drawing of the complete form and new version with something more height</td>
</tr>
<tr>
<td rowspan="2">Description</td>
<td>Manufacture</td>
<td>Surface finish</td>
<td>Texture</td>
<td>Color</td>
<td>Decoration</td>
<td>Element</td>
<td colspan="2" rowspan="2"></td>
</tr>
<tr>
<td>Manual modeling</td>
<td>Smooth</td>
<td>Smooth</td>
<td>Dark brown</td>
<td>Impression, burnish, plastic</td>
<td>Punch/burin, stamp</td>
</tr>
</table>

	Cooking pot in S shape, a flared rim that finishes in a lip of triangular section, straight neck and rounded body. The decoration is focussed on the upper part of the vessel, on the edge oblique lines stamped, on the neck burnished vertical lines, two delimited bands with three applied cords of rectangular section, two of them smooth and the last thicker decorated by stamp. Inside the two bands, decoration with stamped motifs and under the last cordon vertical lines formed by blades/cross. (Use: Food / Function: Preparation or Conservation / Object: Pot).	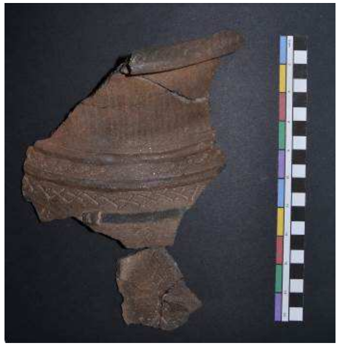 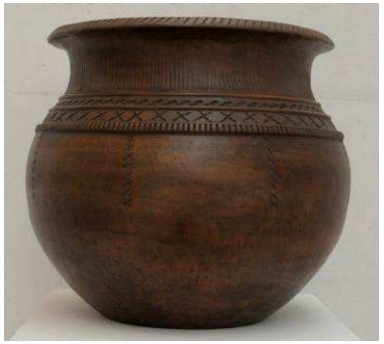 Original fragment preserved and replicated by the Terra Termarum Foundation within the framework of the *Research and Recovery Proyect of Castrexa Ceramic in Galicia*.

CASTREÑAN JUG

<table>
<tr><td>Original</td><td colspan="6">Fragment of the upper third of a pitcher type Toralla, coming from the big hillfort of Neixón, Castro Grande de Neixón, (Boiro, A Coruña). Deposited in the Barbanza Archaeological Center.</td><td>Replicas</td><td>5R (replica)/ 5O(fake original)</td><td>CCA</td><td>Iron Age II (IV-II century b.C.)
Castreñan culture</td></tr>
<tr><td>Origin</td><td colspan="7">Castro Grande de Neixón, (Boiro, A Coruña), excavation 2004.</td><td colspan="3">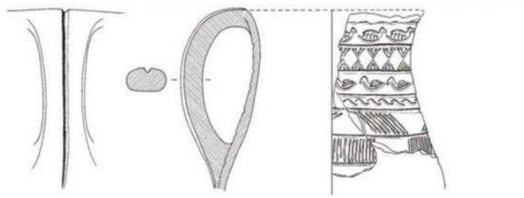
Archaeological drawing of preserved and glued fragments.</td></tr>
<tr><td rowspan="3">Description</td><td>Manufacture</td><td>Surface finish</td><td>Texture</td><td>Color</td><td>Decoration</td><td colspan="2">Element</td><td rowspan="3" colspan="3">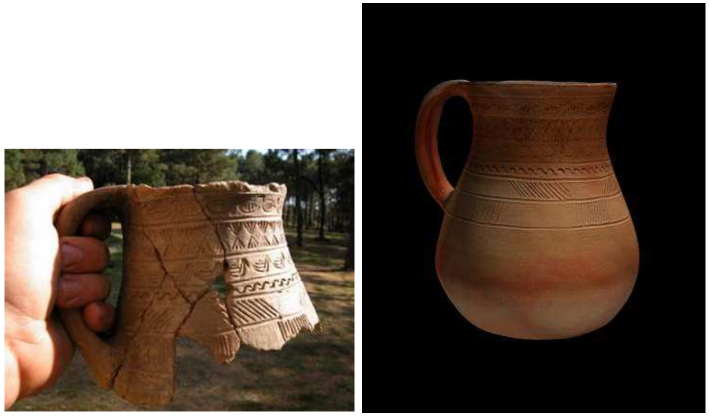
Original fragment and Replica made by Terra Termarum</td></tr>
<tr><td>Manual modeling</td><td>Fine smoothing</td><td>Smooth</td><td>Light brown</td><td>Incision and impression</td><td colspan="2">Punch/burin with rounded tip, estamp</td></tr>
<tr><td colspan="7">S-profile pitcher, modeled manually by the coiling technique, the flared rim ends on a rounded lip. It has a fine smoothing finish and the upper third of the container is decorated with bands of stamped motifs, the bands are limited with incised horizontal lines. The stamp is applied deeply creating a very marked contrast between the decorated part of the container and the smooth part. The finish is a fine smoothing that creates a satin appearance, not polished. The handle, with an oval section, goes from the edge to the transition part of the neck and the pot-belly, has a central groove/fluting made with the same punch used in the decoration. (Use: Food / Function: Presentation / Object: Jug).</td></tr>
</table>

Replication

Graphic documentation

Once the original pieces were selected, it was necessary to obtain the maximum graphic information of the pieces to be able to observe and reproduce the details in the forms, textures, finishes, type of decoration, color.

In the case of the neolithic piece and the *type penha* it was necessary to request permission for reproduction to the museums in which they are deposited to complete the graphic documentation. In the remaining pieces, the graphic documentation already published was used.

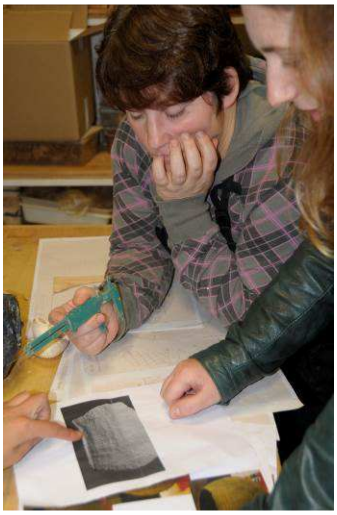
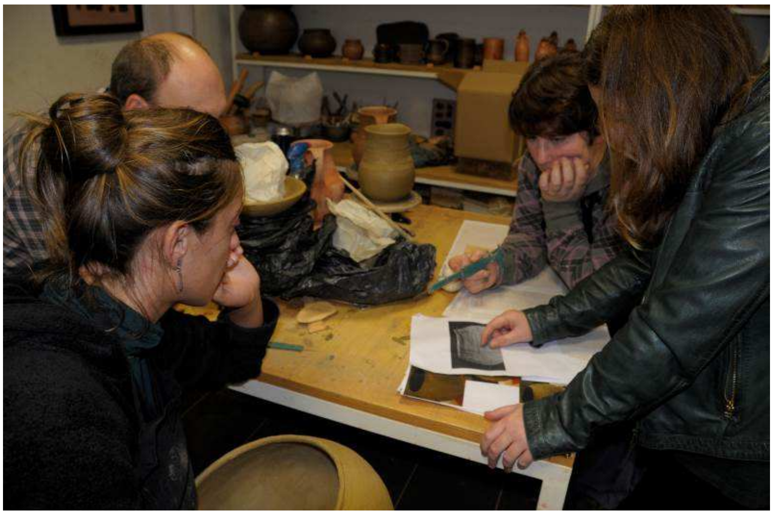

Drawing and detail photos of finishes and decorations.

The fragment of Neolithic vessel of Parxubeira, is deposited in the Archaeological and Historical Museum of A Coruña and forms part of the funds of the Xunta de Galicia, reason why it was necessary to request the permission of the museum and also of the Xunta to be able to realize new photographs of the fragment. General and detailed photos were made especially to determine the element used to make the decoration. From the photos made, the 3D model of the fragment was obtained.

The *type penha* piece from Vinha de Soutilha deposited in the Museum of Chaves also did not have an exhaustive photographic documentation either archaeological drawing, so we request the possibility of completing this information. In this case a complete drawing of the shape and decoration was done as well as a complete sequence of photos that allowed to obtain a 3D model in this case of the complete piece. http://hdl.handle.net/10261/87826.

Once the information of each original piece was collected, Terra Termarum Foundation took charge of the replications, working in a coordinated way with the Incipit to obtain the desired results.

Materials and technology

In order to make the replicas two types of clay are used, of native origin used until today in the traditional pottery. We use one or the other depending on the result that we want to obtain. One of them is called groged (chamotte) red clay coming from Buño of reddish color in fresh, the other clay comes from Gundivós and has ocher color in fresh.

It was intended to reproduce the maximum level of detail appreciable in the original pieces. All the replicas are made by manual modeling, using the coiling technique, only the slow potter's wheel was used to finish the pieces from the Iron Age, as it would be done in the original pieces.

In those pieces in which the technique of manufacture was evident as it was the case of the Neolithic vessel, the presence of the coils was remarked without trying to hide it with a more intense smoothing.

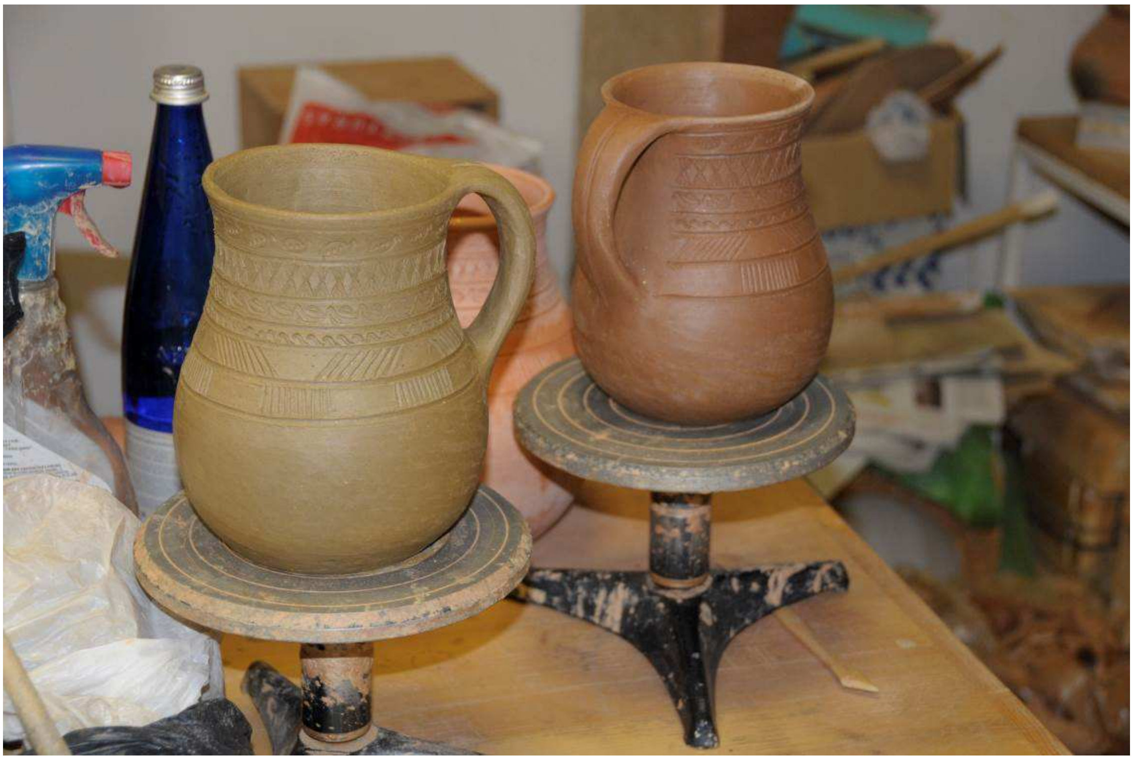

Clays used in replicas.

	Gundivós	Buño
1R		-
1O		-
2R		-
2O		-
3R		-
3O		-
3B		-
4R	-	
4O	-	
5R	-	
5O		

Tª firing	
1R	980^{0}C
1O	indet.
2R	indet.
2O	indet.
3R	980^{0}C
3O	980^{0}C
3B	980^{0}C
4R	980^{0}C
4O	980^{0}C
5R	980^{0}C
5O	980^{0}C

Clay used in replicas and Tª of firing.

Shapes

When making the replicas of the Neolithic vessel and the *castreñan* jug and pot we start with hypothetical reconstructions, since only a fragment of the original pieces is preserved. In these

cases, for drawing the reconstruction of the complete profile, we took into account the general proportions of these vessels, other existing parallels in which the pieces of these typologies are better preserved, and the relation between form and content, in the case of the jug, considering the weight that would have filled with a liquid and supported by the handle.

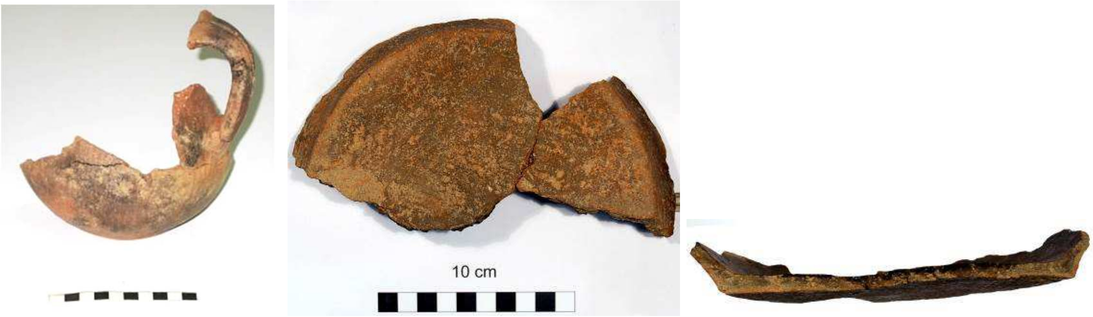

Toralla jug's bottom from the hillfort Castro Neixón and pot bottom from A Lanzada.

Como consecuencia de estas apreciaciones decidimos modificar el modelo de las réplicas de la jarra Toralla procedente de Neixón y de la olla castreña procedente de castro de Punta de Muiño do Vento, que existían en la Fundación Terra Termarum. El resultado de estos cambios es la creación de réplicas con diferente forma pero que parten del mismo fragmento original.

As a result of these appraisal we decided to modify the model of the replicas of the Toralla's jug from Neixón and the *castreñan* pot from the Castro de Punta de Muiño do Vento, which existed in the Terra Termarum Foundation. The result of these changes is the creation of replicas with different shapes but starting from the same original fragment

Surface finish and decoration

In the surface finishes was taken into account the instrument used, the distribution of the finish inside the piece, the contrast between the roughest and smoothest areas and the orientation of the traces.

In the decorative elements, it was tried to imitate the original pieces to the maximum, both in the form of the different elements and in the intensity in case of the stamps in order that their visibility was equal to the original pieces.

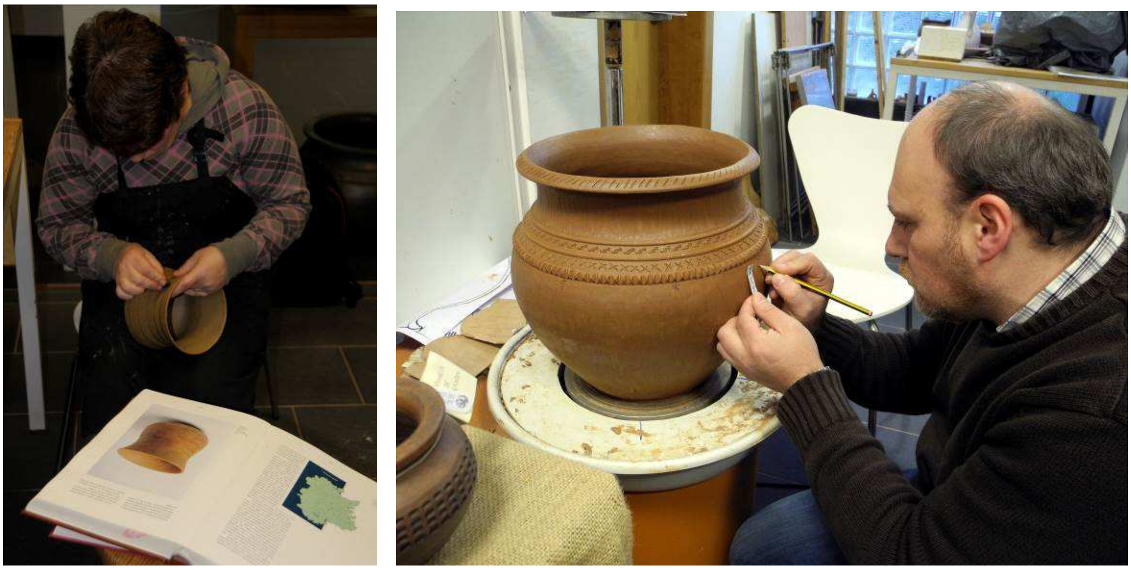

Surface finish and decoration of the replicas.

Firing

Once all the replicas have been made and the drying process is finished, in those replicas in which a final reddish and uniform color (*castreñan* jug and pot) were sought, are firing in an electric kiln. The maximum firing temperature for these pieces is 980^{0}C. To obtain an irregular firing that contributed to give an appearance more similar to the original the rest of pieces are firing in a wood bonfire.

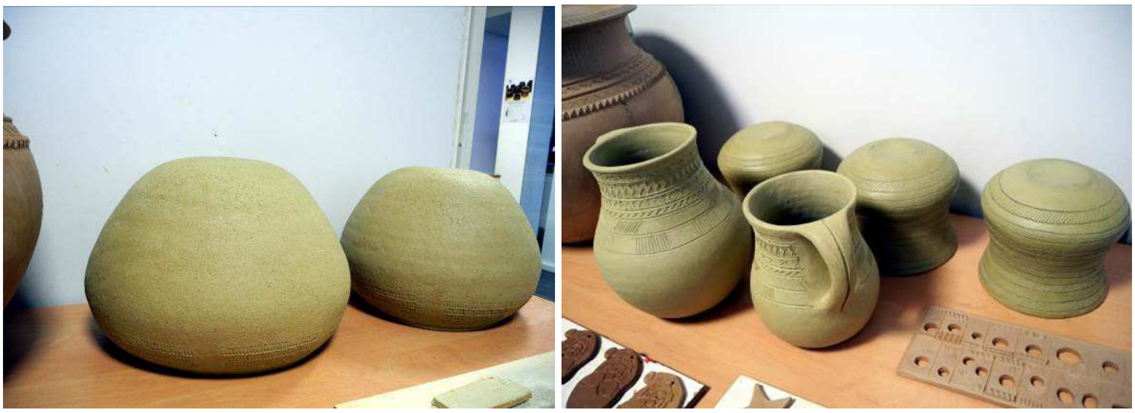

Réplicas during drying.

For the bonfire two holes are made of a 0,50 m deep and 1,5 m in diameter approximately. At the edge of the holes, stones are placed to contain the fire. The two holes are joined with a trench that will serve as an air intake that fuels the combustion. The ovens are made on the ground in an area of clear forest.

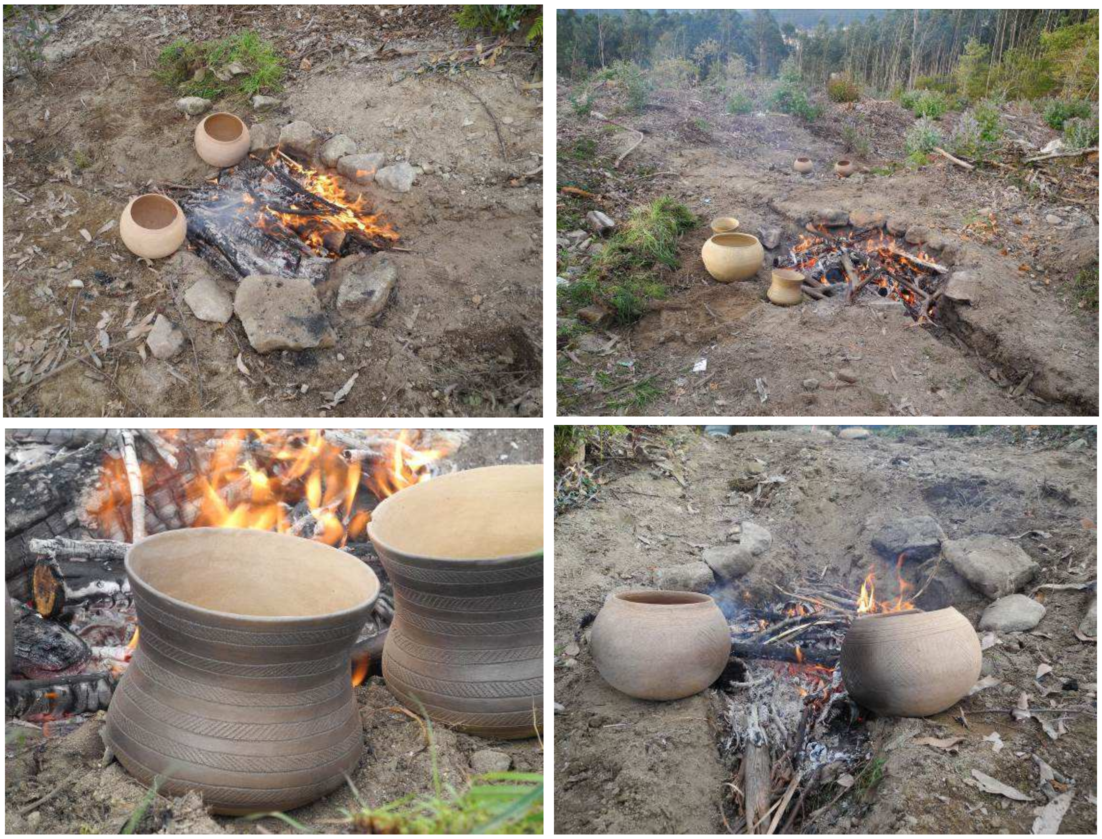

Preparation of the bonfire for the firing of the replicas.

A bonfire is made in each of the holes to create a bed of embers and while the embers are created the pieces which are going to be firing are placed in the edge of the hole so that they

are adapted to the temperature. Approximately one hour after this first fire and with the embers already created, the pieces are placed inside.

In one of the kilns are firing the *type penha* replicas and in another kiln, one of the Neolithic and the bell-beaker replicas. Next, the fire is rekindled by placing firewood over the pieces which remain in combustion for about four hours. After that time the fire is allowed to extinguish and then the pieces are removed and allowed to cool progressively.

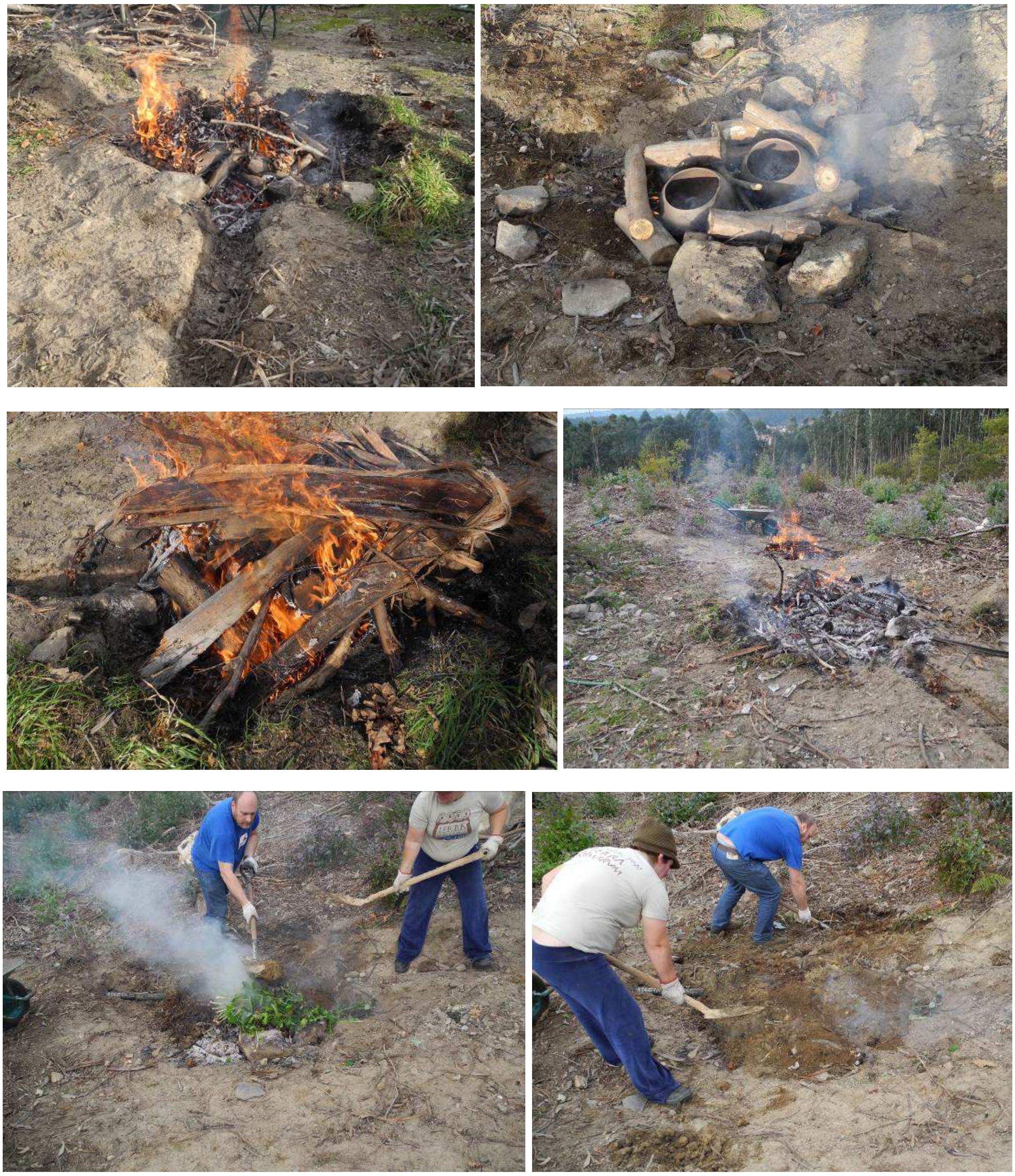

Sequence of images of the firing on bonfire.

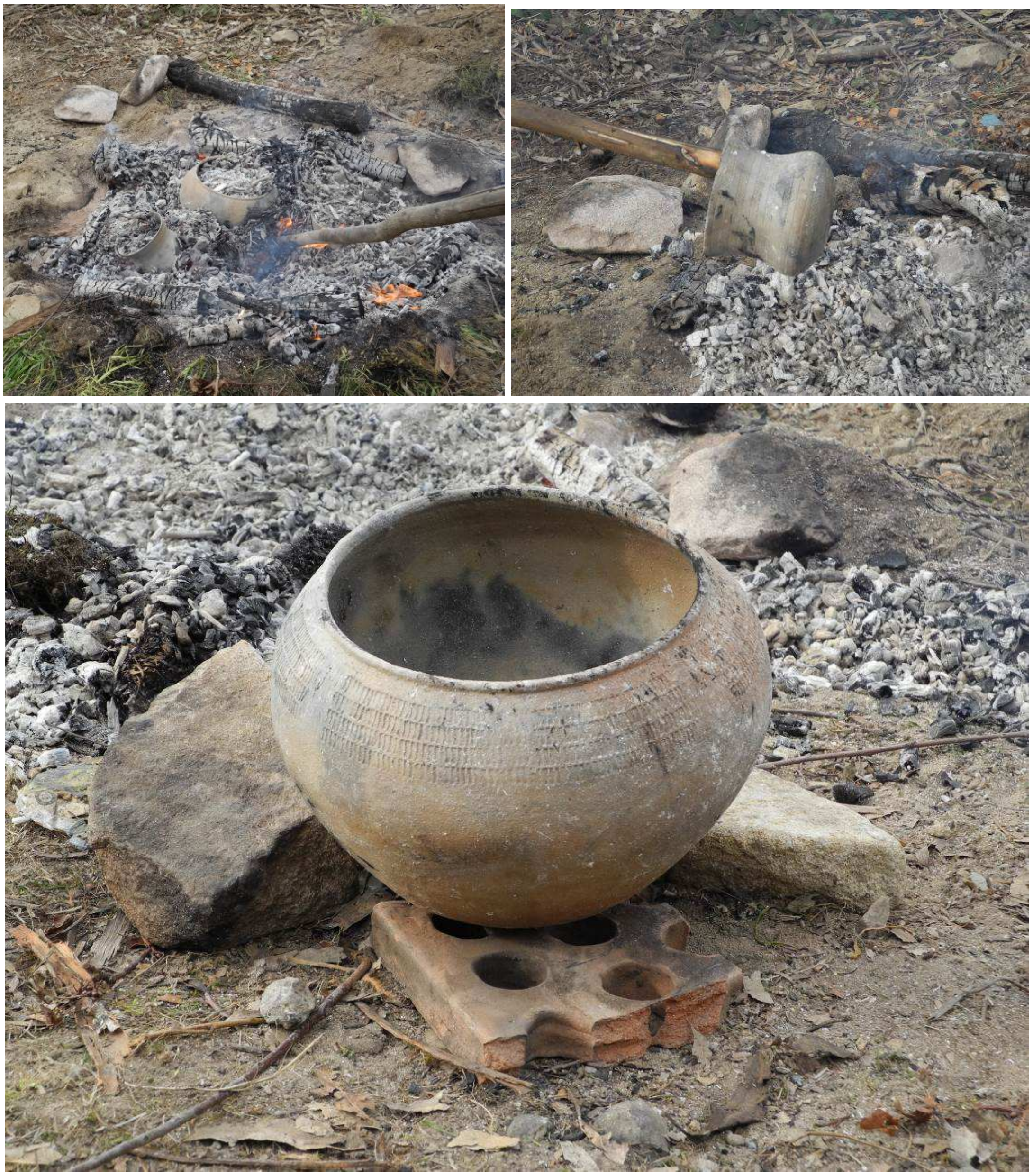

Sequence of images of the firing on bonfire.

In the bonfire where the replicas of the *type Penha* vessel were firing, the fire is extinguished with earth and then covered with green fern branches leaving a central area for the smoke release. It was about creating a reducing firing environment. In this case the pieces are left in the kiln until the next day.

In the case of the *type penha* replicas, a reduction firing is made and the result obtained in terms of color and texture is very similar to the original piece. In the case of the Neolithic replicas, an oxidizing firing is done and the smoky and irregular appearance of the coloration is also very satisfactory. However, in the case of the bell-beaker replicas the smoked appearance moves away from that this type of pieces have, so we decided to finish the surface of the bell-beaker pieces by giving them a more uniform reddish finish by firing them again in the electric kiln, in such a way that we managed to standardize the color.

Final treatment

After firing, a final treatment is given in the wood kiln to the pieces previous firing in the electric kiln to create a smoked effect in the Neolithic replica, and in the *castreñan* jug and pot. In the

case of the *castreñan* replicas manganese oxide is applied previously on the surface and then they are introduced in the kiln so that the oxide penetrates in the pores of the ceramics

In the case of the bell-beaker replica 3B, the decoration is filled with a white paste (ceramichrome, mineral spirit, white color MS19).

Break and Restoration: realization of the "fake originals"

It is uncommon for archaeological vessels to appear complete, usually are found part of the piece, or fragments corresponding to different parts. What is even more exceptional is that in addition to being whole they do not appear fractured. For this reason to make the fake originals we decided to break the pieces and then restore them as would be done with the archaeological objects.

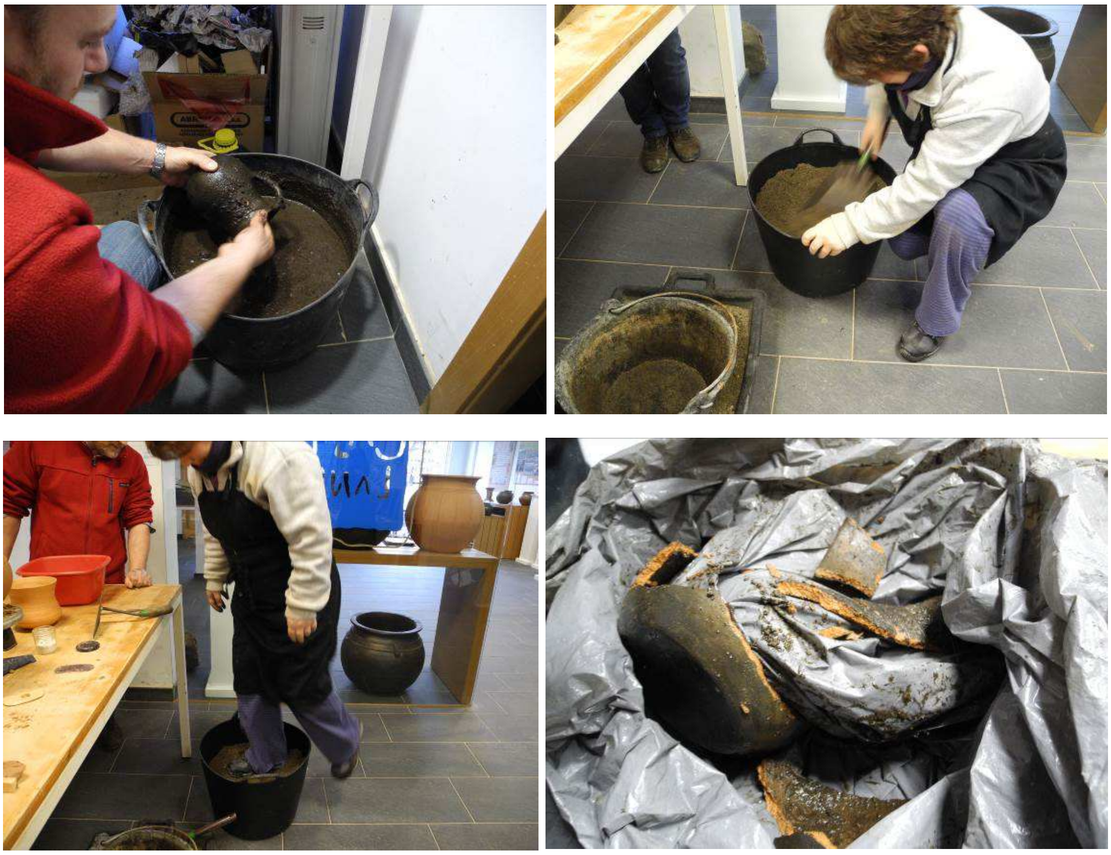

Fake original of the castreña Jug recovered from the clay and rupture.

To cause an abrasion on the outer surface of the ceramics we buried them in mud. After two or three days we caused the vessels to break. In order to try for the break to occur with little loss of material in the fractures, we buried the ceramics wrapped in plastic bag in a bucket with earth. The pieces are placed on the side and with earth also in the interior. Then we exert pressure until we notice the rupture. We remove the earth until recovering the piece that is inside of the plastic bag.

After recovering the fragments from the inside of the bag, the fragments are washed and allowed to air dry. Before beginning the reassembly of the pieces, the fracture lines were sanded so that they have a more rounded appearance and it is not so much noticed that the fracture is recent.

The adhesion of the fragments is done starting from the base of the container and ending at the edge. We use glue Imedio blue band, which makes easy to do the reassembly in the shortest possible time. At the time of gluing the oldest fake originals, the Neolithic and the *type penha*,

we try to make the existing deformation in the pieces be appreciated and even remain more marked in the restored piece. In the *castreñan* and bell-beaker pieces we try to ensure that the deformation in the edge diameter and profiles is the minimum as it happens in the original pieces.

The fracture lines are painted to darken them and the orange color of the interior of the paste does not appear. To achieve a texture and color appearance more similar to that of the ceramic recovered in excavation would be require a prolonged burial period.

In each of the pieces some fragments that are not included in the glued are eliminated. These fragments are kept as a reserve for possible analysis. It was about giving a complete finish but where the elements of the decoration were appreciated discontinuously, and for that we removed some fragments especially of the decorated area of the containers.

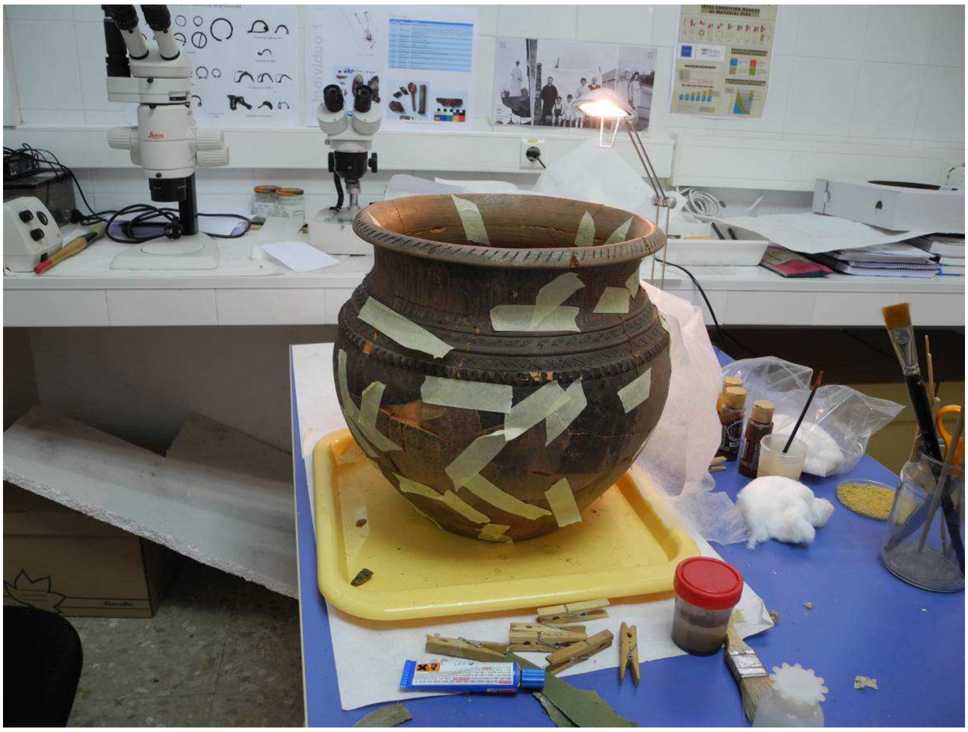

Adhesion of the fake original of the *castreñan* pot

Once the adhesion of the fragments is completed, we reintegrate the faults, the holes left by the fragments removed. For the reintegration of the faults we use Araldit Madera of two components that is modeled to fill the holes and also to reinforce the fracture lines in the case of the Neolithic vessel and the *type Penha*. The reintegration is finished with a smooth finish.

In the Neolithic vessel and in the *castreñan* pot, the reintegration is left unpainted since the color of the putty intones well with the one of the piece. However, in other cases it is necessary to paint the part reintegrated so that it does not emphasize the addition over the original.

Final products

The different products generated through this traditional manufacturing process are described in this section.

Cada pieza se numera con el número del periodo cronológico (o estilo arqueológico) al que pertenece (de 1 a 5 en orden de antigüedad), más la letra R para identificar la “réplica” y 0 para identificar el “falso original arqueológico”.

Each piece is numbered with the number of the chronological period (or archaeological style) to which it belongs (from 1 to 5 in order of seniority), plus the letter R to identify the "replica" and 0 to identify the "fake archaeological original”.

Neolithic vessel of Parxubeira (1R/1O)

In the publications in which is referred this piece, it was said that the decoration was made as a comb, however, after careful observation of the traces and as we could reflect in the photographs seems more likely to have been made with shell type conch. At the time of making the replicas of this piece we decided to make a comb that reproduce the traces left by the shell, since we did not find a shell that totally imitated the original decoration. From the observation of the original fragment we also verify that the decorated band is made continuously and that later some sections are erased creating discontinuous metopes, with decorated and other smooth sections.

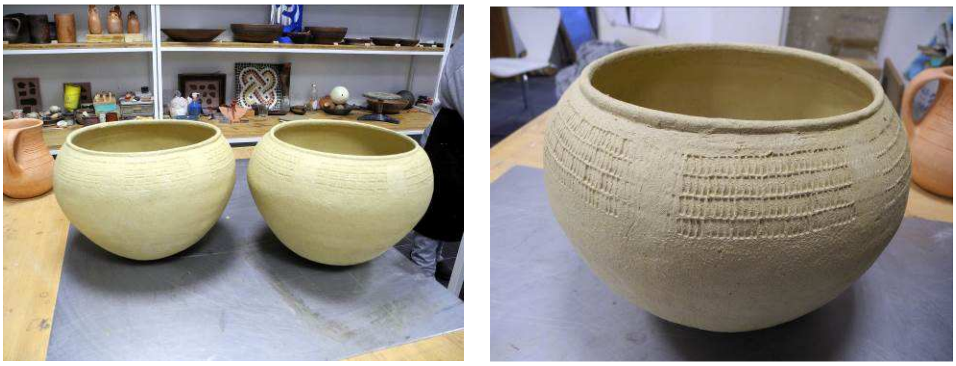

Replica (left) and fake original (right) of the Neolithic vessel during drying.

Replica 1R. The clay used is the ochre clay from Gundivós, it is manually modeled with coils and is given a superficial finish of coarse smoothing, bringing out the aplastic components applying a sponge to create a rough texture. Regarding the shape is about to obtain a piece quite symmetrical, revolutionizing one of the profiles obtained from the archaeological drawing. The mouth of the vessel has a deformation subsequent to the conformation due to the drying process, which has caused it to have an elliptical rather than circular mouth. The decoration is done with the comb that imitates the shell imprint, it is applied over the whole perimeter in a band next to the edge formed by four horizontal lines and some sections are subsequently erased to create a discontinuous decoration. Firing in an electric kiln.

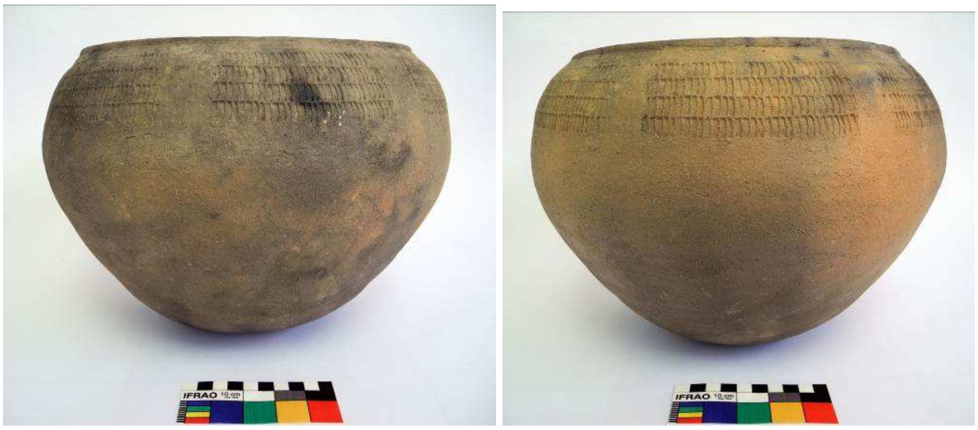

Replica of the Neolithic vessel from Parxubeira 1R.

Fake original 1O. The clay used is the ochre clay from Gundivós. It is manually modeled with coils, surface finishing of coarse smoothing, without hiding the traces of the coils. The decoration is done with a comb, imitating a shell imprint, is applied over the entire perimeter in a band near

the edge and some sections are subsequently erased to create a discontinuous decoration. Firing in an outdoor wood kiln. Buried in mud for 3 days. Breaking of the vessel by burial in sand placed on the side, also filling the inside. The rupture is caused by exerting pressure on the vessel. The fragments are washed to remove the superficial mud remains leaving the earthen remains in the decorated part. Drying the fragments to air for 48 hours in a warm and ventilated environment. Fractures were sanded to cause wear and have a more rounded appearance. Reconstruction of the vessel starting from the base followed by the central part and finally the edge. Small fragments are removed to then reintegrate the gaps with Araldit Wood of two-components resin.

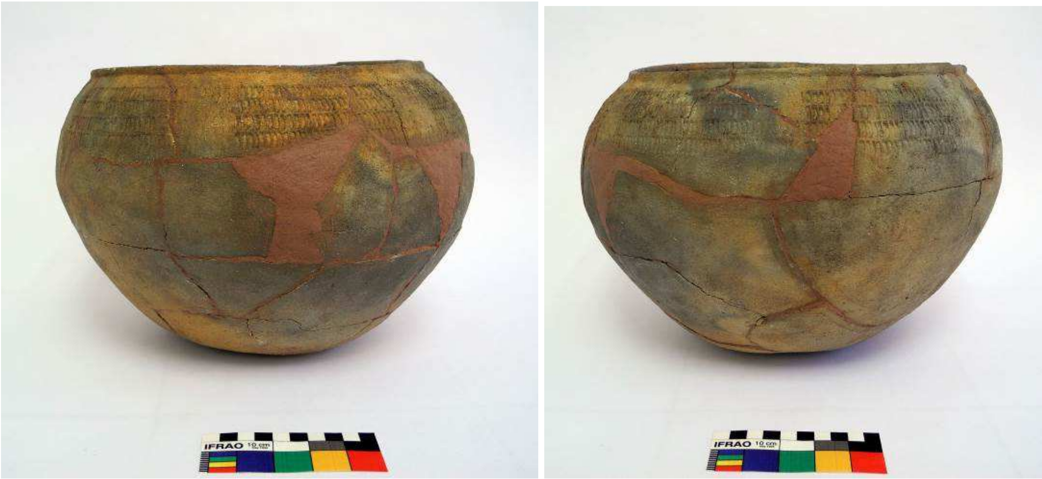

Fake original of the Neolithic vessel from Parxubeira 1O.

Penha vessel (2R/2O)

The original piece, which is in the Museum, is restored and there are parts of the decoration that are not kept complete. As there is not enough documentary information published on the piece, a complete archaeological drawing is made, of the shape and the decoration, as well as a complete photographic report, that allows us to reproduce to the minimum detail the shape, its irregularities and the layout of the decoration.

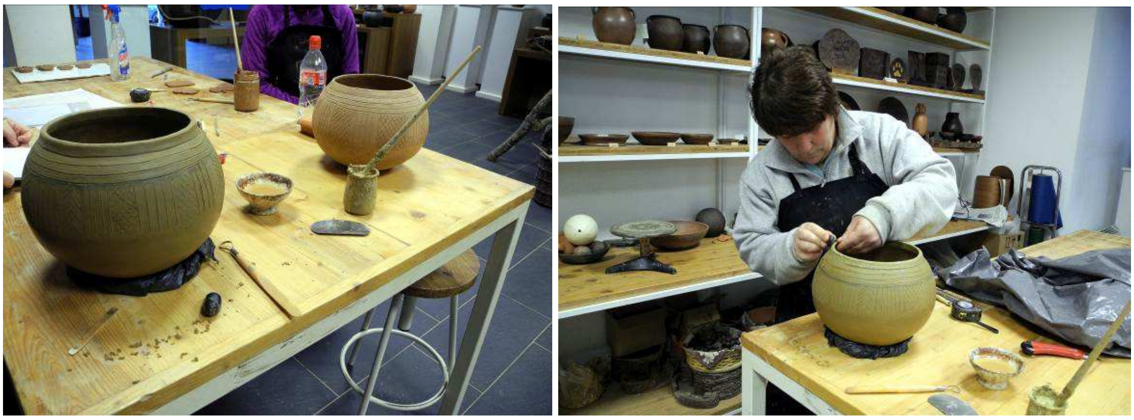
Replicas of the *type Penha* vessel.

Replica 2R. The clay used is the ochre clay from Gundivós. Manual modeling with coils, surface finish of rough smoothing, incised decoration made with a punch/burin and distributed in metopas, is applied over the whole perimeter in a band that goes from the edge to the line of maximum diameter of the vessel. Fired in an outdoors wood kiln.

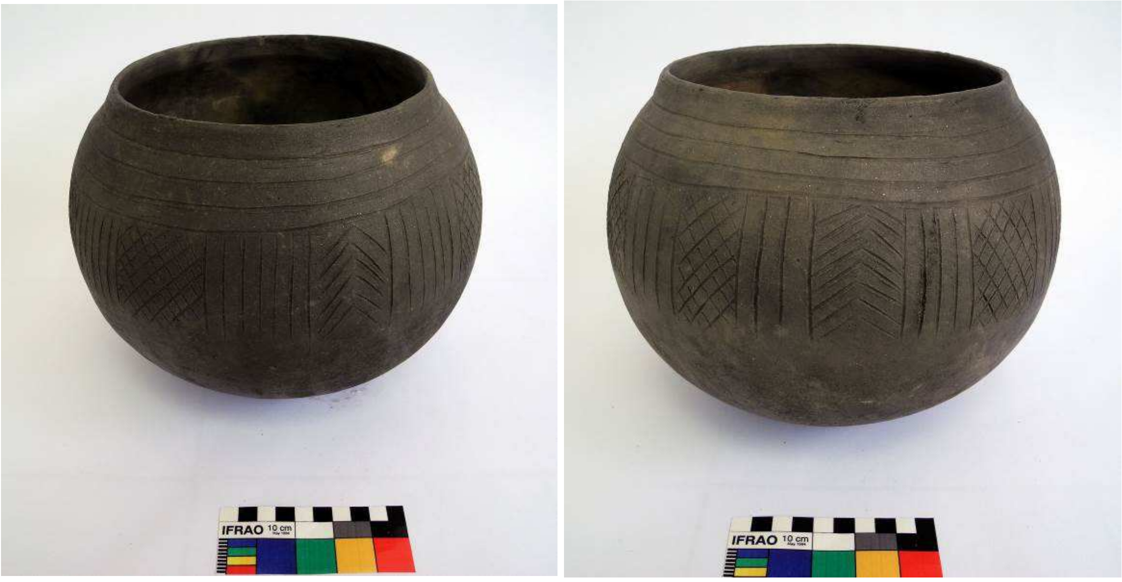

Replica of the *type Penha* vessel, 2R.

Fake original 2O. The clay used is the ochre clay from Gundivós. Manual modeling with coils, surface finish of rough smoothing, incised decoration made with a punch/burin and distributed in metopas, is applied over the whole perimeter in a band that goes from the edge to the line of maximum diameter of the vessel. Fired in an outdoors wood kiln. Buried in mud for 3 days. Breaking of the vessel by burial it in sand placed on the side, also filling the inside. The rupture is caused by exerting pressure on the vessel. The fragments are washed to remove the superficial mud remains leaving the earthen remains in the decorated part. Drying the fragments to air for 48 hours in a warm and ventilated environment. Fractures were sanded to cause wear and have a more rounded appearance. Reconstruction of the vessel starting from the base followed by the central part and finally the edge. Small fragments are then removed to reintegrate the gaps with Araldit Madera two-component resin.

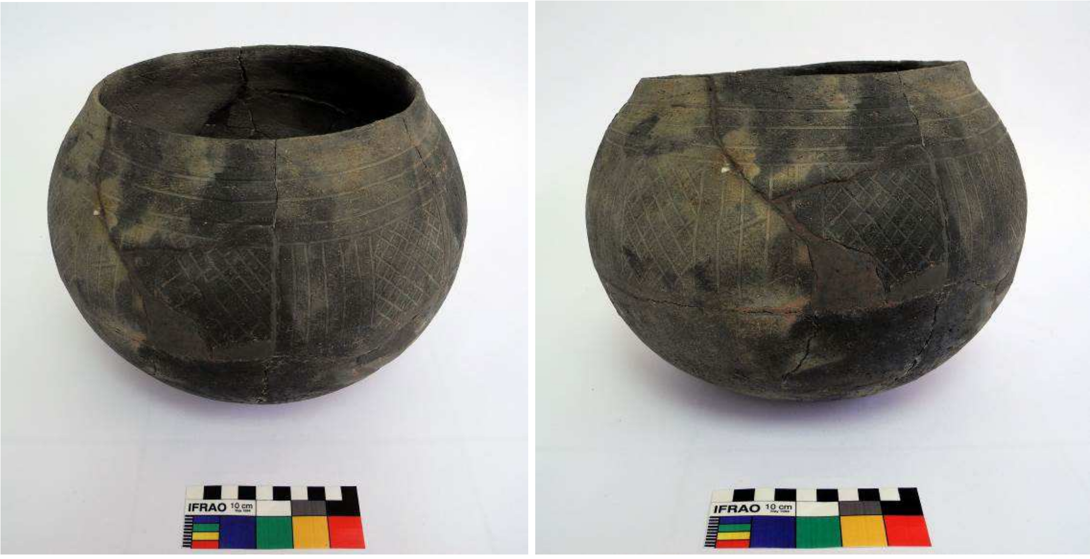

Fake original of the *type Penha* vessel, 2O.

Bell-beaker (3R/3O/3B)

In the replicas of the bell-beaker vessel we try to get the satiny and reddish appearance of this type of vessels. With the firing in wood kiln the pieces had a smoked finish irregularly distributed, so we decided to fire them again at a higher temperature to achieve a more uniform color. In

the decoration of this type of vessel some remains of white paste are conserved in some cases, reason why we decided to make one of the replicas with white paste in its decoration.

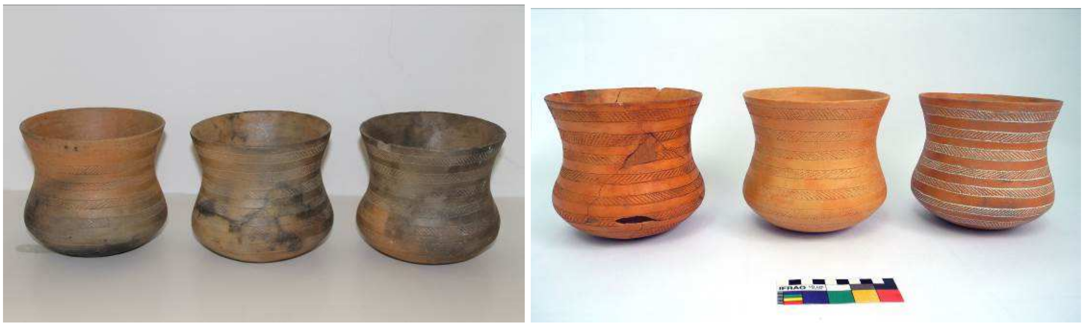

Appearance of the bell-beaker replicas after firing in a wood kiln. Final appearance of the three replicas 3O, 3R, 3B.

Replica 3R. The clay used is the ochre clay from Gundivós. Manual modeling with coils, decoration by comb impression making horizontal bands that alternate with smooth; burnished surface finish. Firing in an outdoor wood kiln. Annealing in an electric kiln to match the color.

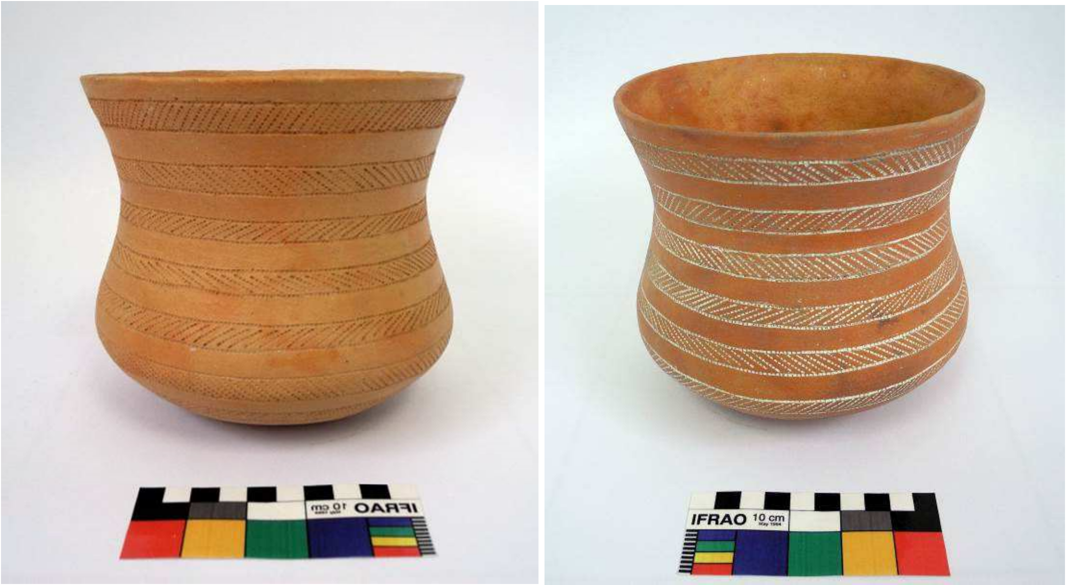

Replica of the bell-beaker vessel 3R and 3B.

Replica 3B. The clay used is the ochre clay from Gundivós. Manual modeling with coils, decoration by comb impression making horizontal bands that alternate with smooth; burnished surface finish. Firing in an outdoor wood kiln. After firing, white paste is applied inside of the decoration and is annealing in the electric kiln to match the color and to fire the white paste.

Fake original 3O. The clay used is the ochre clay from Gundivós. Manual modeling with coils, surface finish of rough smoothing, incised decoration by punch in metopas, is applied over the whole perimeter in a band from the edge to the line of maximum diameter of the container. Firing in an outdoor wood kiln. Buried in mud for 3 days. Breaking of the vessel by burial in sand placed on the side, also filling the inside. The rupture is caused by exerting pressure on the vessel. The fragments are washed to remove the superficial mud remains leaving the earthen remains in the decorated part. Drying the fragments to air for 48 hours in a warm and ventilated environment. Fractures are sanded to cause wear and have a more rounded appearance. Reconstruction of the vessel starting from the base followed by the central part and finally the edge. Small fragments are then removed to reintegrate the gaps with Araldit Wood two-components resin.

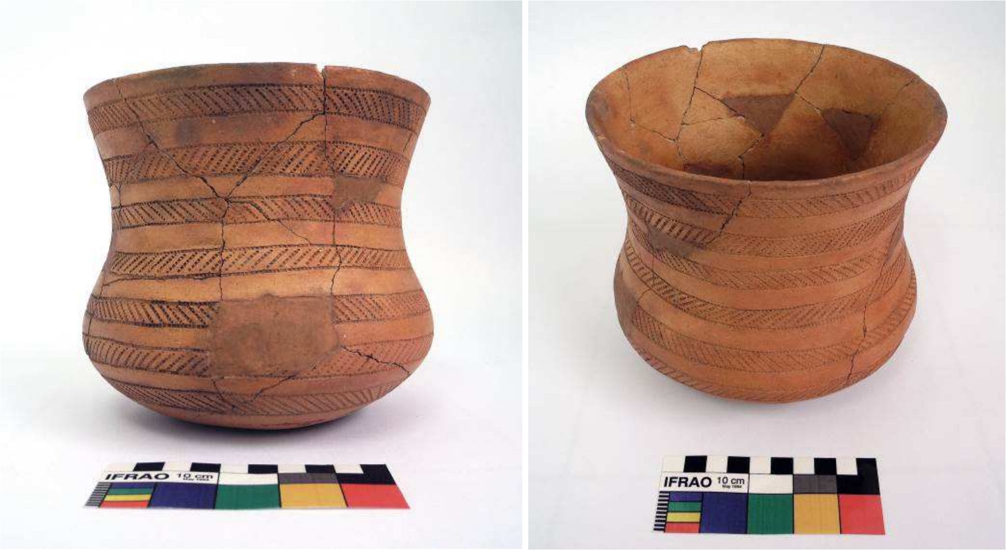

Replica of the bell-beaker vessel 3O.

Castreñan pot (4R/4O)

This is one of the replicas made from a fragment. There was a replica of this piece in the Terra Termarum Foundation, in this new version we decided to give it 1 cm more height.

Replica 4R. The clay used is the red mud from Buño. Manual modeling, with coils, smoothed finish, applied cord, stamp decoration, diamond point decoration on the cord. Firing in electric kiln at 950ºC. Finish after firing, application of manganese oxide with a paint brush.

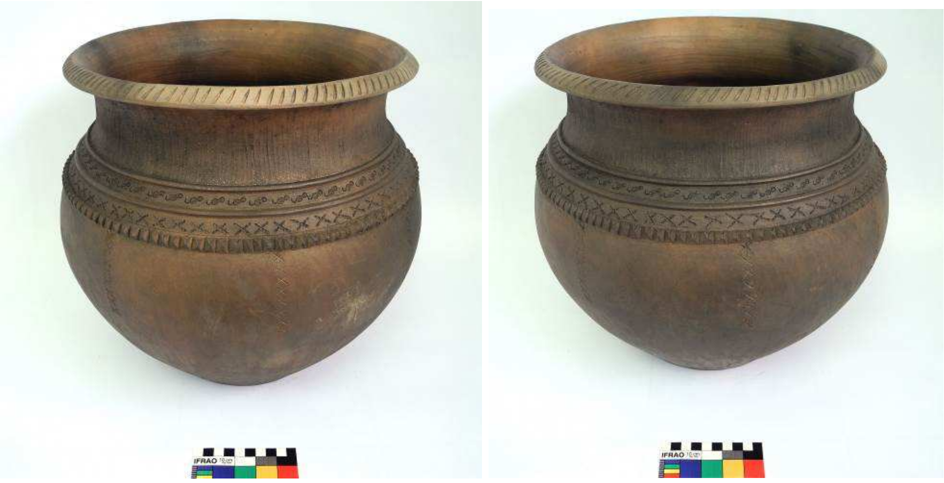

Replica of the castreñan pot 4R.

Fake original 4O. The clay used is the red clay from Buño. Manual modeling, with coils, smoothed finish, applied cord, stamp decoration, diamond point decoration on the cord. Firing in an electric kiln at 950ºC. Finish after firing, application of manganese oxide applied with a paint brush. Buried in mud for 3 days. Breaking of the vessel by burial in sand placed on the side, also filling the inside. The rupture is caused by exerting pressure on the vessel. The fragments are washed to remove the superficial mud remains leaving the earthen remains in the decorated part. Drying the fragments to air for 48 hours in a warm and ventilated environment. Reconstruction of the vessel starting from the base, followed by the central part and finally the edge. Small fragments are removed to then reintegrate the gaps with Araldit Wood two-components resin.

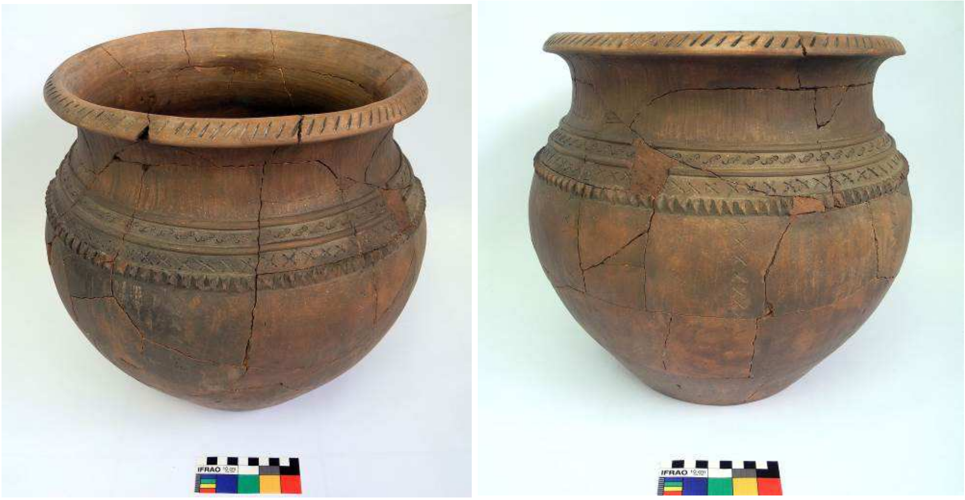

Fake original of the castreñan pot 4O.

Castrexan jar

As in the previous case there was already a replica made from the original fragment but in this case taking into account the weight of the vessel with the content and the carrying capacity of the handle we decided to make it smaller.

Replica 5R*.* The clay used is the red clay of Buño. Manual modelling, with coils, decoration with stamp, handle with central groove. Smooth finish. Firing in an electric kiln at 950ºC.

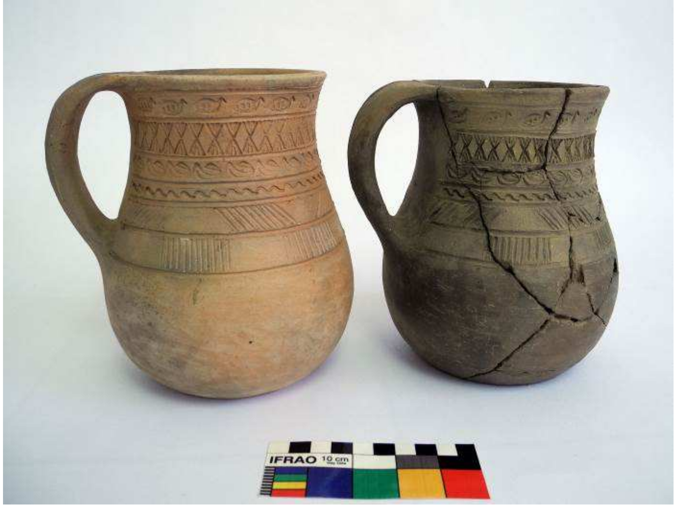

Replica and fake original of the castreñan pot.

Fake original 5O. The clay used is the ocher clay of Gundivós. Manual modeling, with coils, decoration with stamp, handle with central groove. Smooth finish. Firing in electric kiln at 950ºC. Buried in mud for 3 days. Breaking of the vessel by burial in sand placed on the side, also filling the inside. The rupture is caused by exerting pressure on the vessel. The fragments are washed to remove the superficial mud remains leaving the earthen remains in the decorated part. Drying the fragments to air for 48 hours in a warm and ventilated environment. Fractures are sanded to cause wear and have a more rounded appearance. Reconstruction of the vessel starting from the base, followed by the central part and finally the edge. To nuace the color, a few touches of natural ocher diluted in water and applied with a sponge are given on the surface.

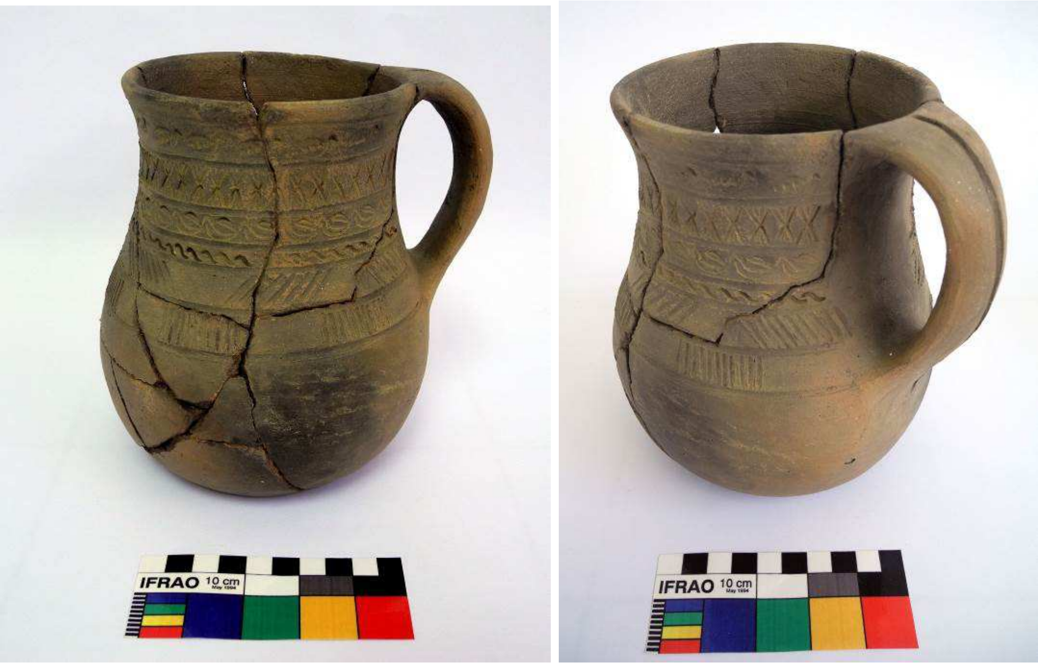

Fake original of the castreñan jug 5O.

References

Ayan, X. M. *Os castros de Neixón*.(Ed. Toxosoutos, Noia, 2005).

Cabrera-Bonet, P., Macías-Ramos, R. & Padilla-Montoya, C. *Diccionario de materiales cerámicos*. (Ministerio de Cultura,Madrid, 2002).

Cortegoso, M. *Proyecto Investigación e Recuperación da Cerámica Castrexa en Galicia*. (Fundación Terra Termarum (no publicado), 2013).

Criado-Boado, F. La cerámica campaniforme en Galicia. *Cuadernos do Seminario de Sargadelos*, **2**. (1982).

Fernández, A. Cerámicas del mundo castrexo del NO Peninsular. Problemática y principales producciones. In *Cerámicas hispanorromanas*. *Un estado de la cuestión*, (eds. Casasaola, B & Ribera, A.) (XXVI Congreso Internacional de la Asociación Rei Cretariae Romanae Fautores, Universidad de Cádiz, 2008)

Prieto-Martínez, P. *Forma, estilo y contexto en la cultura material de la Edad del Bronce gallega: cerámica campaniforme y cerámica no decorada*. PhD thesis, Univ.Santiago de Compostela (1997)

Rodríguez Casal, A. *La necrópolis megalítica de Parxubeira (San Fins de Eirón, Galicia. Campañas arqueológicas de 1977 a 1984)*. (Monografías urxentes do museu, A Coruña, 1984).

Rodríguez Casal, A. *O Megalitismo: a primeira arquitectura monumental de Galicia*. (Biblioteca de Divulgación, USC, 1990).

Rodríguez-Casal, A. El fenómeno tumular y megalítico en Galicia: caracterización general, problemas y perpectivas. *Munibe* **32**, 58-93. (2010).

Rey-Castiñeiras, P.& Soto Arias, P. Estudio preliminar del análisis físico-químico aplicado a la cerámica castreña: vertiente atlántica gallega. *Gallaecia* **21**, 159-176 (2002).

Oliveira, S. & Soeiro, T. Escavações arqueológicas na Vinha da Soutilha (Mairos, 1981). *Portugália* **2-3**, 9-40, (1982).

Oliveira, S. *Povoados da Pré-História recente da região de Chaves-Vila Pouca de Aguiar (Trás-os-Montes Ocidental): bases para o conhecimento IIIº e princípios do IIº milénios A.C. no Norte de Portugal*. PhD Thesis, Universidad de Porto, (1986).
