# Supplementary material for: Coevolution of visual behaviour, the material world and social complexity, depicted by the eye-tracking of archaeological objects in humans
Source: Sci Rep. 2019 Mar 8;9:3985. doi: 10.1038/s41598-019-39661-w (PMC6408451; doi:10.1038/s41598-019-39661-w)
Supplement: Supplementary file 8 — Supplementary Information, Visual display of the reproduction process of replicas [file 41598_2019_39661_MOESM8_ESM.pdf]

Scientific Reports
Paper after editorial revision – SUPPLEMENTARY INFO2: REPRODUCTION PROCESS OF REPLICAS

Coevolution of visual behaviour, the material world and social complexity, depicted by the eye-tracking of archaeological objects in humans

versión 4.0.0, revisión 1 25 de November de 2018 · 22:16
Submission in process: confidential

Authors:

Felipe Criado-Boado[1], Diego Alonso-Pablos[2], Manuel J. Blanco[3], Yolanda Porto[1], Anxo Rodríguez-Paz[1], Elena Cabrejas[1], Elena del Barrio-Álvarez[1], and Luis M. Martínez[2]

Affiliations

[1]Institute of Heritage Sciences (Incipit), Spanish National Research Council (CSIC), Avenida de Vigo s/nº, 15705 Santiago de Compostela, Spain.

[2]Institute of Neurosciences (IN), Spanish National Research Council (CSIC) – Universidad Miguel Hernández (UMH), Campus de San Juan, Sant Joan d'Alacant, Alicante, Spain.

[3] Laboratory of Perception, Faculty of Psychology, University of Santiago de Compostela (USC), Rúa Xosé María Suárez Núñez, s/n, Campus Vida, 15782 Santiago de Compostela, Spain.

*Correspondence to: felipe.criado-boado@incipit.csic.es and l.martinez@umh.es

Electronic Supplementary Material

Supplementary Extended Data include **three documents** and **five video movies**: *Supplementary_Figures*, that incorporates the Extended Data displays (18 figures plus one table) of the Methods section; detailed archaeological and technical information about the pots and the process of experimental manufacturing of replicas used in Experiment 1 (*Supplementary_Info1_Execution-of-experimental-Pots-Replicas*, plus a Powerpoint of the experimental manufacturing process of the pots for Experiment 1 in *Supplementary_Info2_Reproduction-process-of-Replicas*. There are also some movies SI3-7: videos with the total visual movements of 61 experimental subjects for Experiment 1 (Movie SI3 for pot 1, and so on).

Supplementary_Info2_Reproduction-process-of-Replicas

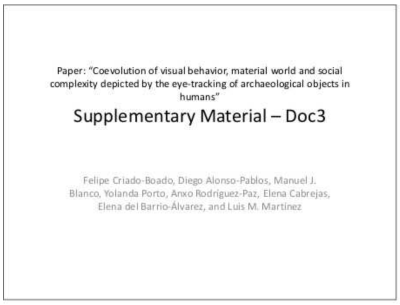

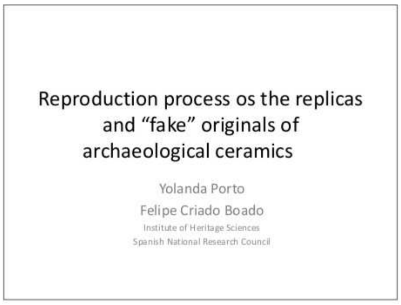

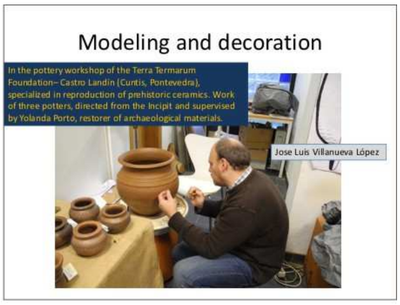

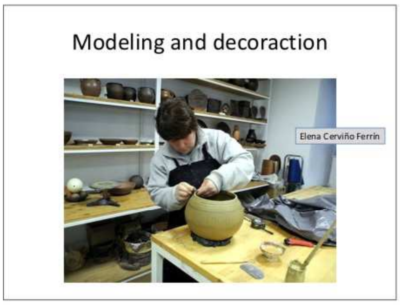

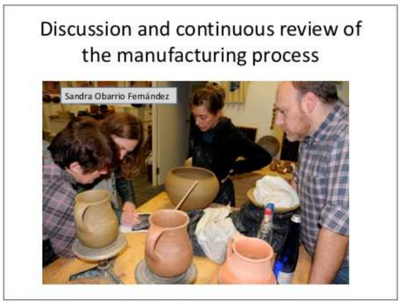

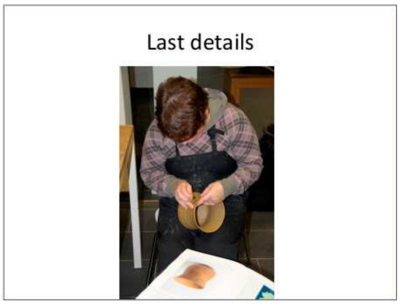

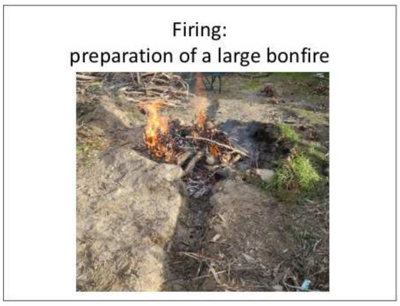

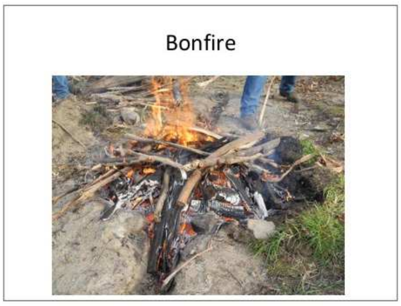

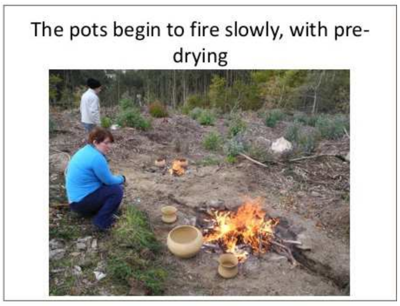

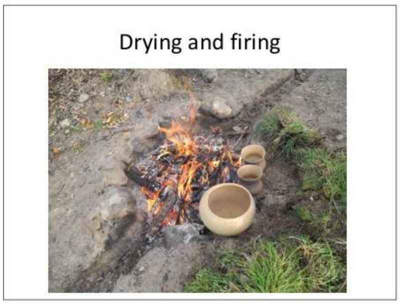

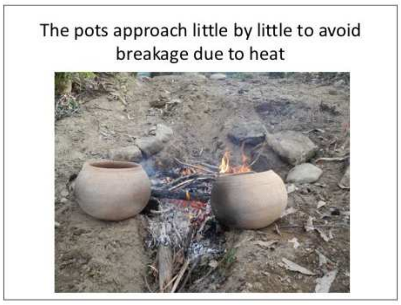

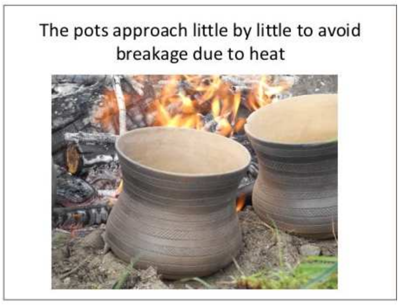

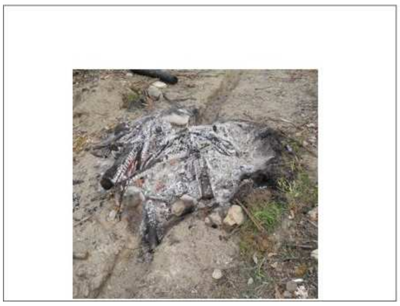

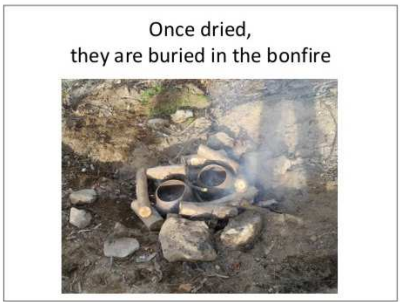

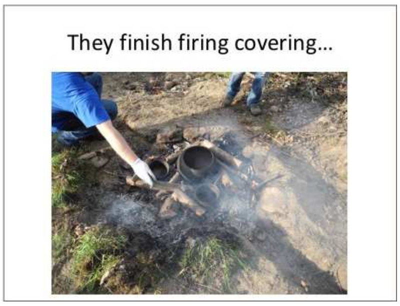

… the bonfire and pots with ashes

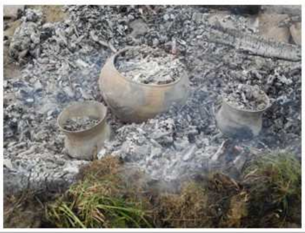

…and finally with leaves…

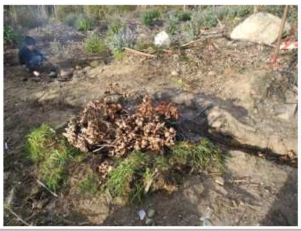

… and soil (to make a firing with oxigen reduction, which will give to the pottery its characteristic original dark tonality)

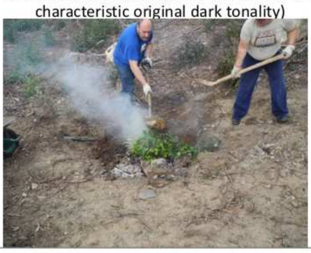

End of firing

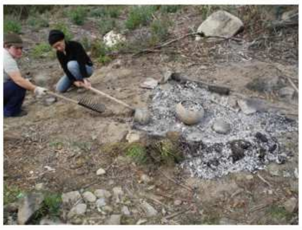

Extraction

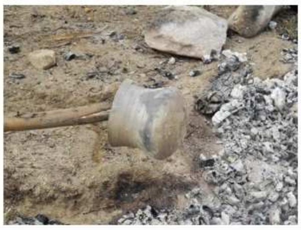

Simulation of use and residues

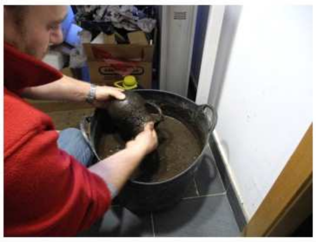

At this point, the replies are finalized (R)
And begins the elaboration of the fake original (O)

Replica of original pot in phase of use (R)

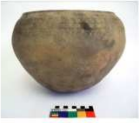

Replica of *fake archaeological pot* (O)

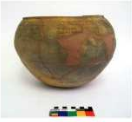

Artificial rupture reproducing archaeological conditions

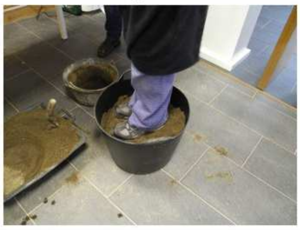

Broken pot

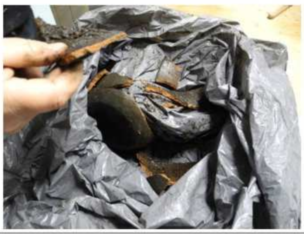

Remounted and recomposition to make a fake original

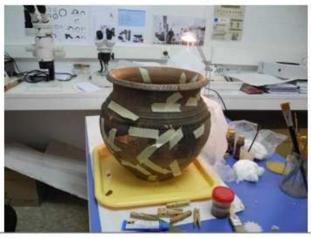

Remounted and recomposition to make a fake original

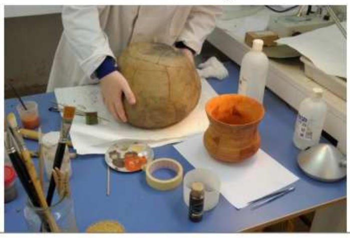

Remounted and recomposition to make a fake original

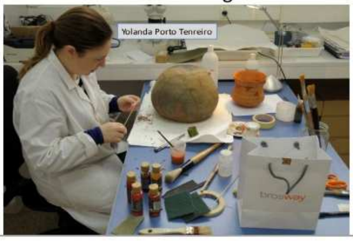

End of the fake original process

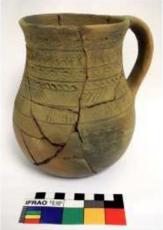

Final result

It has been produced 11 replicas of 5 pots of 5 diferent chronological periods:

Period	Chronology	Result
Neolithic Medium	3500-3000 b.C.	Replica
		Fake original
Final Neolithic	2700-2500 b.C.	Replica
		Fake original
Bronce Age	2100-1900 b.C.	Replica
		Fake original
		Replica with white paste
Iron Age 1	800-400 b.C.	Replica
		Fake original
Iron Age 3	250- 0 b.C.	Replica
		Fake original
